# Cdc73 suppresses genome instability by mediating telomere homeostasis

**DOI:** 10.1371/journal.pgen.1007170

**Published:** 2018-01-10

**Authors:** Rahul V. Nene, Christopher D. Putnam, Bin-Zhong Li, Katarina G. Nguyen, Anjana Srivatsan, Christopher S. Campbell, Arshad Desai, Richard D. Kolodner

**Affiliations:** 1 Ludwig Institute for Cancer Research, San Diego Branch, San Diego, California, United States of America; 2 Department of Medicine, University of California, San Diego, California, United States of America; 3 Department of Cellular and Molecular Medicine, University of California, San Diego, California, United States of America; 4 Moores-UCSD Cancer Center, University of California, San Diego, California, United States of America; 5 Institute of Genomic Medicine, University of California, San Diego, California, United States of America; Columbia University, UNITED STATES

## Abstract

Defects in the genes encoding the Paf1 complex can cause increased genome instability. Loss of Paf1, Cdc73, and Ctr9, but not Rtf1 or Leo1, caused increased accumulation of gross chromosomal rearrangements (GCRs). Combining the *cdc73Δ* mutation with individual deletions of 43 other genes, including *TEL1* and *YKU80*, which are involved in telomere maintenance, resulted in synergistic increases in GCR rates. Whole genome sequence analysis of GCRs indicated that there were reduced relative rates of GCRs mediated by *de novo* telomere additions and increased rates of translocations and inverted duplications in *cdc73Δ* single and double mutants. Analysis of telomere lengths and telomeric gene silencing in strains containing different combinations of *cdc73Δ*, *tel1Δ* and *yku80Δ* mutations suggested that combinations of these mutations caused increased defects in telomere maintenance. A deletion analysis of Cdc73 revealed that a central 105 amino acid region was necessary and sufficient for suppressing the defects observed in *cdc73Δ* strains; this region was required for the binding of Cdc73 to the Paf1 complex through Ctr9 and for nuclear localization of Cdc73. Taken together, these data suggest that the increased GCR rate of *cdc73Δ* single and double mutants is due to partial telomere dysfunction and that Ctr9 and Paf1 play a central role in the Paf1 complex potentially by scaffolding the Paf1 complex subunits or by mediating recruitment of the Paf1 complex to the different processes it functions in.

## Author summary

Maintaining a stable genome is crucial for all organisms, and loss of genome stability has been linked to multiple human diseases, including many cancers. Previously we found that defects in Cdc73, a component of the Paf1 transcriptional elongation complex, give rise to increased genome instability. Here, we explored the mechanism underlying this instability and found that Cdc73 defects give rise to partial defects in maintaining telomeres, which are the specialized ends of chromosomes, and interact with other mutations causing telomere defects. Remarkably, Cdc73 function is mediated through a short central region of the protein that is not a part of previously identified protein domains but targets Cdc73 to the Paf1 complex through interaction with the Ctr9 subunit. Analysis of the other components of the Paf1 complex provides a model in which the Paf1 subunit mediates recruitment of the other subunits to different processes they function in. Together, these data suggest that the mutations in *CDC73* and *CTR9* found in patients with hyperparathyroidism-jaw tumor syndrome and some patients with Wilms tumors, respectively, may contribute to cancer progression by contributing to genome instability.

## Introduction

Gross chromosomal rearrangements (GCRs), such as translocations and deletions, are common in many cancers [1]. DNA repair and DNA damage signaling defects that cause increased rates of accumulating GCRs in model systems like *Saccharomyces cerevisiae* have been identified in sporadic tumors and in inherited cancer predisposition syndromes, suggesting that increased genome instability plays a role in the development of some cancers [2–7]. In addition to defects in DNA metabolism [8,9], defects in transcription are also a source of genome instability. How transcriptional defects cause GCRs is not completely understood, but collisions with the replication machinery, formation of RNA:DNA hybrids, and/or transcription-associated homologous recombination (HR) are potential mechanisms [10,11].

Recently we identified *CDC73* in a large-scale screen for genes that suppress the formation of GCRs in *S*. *cerevisiae* [6]. *CDC73* encodes a subunit of the Paf1 complex, and *CDC73* has been previously implicated as playing a role in maintaining the stability of yeast artificial chromosomes, chromosome transmission fidelity, and suppression of direct repeat HR [12–14]. The Paf1 complex, which is comprised of Paf1, Cdc73, Rtf1, Ctr9, and Leo1, binds to and modifies the activity of RNA polymerase during transcription [15–20]. This complex has been implicated in a variety of processes, including transcription elongation, mRNA 3’-end maturation, histone methylation and ubiquitination, expression of normal levels of telomerase RNA *TLC1* and maintenance of normal telomere lengths [16,21–24], and is conserved among eukaryotes [25]. Somatic mutations in *CDC73* in humans are associated with breast, renal, gastric, and parathyroid cancers [26–28], and germline mutations in *CDC73* cause the cancer susceptibility syndrome hyperparathyroidism-jaw tumor syndrome (HPT-JT) [29,30]. In addition, a small fraction of familial Wilms tumor cases have been attributed to germline mutations in *CTR9* [31]. However, little is known about how *CDC73* and *CTR9* function as tumor suppressors, particularly since mutations in the genes encoding the other members of the Paf1 complex have not yet been linked to the development of cancer.

Here we have investigated how the Paf1 complex acts to suppress genome instability with the goal of shedding light on how the human homolog of *CDC73* may function as a tumor suppressor. We have found that *PAF1*, *CDC73*, and *CTR9* play the most important roles in suppressing the accumulation of GCRs among the genes that encode subunits of the Paf1 complex. Strains with *CDC73* defects appear to have perturbations in telomere maintenance that result in increased GCR rates and that these defects result in synergistic increases in GCR rates when combined with defects in *TEL1* and *YKU80*, which cause other types of defects in telomere maintenance that also result in increased GCR rates. Deletion analysis identified a 105 amino acid region of Cdc73 that was necessary and sufficient for its incorporation into the Paf1 complex, nuclear localization, and Cdc73 function. These analyses enhance our understanding of how Cdc73, as a subunit of the Paf1 complex, suppresses genome instability, and provide insights into how its human homolog may function as a tumor suppressor.

## Results

### Analysis of genome instability, transcriptional defects, and silencing defects in Paf1 complex deletion strains suggests a model for the Paf1 complex

**Fig 1:**
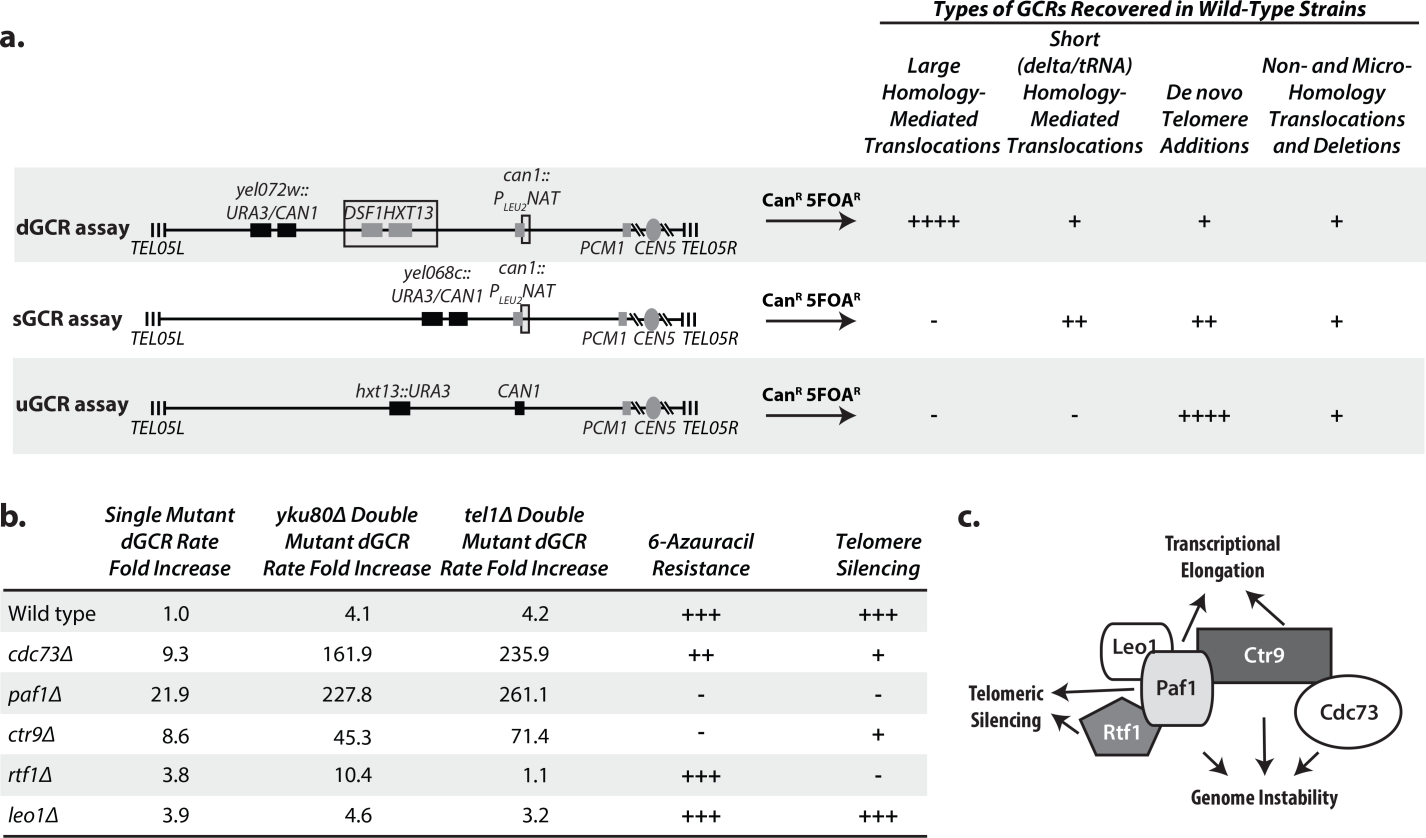
Defects in the genes encoding Paf1 complex subunits affect the suppression of GCRs, transcription elongation, and telomeric silencing to varying degrees. **a.** The dGCR, sGCR, and uGCR assays involve selection against the *CAN1* and *URA3* genes placed on the terminal non-essential region of chrV L. Breakpoints must occur between the most telomeric essential gene, *PCM1*, and the *CAN1* and *URA3* genes. The dGCR assay primarily selects GCRs mediated by non-allelic HR between the *DSF1/HXT13* segmental duplication (boxed) and regions of divergent homology on chrIV L, chrX L, and chrXIV R. The sGCR assay contains a portion of chromosome III containing the *SUP53* tRNA and ~100 bp fragment of *YCLWdelta5* at the *can1*::*P*_*LEU2*_*-NAT* insertion (boxed, also found in the version of the dGCR assay used here) and allows selection of HR-mediated rearrangements that target many tRNA and Ty-related sequences in the *S*. *cerevisiae* genome as well as nonhomology- and microhomology-mediated translocations, interstitial deletions, and *de novo* telomere addition-mediated GCRs. The uGCR assay contains no sequence homology within the breakpoint region and allows selection of nonhomology- and microhomology-mediated translocations, interstitial deletions, and *de novo* telomere addition-mediated GCRs. The number of “+” symbols indicates the relative importance of different types of GCRs in each GCR assay observed in wild-type strains. **b.** The table summarizes the effects of deletion of the genes encoding Paf1 complex subunits on the dGCR rate as single mutations and as double mutation combinations with *tel1Δ* or *yku80Δ* mutations (S1 Table), resistance to 6-azauracil (S1A Fig), and telomeric silencing (S1A Fig). “+++” corresponds to wild-type, “–” corresponds to a severe defect and “++” and “+” correspond to intermediate defects. **c.** Model of the general structure of the Paf1 complex based on cryoelectron microscopy results [81], which illustrates Paf1 as a subunit that facilitates the function of the other subunits, as indicated.

Because we previously identified *CDC73* as a genome instability suppressing (GIS) gene [6], we tested if other genes encoding subunits of the Paf1 complex suppressed the formation of GCRs selected in the duplication-mediated GCR (dGCR) assay (Fig 1A). The *cdc73Δ*, *ctr9Δ*, and *paf1Δ* single mutations caused the largest increases in dGCR rate (9–22 fold), and the *leo1Δ* and *rtf1Δ* single mutations caused small increases in the dGCR rate (3–4 fold; Fig 1B; S1 Table). As will be discussed in detail below, we found that the *cdc73Δ* mutation caused a synergistic increase in the dGCR rate when combined with *yku80Δ* or *tel1Δ* mutations (Fig 1B) and tested if the *yku80Δ* or *tel1Δ* mutations synergized with deletions of other Paf1 complex genes. Similar to the effects of the single mutations, the *cdc73Δ*, *ctr9Δ*, and *paf1Δ* mutations caused strong synergistic increases in the dGCR rate when tested in combination with either a *yku80Δ* or *tel1Δ* mutation whereas the *rtf1Δ* and *leo1Δ* mutations did not cause a synergistic increase in the dGCR rate in combination with either a *yku80Δ* or *tel1Δ* mutation relative to the respective single mutations (Fig 1B; S1 Table). Interestingly, the mutations that caused the strongest increases in GCR rates in these experiments, *cdc73Δ*, *paf1Δ* and *ctr9Δ*, caused the largest decreases in telomere lengths and TLC1 levels along with causing strong defects in telomere gene silencing (see below), whereas the mutations that caused little if any increases in GCR rates in these experiments, *rtf1Δ* and *leo1Δ*, also caused the smallest decreases in telomere lengths and TLC1 levels [32].

Given the differences in the roles of the Paf1 complex subunits in suppressing the accumulation of GCRs, we also tested if transcriptional elongation defects, which are caused by Paf1 complex defects [33–35], might correlate with the increased GCR rates in mutant strains. We measured transcriptional elongation defects that result in sensitivity to 6-azauracil, which depletes cellular rGTP levels [36]. Deletion of *PAF1* or *CTR9* caused strong sensitivity to 6-azauracil, deletion of *CDC73* caused weaker sensitivity, and deletion of *RTF1* or *LEO1* caused no sensitivity (Fig 1B; S1A Fig). These results are in accord with the results of studies employing other transcriptional elongation assays [33–35]; however, it should be noted that the magnitude of the effect caused by defects affecting the Paf1 subunits, including Cdc73, varies between the transcriptional elongation assays used, and 6-azauracil sensitivity assays can show strain-to-strain variation [37].

Strains with deletions of *PAF1* or *RTF1* have defects in the silencing of telomere-proximal genes (*CDC73*, *CTR9* and *LEO1* were not tested) [23], which has been termed the telomere position effect (TPE) [38] and deletions in *PAF1*, *CTR9*, *RTF1*, and to a lesser extent *CDC73*, but not *LEO1* cause defects in the histone H3 modifications required for gene silencing including TPE [23,39–41]. To determine if TPE defects correlated with increased GCR rates, we measured TPE by monitoring the survival of strains with a telomere-proximal *URA3* gene in the presence of 5-fluoroorotic acid (5FOA), which is toxic to strains expressing *URA3*. Deletion of *PAF1* and *RTF1* caused the greatest loss of TPE (Fig 1B; S1A Fig), whereas milder TPE defects were observed in *cdc73Δ* and *ctr9Δ* strains, and no TPE defect was observed in the *leo1Δ* strain. The stronger TPE defects caused by the *paf1Δ* and *rtf1Δ* mutations are consistent with the known role of Paf1 and Rtf1 in the specific recruitment of histone modifiers [23,39]. To verify that the TPE defects in the *cdc73Δ* strain were due to loss of telomere silencing and not due to induction of ribonucleotide reductase, which accounts for the apparent TPE defect in *pol30-8* and *cac1Δ* strains [42], we tested the 5FOA sensitivity of the *cdc73Δ* strain in the presence of sublethal concentrations of hydroxyurea (HU), which rescues the TPE in *pol30-8* and *cac1Δ* strains [42]. Consistent with the results in the absence of HU, growth on 5FOA-containing plates was not restored by addition of HU (S1B Fig). We did not test the deletion of the other Paf1 complex genes because their role or lack of a role in transcriptional silencing is well established [23,39–41] and because *paf1Δ* and *ctr9Δ* strains are HU sensitive [37].

The data presented here along with published data [32] suggest that *PAF1* plays important roles in genome stability, transcriptional elongation, telomere silencing, maintaining *TLC1* levels, and telomere length maintenance. *CDC73* has an important role in genome stability, maintaining *TLC1* levels, telomere length maintenance and a lesser but detectable role in telomere silencing but little if any role in transcriptional elongation. *CTR9* has important roles in genome stability, transcriptional elongation, maintaining *TLC1* levels, and telomere length maintenance, and a role in telomere silencing that was similar to that observed for *CDC73*. *RTF1* has the most important role in telomere silencing, but plays little if any role in genome stability and transcriptional elongation, and lesser roles in maintaining *TLC1* levels, and telomere length maintenance. And *LEO1* plays little if any role at all in the Paf1 complex functions considered here and only a modest role in maintaining *TLC1* levels and telomere length maintenance. These observations suggest a model for the complex in which Paf1 facilitates the functions of the other subunits potentially by mediating recruitment of the complex to the different processes it functions in (Fig 1C), consistent with the results of coimmunoprecipitation experiments in *S*. *cerevisiae* and binding assays performed with human homologs [22,43,44].

### A genetic screen identifies mutations causing synergistic increases in genome instability when combined with a *cdc73Δ* mutation

**Fig 2:**
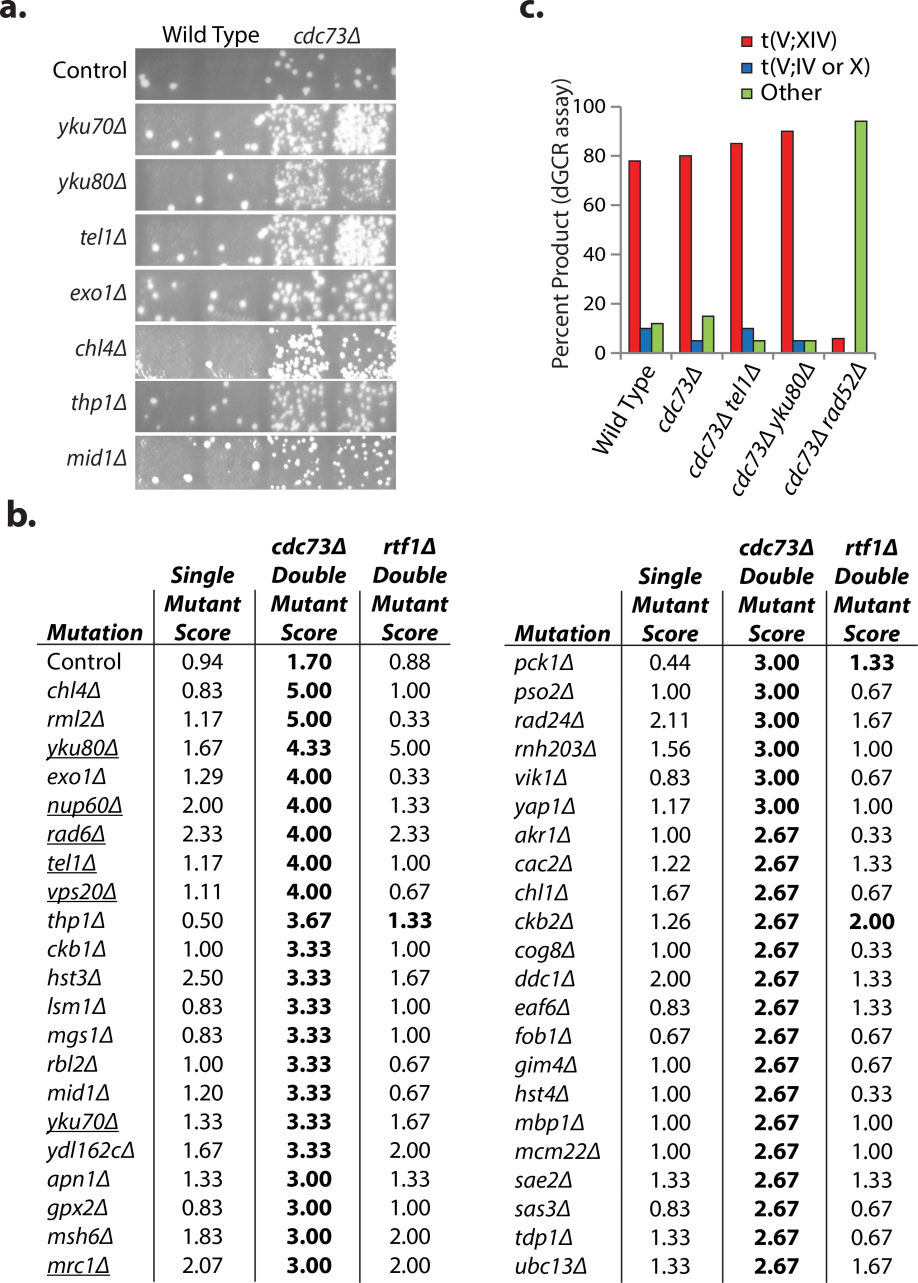
Systematic analysis of *CDC73* as a suppressor of GCRs selected in the dGCR assay. **a.** Sample patches from each of two biological replicates with single and double mutants for genes with mutations that show a synergistic interaction with the *cdc73Δ* mutation. Each papilla corresponds to a GCR event and the greater the number of papillae per patch the greater the GCR patch score, which correlate with increased GCR rates. **b.** dGCR strain scores, which are the average of 3 independent patch scores for mutations that cause increased patch scores when combined with the *cdc73Δ* mutation. The semi-quantitative scoring strategy assigns a number between 0 and 5 to each patch depending on the number of papillae (0: no papillae, 1: 1 to 5 papillae, 2: 6 to 15 papillae, 3: 16 to ~150–200 papillae, 4: distinct papillae that were too many or too close together to count, 5: a lawn of papillae covering the entire patch). A score of 1 corresponds to the wild-type level of GCRs. Interactions with *cdc73Δ* or *rtf1Δ* mutations that resulted in significantly increased patch scores using previously established criteria [6] are displayed in bold. Single mutations causing decreased telomere lengths are underlined [46,47]. **c.** The number of GCRs in the dGCR assay mediated by recombination between *DSF1-HXT13* and *MAN2-HXT17*, t(V;XIV), or between *HXT13* and *HXT15* or *HXT16*, t(V;IV or X), were determined by PCR analysis.

Since *PAF1* and *CDC73* played the largest roles in suppressing GCRs and the *cdc73Δ* mutation caused fewer additional defects, we sought to understand how the Paf1 complex suppresses genome instability by focusing on *CDC73*. We crossed strains containing the dGCR assay and a *cdc73Δ* mutation or an *rtf1Δ* mutation as a control to a 638-strain subset of the *S*. *cerevisiae* deletion collection that contained deletions of known GIS genes and cooperating GIS (cGIS) genes [6,45]. The resulting haploid double mutant strains were scored by patch tests for the increased accumulation of Can^R^ 5FOA^R^ papillae that are a measure of the formation of GCRs relative to the single mutant strains (Fig 2A). Forty-three mutations caused increased strain patch scores when combined with the *cdc73Δ* mutation (Fig 2B); potential suppressive interactions were not investigated as slow growth phenotypes can also cause reduced strain patch scores. Selected interactors causing increased patch scores were verified by quantitative fluctuation assays (S2 Table). Almost none of the mutations that caused increased scores when combined with the *cdc73Δ* mutation interacted with an *rtf1Δ* mutation (Fig 2B), consistent with the more modest effects of *rtf1Δ* on GCR rates (Fig 1B).

### Some mutations affecting telomere homeostasis synergize with a *cdc73Δ* mutation

Among the *CDC73* interactors were 7 genes (*YKU70*, *YKU80*, *TEL1*, *MRC1*, *NUP60*, *RAD6*, and *VPS20)* in which mutations cause shorter telomeres [46–48]. Combined with reports that *cdc73Δ* strains have reduced levels of the telomerase RNA TLC1 [32], these results suggested that defects in telomere homeostasis could be responsible for some of the strong interactions. To extend these results, we focused on *YKU80*, *YKU70*, and *TEL1* because the role of these genes in telomere homeostasis is better understood than the other 4 genes. A *cdc73Δ* mutation showed synergistically increased patch scores when it was combined with either *yku70Δ* or *yku80Δ* mutations, which disrupt the Ku complex and cause both shortened telomeres and non-homologous end joining (NHEJ) defects [49,50]. Quantitative rate measurements demonstrated that the *cdc73Δ yku80Δ* double mutant had a 162-fold increase in dGCR rate as compared to the 4- to 9-fold increase in dGCR rate seen for the respective single mutants (Table 1). In contrast, deletion of *DNL4*, which encodes the DNA ligase required for NHEJ but not telomere maintenance, did not result in a synergistic increase in GCR rates when combined with the *cdc73Δ* mutation (S2 Table) suggesting that the increased GCR rates of the *cdc73Δ yku70Δ* and *cdc73Δ yku80Δ* double mutants do not involve a defect in NHEJ.

**Table 1:** Synergy between *cdc73Δ* and *yku80Δ*, *tel1Δ*, and *exo1Δ* in multiple GCR assays.

*Genotype*	*dGCR assay*		*sGCR assay*		*uGCR assay*	
	*RDKY*	*Can*^*R*^ *5FOA*^*R*^ *Rate*^*†*^	*RDKY*	*Can*^*R*^ *5FOA*^*R*^ *Rate*^*†*^	*RDKY*	*Can*^*R*^ *5FOA*^*R*^ *Rate*^*†*^
Wild-type	7635	8.1 [6.4–15] x 10^−8^ (1)	7964	6.1 [4.3-18] x 10^−9^ (1)	6677	2.27 [1.3–4.8] x 10^−9^ (1)*
*cdc73Δ*	7986	7.54 [3.5–22] x 10^−7^ (9.3)	8407	1.68 [1.1–3.0] x 10^−7^ (28)	8480	1.56 [0.5–2.1] x 10^−8^ (6.9)
*tel1Δ*	8340	3.38 [2.0–4.9] x 10^−7^ (4.2)	8405	7.11 [5.8–8.5] x 10^−9^ (1.2)	6761	4.99 [0.0–9.2] x 10^−9^ (2.2)*
*yku80Δ*	8339	3.29 [1.5–10] x 10^−7^ (4.1)	8406	3.25 [1.3–5.7] x 10^−9^ (0.5)	8006	<6.88 [0.0–7.9] x 10^−10^ (<0.3)*
*cdc73Δ tel1Δ*	8324	1.91 [0.7–3.3] x 10^−5^ (236)	8409	8.00 [3.9–11] x 10^−7^ (131)	8481	2.14 [1.0–7.9] x 10^−7^ (94)
*cdc73Δ yku80Δ*	8323	1.31 [0.7–3.4] x 10^−5^ (162)	8411	1.51 [0.5–2.8] x 10^−6^ (248)	8482	3.73 [2.2–5.2] x 10^−7^ (163)
*tel1Δ yku80Δ*	8467	2.27 [1.3–3.0] x 10^−6^ (28)	8408	1.86 [1.7–2.7] x 10^−8^ (3.1)		n.d.
*pif1Δ*		n.d.	8342	2.49 [1.6–3.5] x 10^−6^ (408)		n.d.
*cdc73Δ pif1Δ*		n.d.	8343	4.36 [0.3–8.4] x 10^−7^ (72)		n.d.
*exo1Δ*	8419	2.21 [1.6–2.7] x 10^−7^ (2.7)	8469	1.09 [0.5–1.6] x 10^−8^ (1.8)		n.d.
*cdc73Δ exo1Δ*	8428	1.02 [0.8–3.5] x 10^−5^ (126)	8470	2.22 [1.2–3.6] x 10^−7^ (36)		n.d.
*tel1Δ exo1Δ*	8464	6.13 [3.2–12] x 10^−7^ (7.6)	8473	1.86 [0.6–3.0] x 10^−8^ (3.1)		n.d.
*yku80Δ exo1Δ*	8463	1.44 [1.2–2.2] x 10^−7^ (1.8)	8472	8.78 [5.2–14] x 10^−10^ (0.14)		n.d.

* GCR rate from [51,52]. The uGCR rate determined using a wild-type uGCR strain constructed in RDKY7635, which is highly related to RDKY6677, was 1.8 [0.7–4.1] x 10^−9^.

^†^Rate of accumulating Can^R^ 5FOA^R^ progeny. The numbers in square brackets [] are the 95% confidence interval limits. The number in parenthesis () is the fold increase relative to the wild-type assay.

n.d., not determined.

Similarly, the *cdc73Δ* mutation showed a strong interaction with a *tel1Δ* mutation in the dGCR assays as measured by patch scores (Fig 2A and 2B), and the *cdc73Δ tel1Δ* double mutant had a 236-fold increase in the dGCR rate (Table 1). *TEL1* encodes a protein kinase involved in the DNA damage checkpoint that also plays a role in maintaining normal telomere lengths such that a *tel1Δ* mutation causes shortened telomere lengths [53,54]. In contrast, mutant strains that contained a *cdc73Δ* mutation in combination with defects in other checkpoint genes either did not have increased dGCR patch scores (*RAD9*, *DUN1*, and *RAD53*) or only had small increases in dGCR patch scores (*MEC3*, *RAD17*, *RAD24*, and *MEC1*), supporting the view that the genetic interaction between the *cdc73Δ* and *tel1Δ* mutations reflects the telomere maintenance defect caused by the *tel1Δ* mutation. The *tel1Δ yku80Δ* double mutant had a 28-fold increase in the dGCR rate (Table 1 and S2 Table) and the *cdc73Δ tel1Δ yku80Δ* triple mutant had a 2024-fold increase in the dGCR rate (S2 Table), consistent with the hypotheses that loss of *CDC73*, *YKU80*, and *TEL1* cause partial defects in different telomere maintenance pathways and that the increased GCR rates that result from combining mutations in these genes may reflect increased telomere maintenance defects.

GCRs selected in the dGCR assay are most commonly generated by non-allelic HR between the *DSF1-HXT13* region on the left arm of chromosome V (chrV L) and divergent homologies on chrIV L, chrX L, and chrXIV R [51]. PCR analysis of GCRs formed in the *cdc73Δ*, *cdc73Δ tel1Δ* and *cdc73Δ yku80Δ* dGCR strains showed that the distribution of GCRs were essentially the same as that from the wild-type strain, despite the >200-fold increase in GCR rate in some of the strains analyzed (Fig 2C). As expected, introduction of the HR-defective *rad52Δ* mutation decreased the dGCR rates of the *cdc73Δ tel1Δ* and *cdc73Δ yku80Δ* double mutants by 45-fold and 25-fold, respectively (S2 Table). In addition, the *rad52Δ* mutation shifted the spectrum of GCRs recovered in the *cdc73Δ* mutant to GCRs that were not formed by non-allelic HR (Fig 2C).

### The *cdc73Δ* mutation reduces the relative efficiency of forming *de novo* telomere addition GCRs

As observed in the dGCR assay, synergistic increases in GCR rates were also observed when the *cdc73Δ* mutation was combined with either the *yku80Δ* or *tel1Δ* mutation in strains containing either the unique sequence (uGCR) assay or the short homology GCR (sGCR) assay (Table 1). Since the sGCR assay selects for a somewhat broader diversity of types of GCRs including *de novo* telomere additions than the uGCR assay and is not dominated by a single type of GCR as compared to the dGCR assay (summarized in Fig 1A), we used the sGCR assay to determine if the absence of *CDC73* altered the distribution of the GCRs formed.

**Fig 3:**
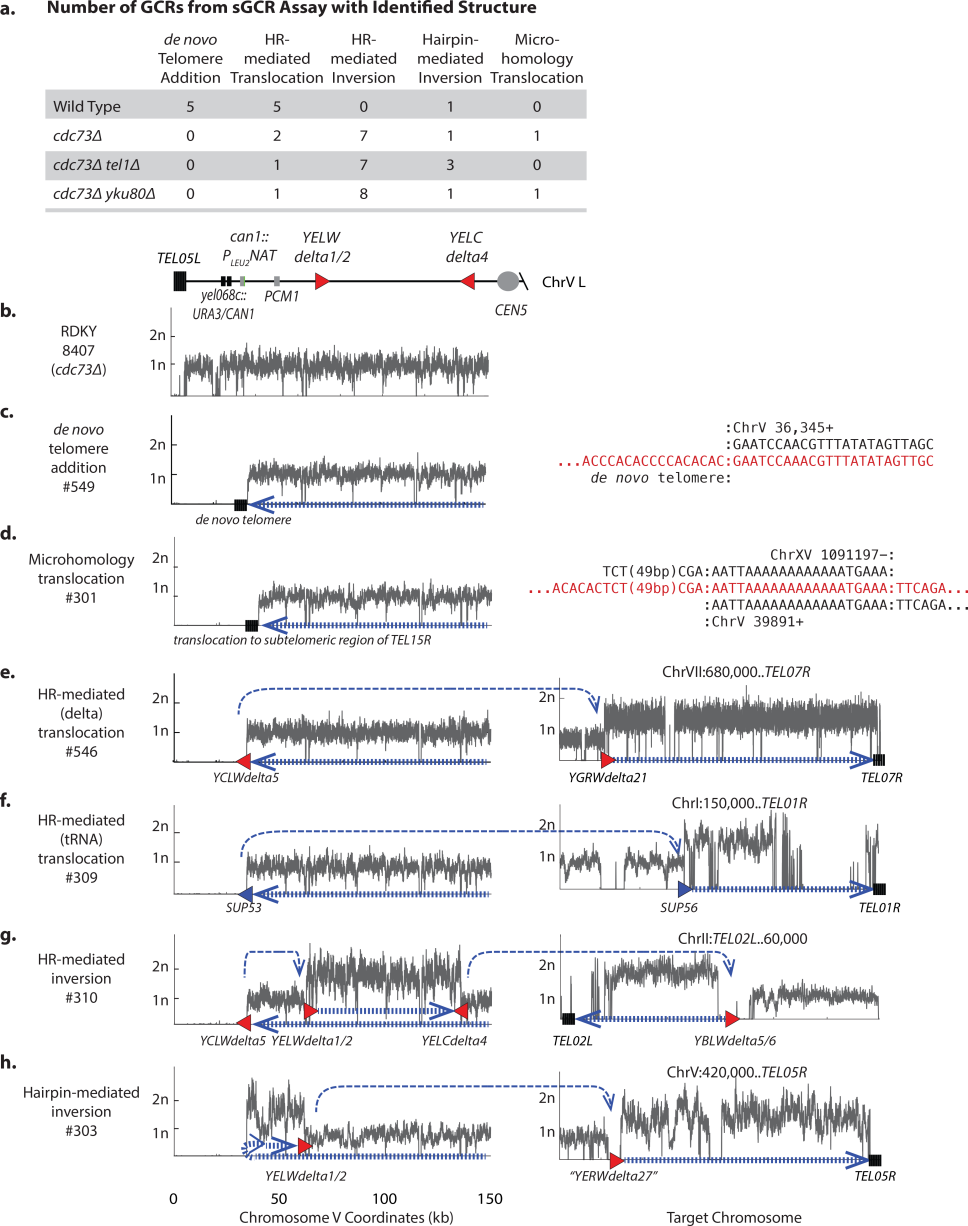
GCRs selected in *cdc73Δ* mutants are characterized by reduced levels of *de novo* telomere additions and increased levels of inverted duplications. **a.** Distribution of different categories of GCRs selected in the sGCR assay. **b.-h**. Copy number analysis of chrV L (left) and the target chromosomes (right) for representative GCRs selected in the sGCR assay based on whole genome sequencing. The thick hashed blue arrow indicates sequences within the GCR; the thin dashed blue arrow indicates connectivity between portions of the GCR that map to different regions of the reference chromosome. Filled triangles are Ty-related (red) or other (blue) duplicated sequences involved in GCR-related HR events. Junction sequences are displayed for rearrangements not associated with copy number changes. **b.** The copy number distribution for the parental strain. **c.** *De novo* telomere addition with a terminal deletion of chrV L and a chrV L/*de novo* telomere junction sequence. **d.** Microhomology-mediated translocation with a terminal deletion of chrV L, and a duplication of a unique sequence from chrXV R terminated by a telomere (*TEL15R*). **e.** Translocation GCR mediated by HR involving the *YCLWdelta5* fragment at the *can1*::*P*_*LEU2*_*-NAT* locus, a loss of all unique sequences telomeric to *can1*::*P*_*LEU2*_*-NAT*, a duplication of chrVII R bounded by*YGRWdelta21* and a telomere (*TEL07R*) at the other end, and characterized by uniquely mapping read pairs that spanned the *YCLWdelta5*/*YGRWdelta21* junction. **f.** Translocation GCR mediated by HR involving the *SUP53* tRNA gene at the *can1*::*P*_*LEU2*_*-NAT* locus had a loss of all unique sequences telomeric to *can1*::*P*_*LEU2*_*-NAT*, a duplication of chrI R bounded at one end by a *SUP53* homolog and a telomere (*TEL01R*) at the other end, and characterized by uniquely mapping read pairs that spanned the *SUP53*/tRNA junctions. **g.** Inverted duplication GCR mediated by an *YCLWdelta5/YELWdelta1* HR-mediated event associated with loss of all unique sequences telomeric to *can1*::*P*_*LEU2*_*-NAT*, duplication on chrV L bounded by *YELWdelta1* and *YELCdelta4*, and duplication of chrII L bounded by a *YBLWdelta5/6* and a telomere (*TEL02L*) mediated by HR between *YELCdelta4* and *YBLWdelta5/6*. The *YCLWdelta5/YELWdelta1* and *YELCdelta4/YBLWdelta5/6* junctions were characterized by uniquely mapping read pairs that spanned each junction. **h.** Hairpin-mediated inverted duplication GCR had a terminal deletion of chrV L, a duplication immediately adjacent to the deletion bounded by an inverted repeat hairpin sequence (light blue arrow) at one end and *YELWdelta1/2* at the other end, and a duplication of chrV R bounded by *“YERWdelta27”* and a telomere (*TEL05R*) mediated by HR between *YELWdelta1/2* and *“YERWdelta27”* characterized by uniquely mapping read pairs that spanned the *YELWdelta1/2*/*“YERWdelta27”* junction.

We analyzed 1 parental strain and 11 independent GCR-containing isolates by paired-end next-generation sequencing for the wild-type strain, the *cdc73Δ* single mutant strain, and the *cdc73Δ tel1Δ* and *cdc73Δ yku80Δ* double mutant strains (Fig 3; S3 and S4 Tables; S2–S8 Figs). In the wild-type sGCR strain, 46% of the GCRs analyzed (5 of 11) were produced by *de novo* telomere addition, 18% (2 of the 11) were produced by HR between the *SUP53* tRNA gene introduced by the *can1*::*P*_*LEU2*_*-NAT* marker and another leucine tRNA gene, and 36% (4 of the 11) were produced by HR between the *YCLWdelta5* fragment introduced by the *can1*::*P*_*LEU2*_*-NAT* marker and another Ty-related sequence (Fig 3A; S5,S9 and S10 Figs; S4 Table). The presence of both *de novo* telomere addition and HR-mediated GCRs among the GCRs selected in the sGCR assay is useful for characterizing mutations that alter the GCR spectra.

Analysis of GCRs formed in the *cdc73Δ*, *cdc73Δ tel1Δ*, and *cdc73Δ yku80Δ* sGCR strains revealed that no *de novo* telomere addition GCRs were recovered when *CDC73* was deleted (Fig 3A; S6–S8 Figs; S4 Table). Remarkably, the majority of the GCRs selected in strains containing a *cdc73Δ* mutation were inverted duplications, and most of these contained a second breakpoint that was mediated by HR (Fig 3G; S11 Fig). Inverted duplications mediated by hairpins were frequent in the *cdc73Δ tel1Δ* sGCR strain (Fig 3H; S12 Fig), which is consistent with the previously observed increase in hairpin-mediated inverted duplications observed in the uGCR assay for the *tel1Δ* single mutant strain [52]. For inverted duplication GCRs, the initial inversion GCRs would be predicted to be dicentric, but in all cases identified here, these GCRs underwent additional rearrangements to generate stable monocentric chromosomes. These additional rearrangements commonly involved HR between repetitive elements on chrV L and other repetitive elements elsewhere in the genome, including an unannotated delta sequence on chrV R (S13 and S14 Figs). All of the GCRs observed other than *de novo* telomere addition-mediated GCRs were different types of translocations; the rates of accumulating these translocations in the sGCR assay relative to the wild-type rate were increased 52-fold for the *cdc73Δ* single mutant, 242-fold for the *cdc73Δ tel1Δ* double mutant, and 460-fold for the *cdc73Δ yku80Δ* double mutant sGCR strains. Most GCR-containing strains contained a normal complement of chromosomes, except for one *cdc73Δ yku80Δ* GCR-containing strain that contained two copies of chrXVI (S15 Fig).

Taken together, the shift in the GCR spectra in sGCR strains lacking *CDC73* is consistent with an underlying defect in telomere homeostasis as most mutations that result in high GCR rates result in increased levels of *de novo* telomere addition GCRs as long as functional telomerase is present [55]. Given the limits on the numbers of GCRs we can presently sequence, our analysis cannot definitively prove that *de novo* telomere addition GCRs are not formed when *CDC73* is deleted, but does demonstrate that other types of GCRs, which are all different types of translocations, are selectively increased (e.g., the increase in the rate of *de novo* telomere additions in the *cdc73Δ* mutant relative to the wild-type is <5-fold compared to a 52-fold increase in the rate of translocations). The relative lack of *de novo* telomere addition GCRs among the GCRs selected in the sGCR assay in strains containing *cdc73Δ* mutations could indicate a complete failure of *de novo* telomere additions, as is observed with strains with deletions of *YKU80* or genes encoding telomerase subunits [55], or a partial defect that only decreases the efficiency of *de novo* telomere additions relative to other GCR-forming mechanisms, as is observed with *tel1Δ* strains [52,55]. We therefore combined the *cdc73Δ* mutation with a deletion of *PIF1*, which causes a substantial increase in GCRs formed through an increase in *de novo* telomere additions due to decreased inhibition of telomerase at DSBs [56,57], even under conditions where *pif1* mutations potentially prevent the formation of GCRs mediated by break-induced replication [52,55]. Mutations inhibiting *de novo* telomere addition suppress the increased GCR rate caused by the *pif1Δ* mutation, whereas mutations causing only reduced efficiency of *de novo* telomere addition do not [52,55]. The *cdc73Δ* mutation partially suppressed the increased GCR rate caused by the *pif1Δ* mutation (Table 1), suggesting that the *cdc73Δ* mutation causes a substantial, but incomplete, defect in the formation of GCRs mediated by *de novo* telomere addition. This could be due to reduced levels of functional telomerase resulting from the partial reduction of TLC1 telomerase RNA levels observed in *cdc73Δ* mutants [32].

### A *cdc73Δ* mutation synergizes with deletion of *YKU80* and *TEL1* to cause shortened telomeres

**Fig 4:**
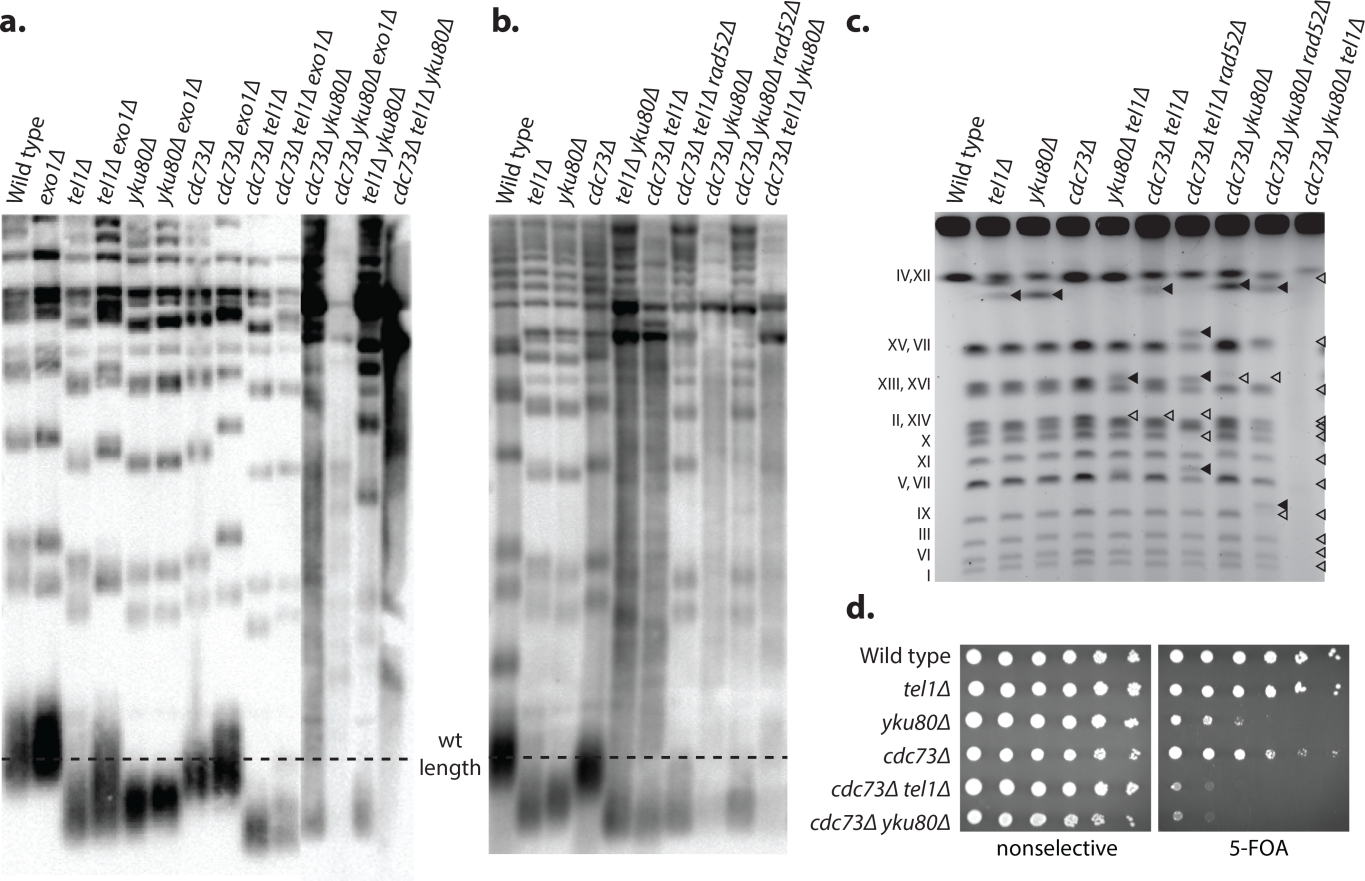
Loss of *CDC73* results in a telomere defect. **a.** Southern blot of *Xho*I-digested genomic DNA isolated from strains of the indicated genotypes derived by sporulation of appropriate diploids and analyzed with a telomere-specific probe immediately after sporulation and genotyping. The dashed line corresponds to wild-type telomere length. **b.** Strains were serially propagated on non-selective media for >20 restreaks and then tested by telomere Southern blot as above. **c.** Strains of the indicated genotypes were obtained by sporulation of heterozygous diploids and analyzed by pulse field gel electrophoresis. Wild-type chromosome sizes are labeled (left). Chromosome bands with new sizes are indicated with solid triangles, and missing bands are indicated with open triangles. Decreased band intensity and increased smearing can be seen in strains that were shown to undergo senescence. **d.** TPE was assayed by plating 10-fold serial dilutions of *cdc73Δ*, *tel1Δ*, and *yku80Δ* single and double mutant strains on selective media or selective media containing 5FOA. Loss of telomeric silencing is indicated by increased sensitivity to 5FOA.

Deletions of *CDC73*, *YKU70*, *YKU80* and *TEL1* all result in shortened telomeres [32,46,47]. To investigate if *cdc73Δ* double and triple mutant strains have increased telomere defects in addition to increased GCR rates, we generated haploid single, double, and triple mutant strains containing different combinations of *CDC73*, *EXO1*, *TEL1* and *YKU80* deletions by crossing mutant strains to each other to generate fresh mutant haploid spore clones for telomere length analysis. Consistent with previous results [32,46,47], telomere lengths were reduced in *cdc73Δ*, and to a greater extent in *tel1Δ*, and *yku80Δ* single mutant strains (Fig 4A). Exo1 plays a role in resection of deprotected telomeres [58] and deleting *EXO1* partially restored the shortened telomeres caused by the *cdc73Δ*, *tel1Δ*, and *yku80Δ* mutations; this is consistent with prior observations that *exo1Δ yku80Δ* double mutants have slightly longer telomeres than *yku80Δ* single mutants [59]. The *tel1Δ yku80Δ*, *cdc73Δ yku80Δ*, and *cdc73Δ tel1Δ* double mutant combinations all showed potential signs of additional telomere dysfunction compared to the respective single mutants, which included: (1) a telomere length that was shorter than seen in the respective single mutants (*cdc73Δ tel1Δ*) or potentially shorter than seen in the respective single mutants (*tel1Δ yku80Δ*, which was previously reported [60], and *cdc73Δ yku80Δ*); and (2) a smeared telomere pattern (*tel1Δ yku80Δ* and *cdc73Δ yku80Δ*), which was reminiscent of the telomere pattern seen in telomerase-defective post-senescent survivors that maintain their telomeres by alternative mechanisms [61]. Remarkably, the *cdc73Δ tel1Δ yku80Δ* triple mutant strain did not have a distinct telomere-containing band, but rather had only a smeared pattern, suggestive an even stronger telomere defect. The genetic interactions observed between the *cdc73Δ*, *tel1Δ*, and *yku80Δ* mutations resulting in increased telomere dysfunction mirrors the synergistic increases in GCR rates seen in strains containing combinations of these mutations.

### The slow growth of the *cdc73Δ* single and double mutants partially recovers after many rounds of serial restreaking

The *cdc73Δ* single mutant and the *tel1Δ yku80Δ*, *cdc73Δ yku80Δ*, and *cdc73Δ tel1Δ* double mutants all grow slowly and have evidence of telomere defects. We therefore investigated whether or not these strains would show evidence of crisis, escape from senescence and improved growth by serially restreaking the mutant strains on non-selective medium (S16 Fig). To ensure that our serial restreaking procedure could detect senescence and recovery, we tested the *tel1Δ yku80Δ* double mutant strain and found it initially grew slowly but eventually recovered a wild-type growth rate as previously reported [62] (not illustrated). In contrast, the slow growth of the *cdc73Δ* single mutant and the even slower growth of the *cdc73Δ yku80Δ*, and *cdc73Δ tel1Δ* double mutants showed only partial improvement in growth after 11 rounds of restreaking and never achieved wild-type growth rates. One possible explanation for this difference is that telomere maintenance-independent effects on transcription could also contribute to the slow growth phenotype caused by the *cdc73Δ* mutation.

The telomere structures of these serially propagated strains were analyzed by Southern blot and the telomere species of the *tel1Δ yku80Δ*, *cdc73Δ tel1Δ*, *cdc73Δ yku80Δ*, and *cdc73Δ tel1Δ yku80Δ* strains were all observed to contain smeared telomere fragments (Fig 4B); this suggests that the telomeres in these mutants may be partially maintained by one of the *RAD52*-dependent telomerase-independent telomere maintenance pathways [61,63]. Consistent with this, the *cdc73Δ tel1Δ rad52Δ* and *cdc73Δ yku80Δ rad52Δ* triple mutants all had very short telomeres, but lacked the smeared pattern seen in the Southern blots (Fig 4B). In contrast, we were unable to generate a *cdc73Δ tel1Δ yku80Δ rad52Δ* quadruple mutant by either PCR mediated gene disruption or by crossing different mutant strains to each other; this is consistent with a requirement of *RAD52*-dependent HR in the *cdc73Δ tel1Δ yku80Δ* triple mutant either for telomere maintenance or for the repair of some other type of spontaneous DNA damage in this triple mutant.

### Strains with increasing levels of telomere defects have increased levels of chromosome alterations

Pulse field gel electrophoresis (PFGE) was used to analyze chromosomes from *cdc73Δ* single, double and triple mutant strains for the presence of aberrant sized chromosomes (Fig 4C). The *cdc73Δ*, *tel1Δ*, and *yku80Δ* single mutant strains and the *cdc73Δ tel1Δ*, *cdc73Δ yku80Δ*, and *tel1Δ yku80Δ* double mutant strains had chromosomal banding patterns that were similar to that from the respective wild-type strain, although the double mutants showed more chromosomes with abnormal sizes despite being grown in the absence of any selection for chromosome rearrangements. The *cdc73Δ tel1Δ rad52Δ* and *cdc73Δ yku80Δ rad52Δ* triple mutants had increased numbers of chromosomes with abnormal sizes compared to the respective *cdc73Δ tel1Δ* and *cdc73Δ yku80Δ* double mutants. In contrast, no chromosome bands were visible when the *cdc73Δ tel1Δ yku80Δ* triple mutant was analyzed, which is consistent with reports that chromosomes from post-senescent survivors are unable to enter PFGE gels, likely because of the structure of the HR intermediates that act in telomere maintenance [61]. The aberrant chromosomes observed in this experiment were not studied further; however, the structures of GCRs selected in many of these mutant strains have been determined (Fig 3).

### Strains with increasing levels of telomere defects show increased TPE defects

We also investigated whether *cdc73Δ* single and double mutants with telomere defects had TPE defects. Consistent with previous results [64], we found that deletion of *YKU80* caused significant TPE defects relative to wild-type cells and hence a decreased ability to grow on plates containing 5FOA (Fig 4D). In contrast, the *cdc73Δ* and *tel1Δ* single mutant strains had modest but easily detectible or no sensitivity to 5FOA, respectively (Fig 4D, S1A Fig, S17A Fig). However, the *cdc73Δ yku80Δ* and *cdc73Δ tel1Δ* double mutant strains showed increased sensitivity to 5FOA, suggesting increased perturbation of the chromatin structure proximal to the telomeres, and hence loss of silencing in these double mutants. Consistent with a synergistic defect in TPE rather than an indirect effect due to induction of ribonucleotide reductase [42], growth on 5FOA-containing plates was not restored by addition of HU (S1B Fig, S17A Fig, S20B Fig).

### Not all mutations affecting telomere length homeostasis synergize with *cdc73Δ*

To test interactions between *cdc73Δ* and additional telomere homeostasis mutations, we measured the dGCR rates of strains containing a *cdc73Δ* mutation in combination with deletions of *SIR2*, *SIR3*, or *SIR4*, which cause defects in TPE, telomere chromatin structure and, at least in the case of *SIR3* and *SIR4* (*SIR2* does not appear to have been tested) also cause shortened telomeres [61,65], but were missing from our screen as these genes are required for mating [61,66]. The single *sir2Δ*, *sir3Δ* and *sir4Δ* mutant dGCR rates were increased 6 to 8-fold relative to the wild-type dGCR rate, and the double mutation combinations with the *cdc73Δ* mutation resulted in a synergistic increase in dGCR rates that were 41 to 190-fold higher than the wild-type dGCR rates (S2 Table).

In contrast, only 9 of the 36 mutations tested (including *sir3Δ* and *sir4Δ*) that were known to cause shortened telomeres [46–48,67] resulted in synergistic increases in dGCR rates when combined with *cdc73Δ* (S18 Fig). However, of the 27 mutations that did not interact, 3 mutations caused extremely high GCR rates and 1 mutation was in a Paf1 complex genes making it unlikely that interactions could be detected. Of the remaining 23 non-interacting mutations, many caused weak or inconsistent phenotypes (*lst7Δ* was reported to cause both long and short telomeres), 20 were identified in only one of two genetic screens performed suggestive of causing weak or inconsistent phenotypes and in most cases have not yet been demonstrated as causing a defect in a specific aspect of telomere homeostasis such as defects in TPE. Moreover, the *cdc73Δ* mutation also caused a strong synergistic increase in the dGCR rate when combined with a deletion of *EXO1* (Table 1). *EXO1* encodes a 5’ to 3’ exonuclease that acts in different DNA repair pathways and is the primary nuclease that resects deprotected telomeres [68–70]. Unlike the case of the *cdc73*Δ mutation, combining the *exo1Δ* mutation with either a *yku80Δ* or a *tel1Δ* mutation did not cause synergistic increases in the dGCR rate (S2 Table). Taken together, these data do not argue that the *cdc73Δ* mutation causes synergistically increased GCR rates in strain backgrounds that have short telomeres *per se*. Rather, the interaction of *cdc73Δ* with *tel1Δ* and *yku80Δ* may reflect an interaction between mutations that disrupt specific aspects of telomere structure including telomere chromatin structure [61,65], nuclear localization of telomerase [71,72], and/or telomerase recruitment to telomeres [73,74].

### Overexpression of TLC1 partially suppresses the genomic instability of *cdc73Δ* strains

The data described above are consistent with a role for *CDC73* in suppressing genome instability arising due to telomere dysfunction. This effect could be due to roles of *CDC73* in promoting TLC1 transcription [32] or causing defects in transcriptional elongation that give rise to recombinogenic RNA:DNA hybrids (R-loops) [75–78], particularly at the sites of long noncoding telomeric repeat containing RNA (TERRA) [79]. We measured the TLC1 levels in *cdc73Δ*, *tel1Δ*, and *yku80Δ* single and double mutant strains and found that the *yku80Δ* and *cdc73Δ* mutations caused a small and large decrease in TLC1 levels, respectively, as previously reported [32] and that the *cdc73Δ tel1Δ* and *cdc73Δ yku80Δ* double mutants had the same level of TLC1 as the *cdc73Δ* single mutant (S17B Fig). Introduction of a plasmid expressing TLC1 into strains in the uGCR assay caused a statistically significant ~4-fold decrease in the GCR rate of the *cdc73Δ tel1Δ* double mutant and caused a small, but not statistically significant, decrease in the GCR rate of the *cdc73Δ yku80Δ* double mutant (S5 Table). Consistent with the suppression results, the TLC1 expression plasmid caused 1) increased TLC1 levels in all strains tested, 2) increased the telomere lengths in the *cdc73Δ* single mutant and the *cdc73Δ tel1Δ* double mutant, and 3) potentially a small increase in telomere length in the *cdc73Δ yku80Δ* double mutant as evidenced by a modest increase in more slowly migrating telomere species (S19 Fig). We also measured the TERRA levels in *cdc73Δ*, *tel1Δ*, and *yku80Δ* single and double mutant strains and found that these mutants did not significantly affect TERRA accumulation, except for an increase of the chrXV L TERRA in a *yku80Δ* single mutant (S17C Fig). To test if the effects of *cdc73Δ* might be due to the accumulation of R-loops, we introduced a plasmid bearing *RNH1*, which encodes *S*. *cerevisiae* RNase H1, into uGCR assay strains. In contrast to TLC1 overexpression, the *RNH1* plasmid did not substantially affect the uGCR rate of either the *cdc73Δ tel1Δ* double mutant or the *cdc73Δ yku80Δ* double mutant (S5 Table). Taken together, these data suggest that the increased GCR rate caused by the *cdc73Δ* mutation may in part reflect an alteration in telomere structure caused by reduced telomerase activity due to reduced TLC1 levels. However, the synergistic increases in GCR rates seen in the *cdc73Δ tel1Δ* and *cdc73Δ yku80Δ* double mutants (and potentially the *ctr9Δ* and *paf1Δ* double mutants) is unlikely to be explained solely by reduced TLC1 levels as these double mutants have the same TLC1 levels as the *cdc73Δ* single mutant.

### An internal 105 amino acid region of Cdc73 is necessary and sufficient for Cdc73 function

**Fig 5:**
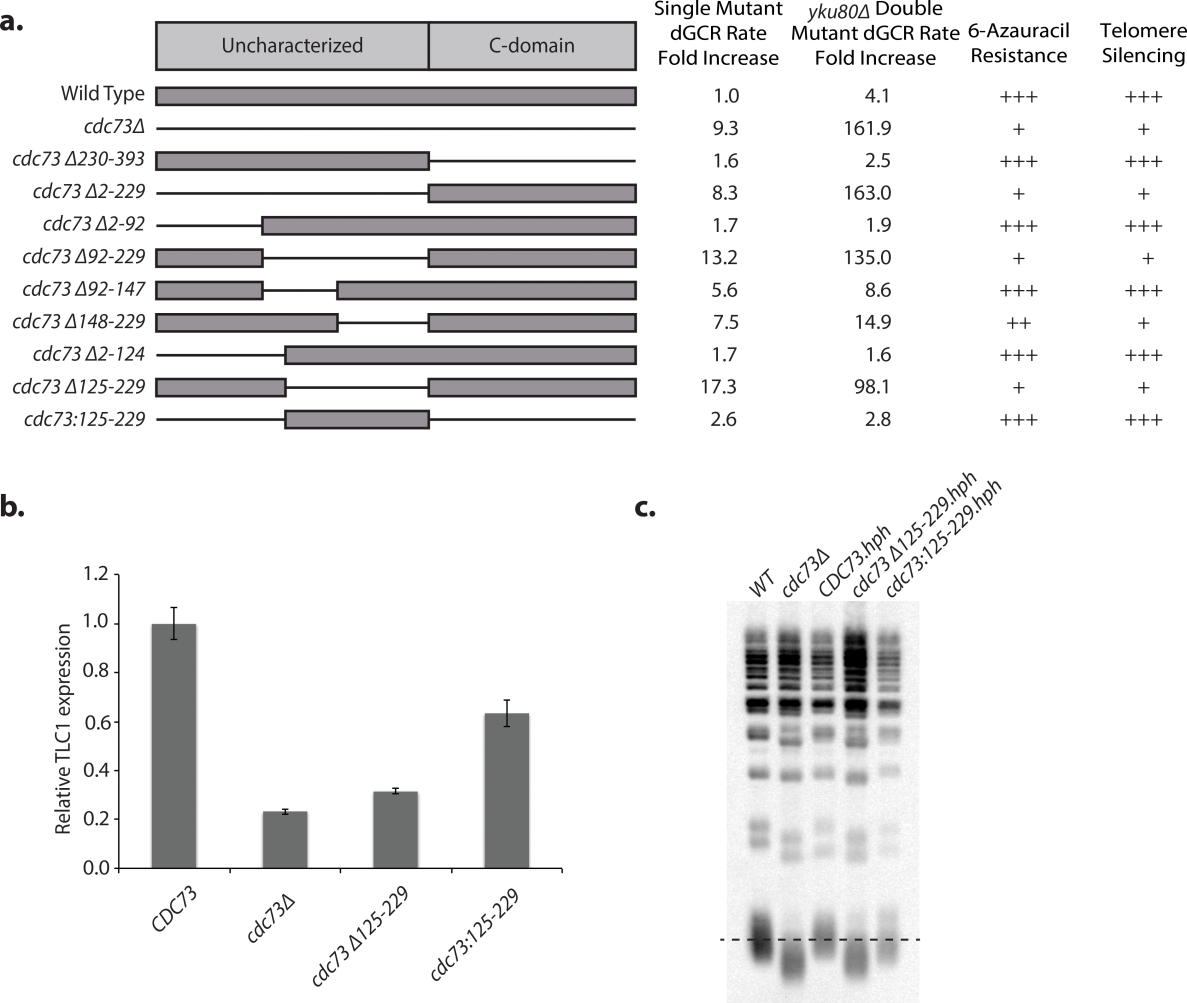
Cdc73 residues 125–229 are necessary and sufficient for its function. **a.** Various Cdc73 deletion constructs are shown; thin lines indicate deleted regions. Mutant constructs were tested for effects on GCR rates, transcription elongation as determined by resistance to 6-azauracil, and telomere silencing as determined by sensitivity to 5-FOA. “+++” corresponds to wild-type, “–” corresponds to a severe defect and “++” and “+” correspond to intermediate defects. **b.** Expression of TLC1 RNA was measured by RT-qPCR. Both deletion of *CDC73* and the *cdc73Δ125–229* allele result in substantial reduction of TLC1 expression, whereas *cdc73*:*125–229* allele results in partial restoration of TLC1 expression. **c.** Telomere Southern Blot. The dashed line indicates the wild-type telomere length.

Cdc73, like other members of the Paf1 complex, has no known enzymatic activity [24]. The N-terminal region (*S*. *cerevisiae* residues 1–229) lacks identifiable domains; whereas the C-terminal domain (*S*. *cerevisiae* residues 230–393) has a conserved GTPase-like fold [41,80] and has been proposed on the basis of chemical crosslinking and cryo-electron microscopy to make direct interactions with the RNA polymerase II subunit Rpb3 [81]. We replaced the wild-type chromosomal copy of *CDC73* with various *CDC73* deletion mutations to gain insights into Cdc73 function (Fig 5A; S20 Fig; S6 Table). We found that deletion of the C-terminal domain (*cdc73Δ230–39*3) resulted in wild-type dGCR rates, normal sensitivity to 6-azauracil and normal TPE. This result contrasts with a previous report suggesting that a *cdc73Δ231-393-TAP* construct causes increased sensitivity to 6-azauracil relative to wild-type *CDC73* [41]; this difference may be due to the presence of the TAP tag in the previous study. On the other hand, deletion of the N-terminal region (*cdc73Δ2–229*), caused defects in all three assays that were similar to those caused by the *cdc73Δ* single mutation.

Additional analysis of *CDC73* (Fig 5A) defined a minimal deletion, *cdc73Δ125–229*, that caused a similar fold-increase in the dGCR rate compared to that caused by the *cdc73Δ* single mutation (17.3-fold increase vs. 9.3-fold increase) and caused a synergistic increase in the dGCR rate when combined with the *yku80Δ* mutation that was similar to that observed with the *cdc73Δ* mutation (98.1-fold increase vs. 162-fold increase). This minimal deletion also caused increased sensitivity to 6-azauracil and reduced TPE (Fig 5A, S20A Fig) as well as reduced TLC1 levels (Fig 5B) and shorter telomeres (Fig 5C) similar to that caused by the *cdc73Δ* single mutation; as before, addition of sublethal concentrations of HU to distinguish TPE from 5FOA-induced overexpression of ribonucleotide reductase verified that the *cdc73Δ125–229* mutation, like the *cdc73*Δ mutation, caused TPE defects (S20B Fig). As the effect of the *cdc73Δ125–229* mutation could either have been due to loss of a functional region of Cdc73 or due to causing defects in folding Cdc73, we generated a gene construct that encoded only residues 125–229 (*cdc73*:*125–229*). This gene construct, which encoded 105 residues from the center of Cdc73, was sufficient to substantially restore Cdc73 functions in suppressing GCRs, maintenance of TLC1 levels, TPE, and telomere length homeostasis (Fig 5, S20 Fig). These results define a minimal functional Cdc73 construct, Cdc73:125–229, and a minimal non-functional Cdc73 construct, Cdc73Δ125–229.

Residues 125–229 of Cdc73 precede the C-terminal GTPase domain and lie in a region that is predicted to be less ordered by IUPRED [82] (S21A Fig) and that has reduced conservation (S21B Fig). Previous chemical crosslinking of the Paf1 complex bound to RNA polymerase II identified 22 crosslinks between Cdc73 and other Paf1 subunits of which 19 were between Cdc73 and Ctr9, which is primarily composed of tetratricopetide repeat (TPR) domains [81]. Analysis of these data also revealed that the Cdc73 region containing residues 125–229 had 9 crosslinks to Ctr9 (~50% of Cdc73-Ctr9 crosslinks), 2 crosslinks to Leo1, 2 crosslinks to Rpb11, and 1 crosslink to Rpb2 (S21C Fig). Together these data are consistent with the possibility that the TPR domains of Ctr9 bind to an unstructured Cdc73 peptide or Cdc73 alpha helices, rather than a folded Cdc73 domain, like other known TPR-peptide interactions [83]. To test for a direct Cdc73-Ctr9 interaction in the Paf1 complex, we tested the ability of Paf1 and Cdc73 to co-immunoprecipitate in a wild-type strain or strains with deletions of *LEO1*, *RTF1*, or *CTR9* (S21D Fig). Consistent with this hypothesis, the Paf1-Cdc73 interaction was lost in the *ctr9Δ* strain, whereas deletions of *LEO1* and *RTF1* had only modest effects on the Paf1-Cdc73 interaction.

### Functional and non-functional Cdc73 truncations can be distinguished by their ability to associate with the Paf1 complex

**Fig 6:**
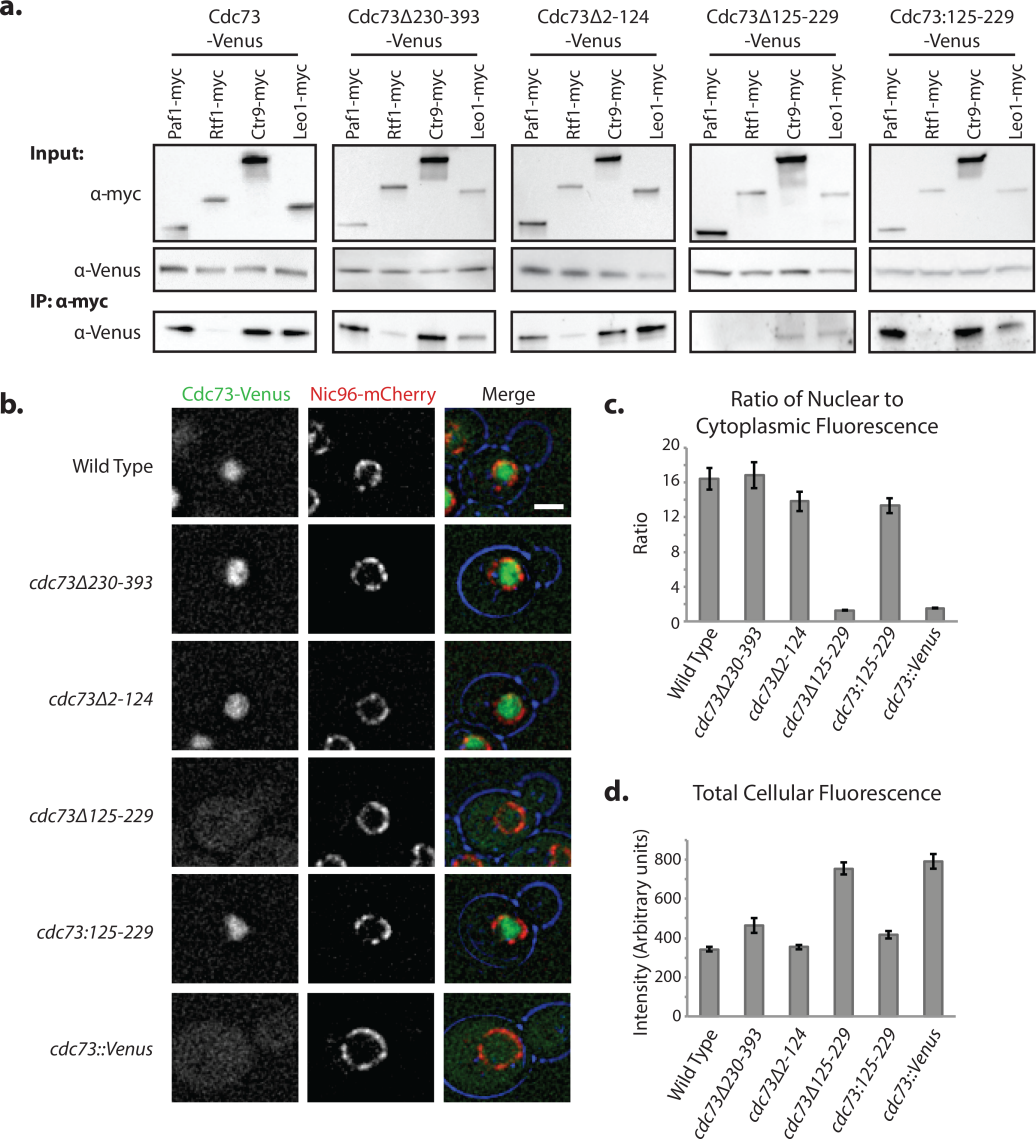
Functional Cdc73 variants associate with the Paf1 complex and localize to the nucleus. **a.** Functional Cdc73 variants can associate with the Paf1 complex. Wild-type and the indicated mutant Cdc73 proteins were tagged with a C-terminal Venus and other Paf1 subunits were individually tagged with C-terminal myc tags. Whole cell lysates were immunoprecipitated with anti-myc antibody and analyzed by Western blotting for coimmunoprecipitation using an anti-Venus antibody. **b-d.** Functional Cdc73 variants specifically localize to the nucleus. **b.** Wild-type and the indicated Cdc73 variants were tagged with C-terminal Venus and Nic96 (a member of the nuclear pore complex) was tagged with mCherry, and the cells were imaged by deconvolution microscopy. The *cdc73*::*Venus* control replaces the *CDC73* ORF with the sequence encoding the Venus protein. Scale bar is 2 μm. **c, d.** The ratio of nuclear to cytoplasmic fluorescence and total cellular fluorescence for each of the Cdc73 constructs was measured using ImageJ. The data represent averages of at least 20 cells; the error bars are the SEM.

As the *paf1Δ* mutation causes increased dGCR rates similar to those caused by the *cdc73Δ* mutation, we sought to determine if the functional truncated Cdc73 proteins bound the Paf1 complex and if the defects associated with the minimal non-functional Cdc73Δ125–229 truncation were due to loss of Paf1 complex association or due to defects in other functions. We tested the ability of C-terminally Venus-tagged full-length Cdc73, Cdc73Δ230–393, Cdc73Δ2–124, Cdc73Δ125–229, or Cdc73:125–229 to co-immunopreciptate with C-terminally myc-tagged Paf1, Rtf1, Ctr9, or Leo1; all tagged proteins were expressed from the respective chromosomal loci. Cell lysates from doubly tagged strains were prepared from log-phase cells and immunoprecipitated with anti-myc antibodies, and then probed by Western blotting using anti-GFP antibodies. Full-length Cdc73 co-immunoprecipitated with Paf1, Rtf1, Ctr9, and Leo1 (Fig 6A), although the interaction with Rtf1 appeared to be weaker than the interaction with the other Paf1 complex subunits, consistent with previous observations [22,43,84]. The functional Cdc73 truncations, Cdc73Δ230–393, Cdc73Δ2–124, and Cdc73:125–229, all associated with Paf1, Ctr9, Leo1, and Rtf1 (Fig 6A). Reduced binding to Leo1 was observed with both the Cdc73Δ230–393 and Cdc73:125–229 truncations, suggesting that the C-terminus of Cdc73 may stabilize Leo1 in the complex. In contrast, the non-functional Cdc73 truncation, Cdc73Δ125–229, had substantially reduced binding to each of the other Paf1 complex subunits; a low level of residual binding was only detected with Ctr9 and Leo1. Thus residues 125–229 of Cdc73 appear to be necessary and sufficient for stable binding of Cdc73 to the Paf1 complex.

### Functional Cdc73 proteins are localized to the nucleus

The Paf1 complex has been localized to the nucleus in wild-type cells by immunofluorescence [85], so we monitored the cellular localization of Cdc73 truncations. The wild-type and truncated forms of Cdc73 were C-terminally tagged with Venus, and functional versions of Cdc73, including the minimal construct Cdc73:125–229, localized to the nucleus (Fig 6B), with a high ratio of nuclear to cytoplasmic fluorescence (Fig 6C). In contrast, the non-functional Cdc73 truncation Cdc73Δ125–229, which did not stably associate with the Paf1 complex, had diffuse localization in both the nucleus and the cytoplasm, but was still expressed at normal levels based on total cellular fluorescence (Fig 6D). Thus, residues 125–229 of Cdc73 either include a nuclear localization signal or are necessary for binding to a Paf1 complex that is imported into the nucleus. Single mutant strains with deletions of *PAF1*, *CTR9*, *RTF1*, and *LEO1* appeared to have normal nuclear localization of a Cdc73-Venus fusion protein (S22A Fig), although these mutations resulted in enlarged cells and abnormally elongated buds, as previously described [18,86]. Similarly, deletion of *CDC73* did not prevent the nuclear localization of C-terminally Venus tagged Paf1, Rtf1, Ctr9, or Leo1 (S22B Fig), indicating that defects caused by the *cdc73Δ* mutation were not due to defects in the nuclear localization of other Paf1 complex subunits. Finally, the *cdc73Δ* mutation did not cause major changes in the cellular levels of the other Paf1 complex subunits as measured by Western blot (S22C Fig). These localization data are consistent with the observation that all Paf1 complex subunits other than Cdc73 are predicted to contain nuclear localization signals (S23 Fig); this is different from that seen with human Cdc73, which possesses a functional N-terminal nuclear localization signal [87]. Together, these data suggest that Cdc73 does not regulate the cellular localization of the Paf1 complex, but instead mediates the suppression of genome instability once the complex is already in the nucleus, potentially through contributions to overall complex stability or conformation.

## Discussion

Transcription, and defects in transcription including those that lead to the accumulation of R-loops, are becoming an increasingly well-appreciated source of genome instability [10,11]. Using a screen to identify genes that suppress the accumulation of GCRs, we found the loss of *CDC73* results in increased rates of accumulating GCRs in three different GCR assays. We also found that a *cdc73Δ* mutation resulted in synergistic increases in GCR rates and in increased levels of telomere dysfunction when combined with either *tel1Δ* or *yku80Δ* mutations. This is reminiscent of the observation that *tlc1Δ tel1Δ* double mutants have synergistic increases in GCR rates relative to the respective single mutants, although they show delayed senescence and delayed loss of telomeres [88]; analysis of GCR rates and other telomere-related phenotypes in *tlc1Δ yku80Δ* double mutants was not possible as these double mutants cannot be propagated [59,89]. The fact that the *cdc73Δ tel1Δ yku80Δ* triple mutant appears to be highly defective in telomerase function and also shows a large synergistic increase in the rate of accumulating GCRs further suggests that telomere dysfunction is likely a hallmark of genome instability in *cdc73Δ* strains and that *cdc73Δ*, *tel1Δ*, and *yku80Δ* mutations all cause different defects that contribute to increased rates of accumulation of GCRs. A role for *CDC73* in contributing to telomerase function is also consistent with our inability to observe GCRs formed by *de novo* telomere additions relative to the large increase in the levels of different translocation GCRs among the GCRs selected in the sGCR assay in *cdc73Δ* mutants. Consistent with the observation that *cdc73* defects result in reduced levels of the TLC1 RNA component of telomerase [32], overexpression of TLC1 partially suppressed the increased GCR rate of the *cdc73Δ tel1Δ* double mutant. In contrast, over-expression of RNase H1, which degrades R-loops, did not suppress the increased GCR rate of the *cdc73Δ tel1Δ* double mutant.

**Fig 7:**
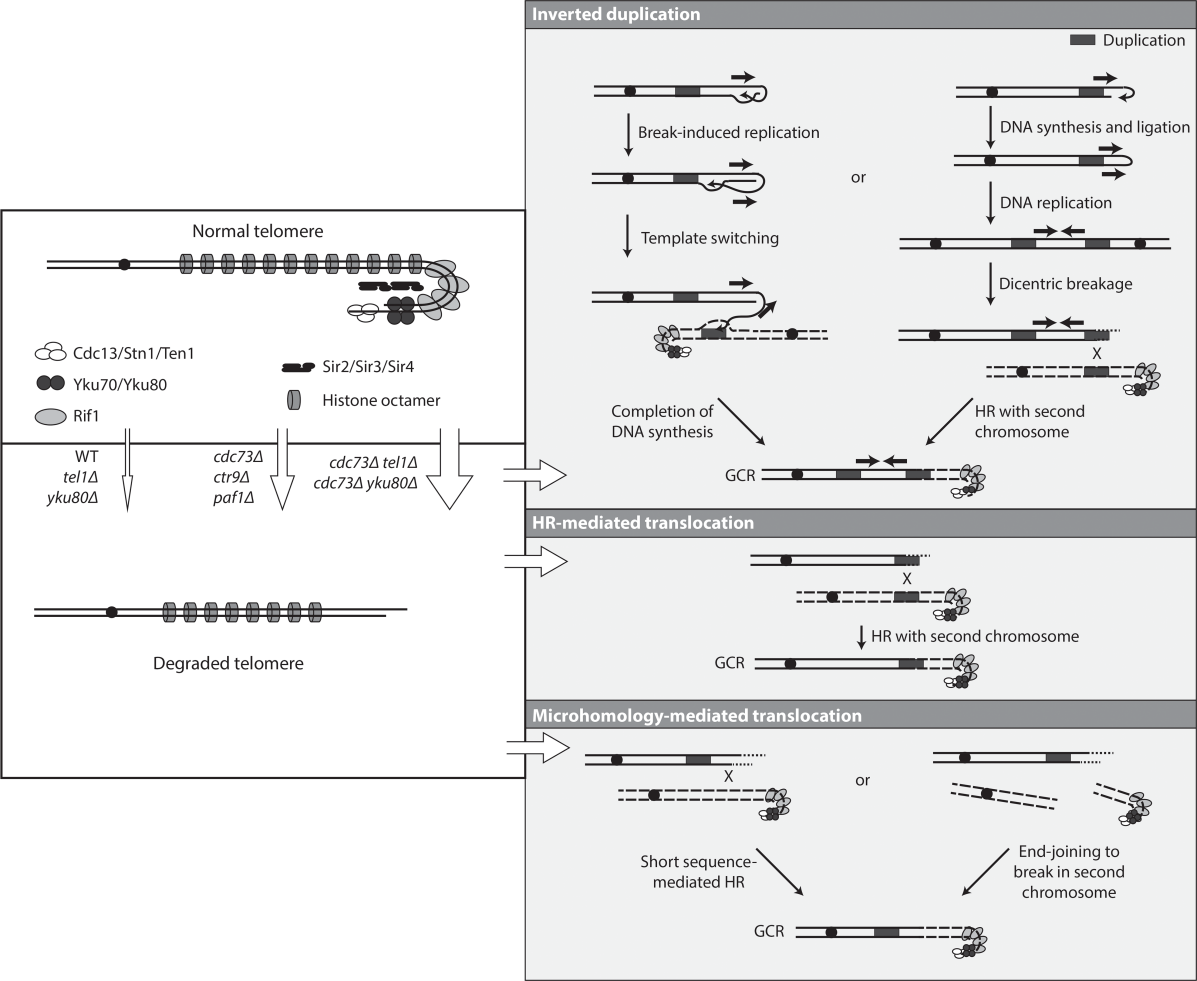
Formation of GCRs in strains lacking *CDC73* and strains lacking both *CDC73* and *TEL1* or *YKU80*. Deletion of *CDC73* (and likely *CTR9* and *PAF1*) gives rise to an increased frequency of dysfunctional telomeres that are subject to degradation. This frequency of these dysfunctional telomeres increases when *TEL1* or *YKU80* is additionally deleted. The degraded telomere is then processed to form inverted duplication GCRs, HR-mediated translocation GCRs, or microhomology-mediated translocation GCRs. The observed inverted duplications can be generated by invasion of the exposed 3’ end into sequences on the same chromosome followed by Break-Induced Replication until copying reaches the end of the chromosome or until it reaches a duplication that allows template switching and duplication of a second chromosome. The same products can also be generated through the formation of a dicentric chromosome generated via replication of a capped chromosome; breakage of the dicentric chromosome gives rise to at least one additional round of HR-mediated rearrangements involving duplications on the broken chromosome and a second, potentially intact, chromosome. HR-mediated translocation GCRs can be formed when the dysfunctional telomere is degraded to the position of a duplication, which then can mediate HR with a second chromosome. Microhomology-mediated translocations can be formed when degradation reaches a short sequence capable of mediating HR with a target or when end joining occurs to a second broken chromosome; the junctions with microhomologies likely involve base pairing between exposed single-stranded DNAs in both broken chromosomes.

The absence of telomerase in *S*. *cerevisiae* results in shortening of telomeres and reduced rates of cell growth until telomerase negative cells undergo crisis and survivors emerge in which telomeres are maintained by one of two different HR-mediated telomere maintenance pathways [61,63]. These surviving cells do not have increased rates of accumulating GCRs, although additional genetic defects can result in synergistic increases in GCR rates in these telomerase negative cells [55]. One exception where telomerase defects alone result in increased GCRs is telomerase negative cells that have been stabilized by re-expression of telomerase during crisis before the shortened telomeres have started to be maintained by HR [90]. In addition, *tel1Δ* mutations, which by themselves result in shortened telomeres and small increases in GCR rates, can result in large increases in GCR rates when combined with *mec1* or other mutations [91]. Under all of these conditions, the telomeres with altered structures fuse to the ends of broken chromosomes, and the resulting fusion chromosomes then appear to undergo breakage and additional rearrangement events [92]; these altered telomeres can also undergo telomere to telomere fusion [93]. The structural analysis presented here showed that the *cdc73Δ* mutation that causes an increased GCR rate and the *cdc73Δ tel1Δ* and *cdc73Δ yku80Δ* double mutation combinations that cause synergistic increases in GCR rates did not appear to cause the accumulation of either *de novo* telomere addition-mediated GCRs or GCRs mediated by fusion of altered telomeres to broken chromosome ends. Telomerase activity is likely reduced but not absent in *cdc73Δ* mutants [32], which would explain the presence of telomeres that are shorter than normal and this is likely sufficient to result in modest increases in the rate of accumulating GCRs as well as the absence of *de novo* telomere addition-mediated GCRs. When a *cdc73Δ* mutation is combined with other mutations like *tel1Δ* and *yku80Δ*, which affect telomere maintenance in different ways and also cause shortened telomeres, there is an increased defect in telomere maintenance and an increased alteration of telomere chromatin structure as indicated by synergistic increases in TPE defects and a synergistic increase in the rate of accumulating GCRs. A hypothesis that explains the increased GCR rates and the spectrum of GCRs observed is that in these mutants reduced telomere maintenance combined with alterations in telomere chromatin structure results in a fraction of chromosome ends that escape protection and undergo extensive degradation (Fig 7). These degraded chromosome ends can then be processed by end joining to other DSBs, hairpin formation or short sequence-mediated HR resulting in the GCRs selected in the sGCR assay or longer sequence non-allelic HR resulting in the translocations selected in the dGCR assay. This mechanism also accounts for the increased GCR rates seen in *paf1Δ* and *ctr9Δ* single and double mutants analyzed as these mutations also cause telomere maintenance and telomere chromatin structure defects as evidenced by reduced *TLC1* levels, short telomeres and TPE defects [32]. The lack of or limited increased GCR rates seen in *rtf1Δ* and *leo1Δ* single and double mutants is also accounted for by this mechanism as these latter mutations have smaller effects on TLC1 levels and telomere shortening [32], and in the case of *leo1Δ* mutations, no defect in TPE reflective of alterations in telomere chromatin structure.

The Paf1 complex promotes transcription elongation, 3’-end mRNA maturation, and histone modification [16,21–24]. Our results demonstrate that different subunits of the Paf1 complex subunits promote different Paf1 complex functions: (1) suppression of GCRs primarily requires Paf1, Ctr9, and Cdc73; (2) resistance to 6-azauracil inhibition of transcriptional elongation primarily requires Paf1 and Ctr9; and (3) silencing of telomere-proximal genes requires Cdc73, Paf1, Ctr9 and Rtf1 to differing degrees. The rather disparate effects of deleting genes encoding different Paf1 complex subunits observed here mirrors previous observations of different requirements for individual Paf1 complex subunits under different stress conditions [37]. Using an assay that detected chromosome loss and GCRs but did not distinguish between the two, a previous study showed that deletions of *CDC73* and *LEO1*, but not *PAF1*, resulted in increased genome instability that could be suppressed by increased expression of RNase H1 [12]. The relatively important role of Paf1 in all of the Paf1 complex functions (our results as well as in previous studies [37]) is consistent with the idea that Paf1 functions by mediating recruitment of the other Paf1 subunits to the different processes they functions in. Alternatively, Paf1 may provide the major function of the Paf1 complex and may be recruited to different processes by different Paf1 complex subunits: Leo1 and Rtf1 bind RNA [94]; Rtf1 binds phosphorylated Spt5, which is a component of TFIIS and binds the elongating RNA polymerase II complex [95,96]; and the Cdc73 C-terminal domain mediates binding to the phosphorylated C-terminal domain (CTD) of RNA polymerase II [97].

The importance of the interaction of Cdc73 with Paf1 is demonstrated by the deletion analysis of Cdc73. The C-terminal domain and the N-terminal regions of Cdc73 were found to be dispensable for *CDC73* function. However, the central 105 amino acid region (residues 125–229) was necessary and sufficient to: (1) suppress the defects of *cdc73Δ* strains studied here; (2) mediate incorporation into the Paf1 complex; and (3) promote nuclear localization of Cdc73. Remarkably, the C-terminal region, which binds the phosphorylated RNA polymerase II CTD [97] and contributes to suppression of Ty element expression [41], was not required for any of the functions analyzed here. The dispensable nature of the Cdc73 C-terminal domain could be consistent with the redundancy of recruitment of the Paf1 complex to RNA polymerase II by either Cdc73 binding to the phosphorylated RNA polymerase II CTD or by Rtf1 binding to phosphorylated Spt5 [97]. This redundancy also explains the synergistic defect in 6-azauracil sensitivity caused by combining a deletion of the C-terminal domain of Cdc73 with loss of Rtf1 [41]. Moreover, the available data suggest that the central 105 amino acid region (residues 125–229) of Cdc73 plays some previously unappreciated function in the Paf1 complex. Extensive chemical crosslinking between this region of Cdc73 and the TPR domain containing protein Ctr9 [81] and the requirement for Ctr9 for coimmunoprecipitation of Cdc73 with Paf1 suggest that the Ctr9-Cdc73 interaction recruits Cdc73 to the Paf1 complex. The fact we were unable to computationally predict a function-associated motif or domain structure within the central 105 amino acid region and that the N-terminus of *S*. *cerevisiae* Cdc73 up to residue 236 is highly sensitive to partial proteolysis [41] suggests the central 105 amino acid region of Cdc73 is likely unstructured in the absence of the Paf1 complex. This is consistent with the role of TPR domains in binding alpha-helices and unstructured peptides [83]. Together these data are also consistent with the fact that *ctr9Δ* mutations, like *cdc73Δ* mutations, also cause increased GCR rates, cause synergistic increases in GCR rates when combined with *yku80Δ* and *tel1Δ* mutations, have TPE defects, and defects in TLC1 expression [32].

Mutations in human *CDC73* (also called *HRPT2*) identified in cases of sporadic and hereditary parathyroid carcinomas appear to primarily be loss-of-function mutations including frameshifts, premature stop codons, and deletions that result in truncated proteins. In many cases, the heterozygous germline mutations observed are associated with events leading to loss-of-heterozygosity in tumors; however, some tumors appear to have amplification of the mutant copy of *CDC73*, suggesting a dominant genetic phenotype [28–30,98–100]. The region of human Cdc73 (also called parafibromin) responsible for Paf1 complex binding [29] is in a region that is similar to the central 105 amino acid region in *S*. *cerevisiae* Cdc73 identified here, and at least some mutant versions of human Cdc73 seen in parathyroid carcinomas have lost their ability to interact with the Paf1 complex [101]. The 3 mutations in *CTR9* found in Wilms tumor families comprised a nonsense mutation and 2 splice site mutations, all of which were consistent with causing loss-of-function [31]. Our results suggest that the *CDC73* mutations seen in sporadic and hereditary parathyroid carcinomas and the *CTR9* mutations found in Wilms tumor could cause increased genome instability; however, it is not currently known if these defects in human *CDC73* and *CTR9* cause genome instability and telomere dysfunction in human cells as observed here for the *S*. *cerevisiae cdc73Δ* mutation. Given the ability of the Paf1 complex to affect transcriptional elongation, RNA 5’ end maturation, and histone modification, inherited and sporadic *CDC73* mutations and inherited *CTR9* mutations in human cancers could have pleiotropic effects in which increased genome instability might not play the only role in carcinogenesis.

## Materials and methods

### Construction and propagation of strains and plasmids

All *S*. *cerevisiae* strains used in this study were derived from S288c and were constructed by standard PCR-based gene disruption methods or by mating to strains containing mutations of interest (S7 Table; [102,103]). GCR assays were performed using derivatives of RDKY7635 (dGCR assay), RDKY7964 (sGCR assay), and RDKY6677, (uGCR assay) (S7 Table; [6,51]). The Venus, mCherry and 9myc tags were amplified from pBS7, pBS35, and pYM19, respectively [102,104], inserted at the 3’ end of the indicated genes using standard methods. For determining GCR rates of strains transformed with the RNase H1 tet-off overexpression plasmid pCM184RNH1 (a gift from Andrés Aguilera, Universidad de Sevilla, Seville, Spain [105]) or the *ADH1* promoter *TLC1* overexpression plasmid pVL2679 (a gift from Victoria Lundblad, Salk Institute), transformants were cultured overnight in complete synthetic medium (CSM)–Trp liquid media and plated onto either CSM–Trp medium or CSM–Arg–Trp medium supplemented with 1 g/L 5FOA and 60 mg/L canavanine. To test for transcription elongation defects, 6-azauracil (Sigma-Aldrich) was added to synthetic complete medium at a final concentration of 50 μg/ml.

*CDC73*, including 998 bp upstream and 536 bp downstream, was amplified by PCR using the primers 5’-CAC CGA ATT GCA AGC GCT TGC AAC TTG TTC TTT CTG TGC -3’ and 5’-GAA TTG CAA GCG CTC CCA TGG AAA TGA GAG AAG C-3’ (*Afe*I cut site underlined) and cloned into the pENTR/D-TOPO vector (Thermo Fisher Scientific) to generate pRDK1705. The hygromycin B resistance gene was amplified from the plasmid pFA6a-hphNT1 with the primers 5’-GAA TTG CAA AGC TTC GGA TCC CCG GGT TAA TTA A-3’ and 5’-GAA TTG CAA AGC TTT AGG GAG ACC GGC AGA TCC G-3’ (*Hind*III cut site underlined) and inserted into pRDK1705 at a *Hin*dIII cut site located 693 bp upstream of the *CDC73* start codon to make plasmid pRDK1706. The *cdc73* alleles were made in pRDK1706 using the GeneArt Site-Directed Mutagenesis kit (Life Technologies) to generate pRDK1708 (*cdc73Δ230–393*), pRDK1770 (*cdc73Δ2–229*), pRDK1771 (*cdc73Δ92–229*), pRDK1772 (*cdc73Δ2–91*), pRDK1781 (*cdc73Δ92–147*), pRDK1782 (*cdc73Δ148–229*), pRDK1784 (*cdc73*:*92–229*), pRDK1788 (*cdc73Δ2–124*), pRDK1789 (*cdc73Δ125–229*), and pRDK1790 (*cdc73*:*125–229*). These plasmids were integrated at the endogenous *CDC73* locus by transformation with *Afe*I-digested plasmid DNA. Integrants were confirmed by PCR and Sanger sequencing.

### Systematic double mutant generation

We crossed a strain containing the dGCR assay and a *cdc73Δ* or *rtf1Δ* mutation against 638 strains from the *S*. *cerevisiae* deletion collection and obtained haploid progeny by germinating spores generated from the resulting diploids, as previously described [6].

### DNA content measurement by flow cytometry

Systematically generated *cdc73Δ* double mutants and control haploid and diploid strains were screened by flow cytometry for DNA content to exclude diploid isolates. Briefly, 10 μL aliquots of overnight cultures grown in YPD were added to 190 μL of fresh YPD, and the cells were incubated in a 30°C shaker for 3 hours. Cells were washed, resuspended in 60 μL of dH_2_O, and fixed with 140 μL of cold absolute ethanol. Fixed cells were sonicated and resuspended in 150 μL of 50 mM sodium citrate with 1 mg/mL Proteinase K (Sigma-Aldrich) and 0.25 mg/mL RNase A (Sigma-Aldrich) and incubated at 37°C overnight. Treated cells were washed, resuspended in 100 μL of 50 mM sodium citrate containing 1 μM Sytox Green (Life Technologies), and analyzed using a BDS LSR II flow cytometer at The Scripps Research Institute flow cytometry core facility. Data were analyzed using FlowJo v10 [106].

### Determination of GCR patch scores and rates

Patch tests for identifying systematically generated double mutants with increased GCR rates were performed as described [6]. GCR rates were determined using at least 14 independent cultures from 2 independent biological isolates of each strain using the fluctuation method as previously described [107]. Significantly different GCR rates were identified through analysis of the 95% confidence intervals.

### Analysis of dGCR structures

The t(V;XIV) and t(V;IV or X) homology-mediated translocation GCRs were identified by PCR, as previously described [51].

### Whole genome sequencing

Multiplexed paired-end libraries were constructed from 5 μg of genomic DNA purified using the Purgene kit (Qiagen). The genomic DNA was sheared by sonication and end-repaired using the End-it DNA End-repair kit (Epicentre Technologies). Common adaptors from the Multiplexing Sample Preparation Oligo Kit (Illumina) were then ligated to the genomic DNA fragments, and the fragments were then subjected to 18 cycles of amplification using the Library Amplification Readymix (KAPA Biosystems). The amplified products were fractionated on an agarose gel to select 600 bp fragments, which were subsequently sequenced on an Illumina HiSeq 2000 using the Illumina GAII sequencing procedure for paired-end short read sequencing. Reads from each read pair were mapped separately by bowtie version 2.2.1 [108] to a reference sequence that contained revision 64 of the *S*. *cerevisiae* S288c genome [109], *hisG* from *Samonella enterica*, and the *kanMX4* marker (S3 Table). Reads are available from National Center for Biotechnology Information Sequence Read Archive under accession number: SRP107803.

### Analysis of sGCR structures from sequencing data

GCR structures were determined using mapped reads using version 0.6 of the Pyrus suite (http://www.sourceforge.net/p/pyrus-seq) [52]. Rearrangements relative to the reference S288c genome were identified by analyzing the read depth distributions (S5–S8 Figs), the discordantly mapping read pairs (S2–S4 Figs; S4 Table), and/or extracting the sequences of the novel junctions (S9–S13 Figs). Associated junction-sequencing reads, which were reads that did not map to the reference but were in read pairs in which one end was adjacent to discordant reads defining a junction, were used to sequence novel junctions. Most hairpin-generated junctions (S12 Fig) could be determined using alignments of junction-sequencing reads. For junctions formed by HR between short repetitive elements (S9–S11 Figs) and for problematic hairpin-generated junctions (S12 Fig), the junction sequence could be derived by alignment of all reads in read pairs where one read was present in an “anchor” region adjacent to the junction of interest and the other read fell within the junction to be sequenced.

Junctions indicated by copy number changes, discordant read pairs, and junction sequencing were identified with a high degree of confidence; however, previous analyses have indicated that even junctions inferred from only copy number changes can be experimentally verified at high frequency [52,92,110,111]. Analysis of the sequencing data identified all of the genetic modifications introduced during construction of the starting strains, such as the *his3Δ200* deletion, (S2–S4 Figs) as well as the molecular features associated with the selected GCRs (S5–S13 Figs; S4 Table). Several inverted duplications (isolates 307, 324, and 331) with a *YCLWdelta5/YELWdelta1* junction copied very little sequence in the vicinity of *YELWdelta1*, and had an additional HR-mediated translocation between *YELWdelta1* and an unannotated delta sequence on chrV R, which we term here “*YERWdelta27*” (S14 Fig).

### Telomere Southern blotting

Telomere Southern blots were performed using a modified version of a previously described protocol [112]. Genomic DNA was purified from 50 mL overnight cultures using the Purgene kit (Qiagen). 5 μg of DNA was digested with *Xho*I (New England Biolabs) in a 50 μL reaction for 2 hr at 37°C. The reaction was stopped by adding 8 μL of loading buffer, and the samples were run on a 0.8% agarose gel in 0.5X TBE for 16 hr at 50 V. The DNA in the gel was transferred to Amersham Hybond-XL membranes (GE) by neutral capillary blotting, allowed to run overnight. The DNA was crosslinked to the membrane by UV irradiation in a Stratalinker (Stratagene) apparatus at maximum output for 60 seconds. Biotinylated TG probes were purchased from ValueGene. Probe hybridization was performed with ULTAhyb oligo hybridization buffer (Life Technologies) at 42°C for 1 hr. The membrane was then washed extensively and developed with a chemiluminescent nucleic acid detection kit (Life Technologies) and imaged with a Bio-Rad Imager.

### Pulse Field Gel Electrophoresis (PFGE)

DNA plugs for PFGE were prepared as described [113]. Strains were grown to saturation in 50 mL of YPD at 30°C for 3 days. Cell counts were measured by optical density at 600 nm, and 7.5 x 10^8^ cells from each strain were washed and resuspended in 200 μL of 50 mM EDTA, then mixed with 70 μL of 1 M sorbitol, 1 mM EDTA, 100 mM sodium citrate, 0.5% β-mercaptoethanol, 8 U/mL of zymolase. The cells were then mixed with 330 μL of liquefied 1% ultrapure agarose (Bio-Rad) to prepare multiple 80 μL plugs. The plugs were incubated in 15 mL conical tubes in 750 μL of 10 mM Tris pH 7.5, 500 mM EDTA pH 8, 1% β-mercaptoethanol for 16 hr at 37°C. The plugs were then incubated in 750 μL 10 mM Tris pH 7.5, 500 mM EDTA pH 8, 1% sodium N-lauryl sarcosine, 0.2% sodium dodecyl sulfate containing 2 mg/ml Proteinase K (Sigma-Aldrich) for 6 hr at 65°C. Finally, the plugs were washed in 50 mM EDTA pH 8 prior to resolving the chromosomes in a 1% agarose gel run in a CHEF (clamped homogeneous electric field electrophoresis) apparatus in chilled (14°C) 0.5x TBE (89 mM Tris-borate, pH 8.3, 25 mM EDTA). Electrophoresis was performed using a Bio-Rad CHEF-DRII apparatus at 6 V/cm, with a 60 to 120 s switch time for 24 h. The gels were stained with ethidium bromide and imaged.

### Telomere Position Effect (TPE) assay

The TPE assay was constructed by transforming BY4742 (*MATalpha leu2Δ0 his3Δ1 ura3Δ0 lys2Δ0*) with pADH4UCA ([38], a gift from Virginia Zakian, Princeton University) digested with *Sal*I and *Eco*RI. Integration of *URA3* into *ADH4*, which was verified by PCR, generated the strain RDKY8230, and mutant derivatives were constructed by PCR-mediated gene disruption (S7 Table). TPE was assayed by culturing strains overnight in YPD at 30°C followed by spotting 1.5 μL of 10-fold serial dilutions onto CSM, and CSM supplemented with 1 g/L of 5FOA (CSM+5FOA). Plates were incubated at 30°C for 3 days before imaging. In some experiments, the plates also contained either 10 mM or 30 mM HU [42].

### RNA isolation and quantitative real-time PCR (qRT–PCR)

RNA isolation and qRT-PCR for TLC1 and TERRA RNA levels were performed using published techniques [114,115]. Cells were grown in YPD to an OD600 of 0.6 to 0.8. 1 mL samples were used for RNA isolation with the RNeasy kit (Qiagen), with on-column DNase I treatment using the RNase-Free DNase Set (Qiagen). 1 μg RNA was reverse transcribed with the iScript cDNA Synthesis Kit (Bio-Rad), which uses random primers. cDNA was diluted 1:10 with distilled H_2_O. qPCR was performed with 2 μL of the dilution in a final volume of 20 μL using the iTaq Universal SYBR Green Supermix (Bio-Rad) in a Bio-Rad CFX96 Touch Real-Time PCR Detection System. Reaction conditions: 95°C for 10 min, 95°C for 15 sec, 50°C for 1 min, 40 cycles. Primer concentrations and sequences were the same as previously described [115].

### Immunoprecipitation

The μMACS anti-c-myc magnetic bead IP kit (Miltenyi Biotec) was used in immunoprecipitation experiments. Lysates were generated from strains in which one or two Paf1 complex genes in the *S*. *cerevisiae* strain BY4741 (*MATa leu2Δ0 his3Δ1 ura3Δ0 met15Δ0*) were tagged with Venus or c-myc. Strains were grown to mid-log phase in 50 mL YPD, harvested, resuspended in 1 mL of the supplied lysis buffer, and incubated on ice for 30 minutes. Cells were lysed with the addition of 100 μL of glass beads and vortexed four times for 1 minute with cooling. Lysates were clarified at 14,000 rpm for 10 minutes at 4°C. Protein concentrations were determined using the DC Protein Assay (Bio-Rad). For the input analysis, 500 μg of protein was trichloroacetic acid (TCA) precipitated, resuspended in 100 μL of 2x SDS gel loading buffer (100 mM Tris-Cl (pH 6.8), 4% SDS, 20% glycerol, 200 mM DTT, 0.2% bromophenol blue) and 10 μL was used for Western Blotting. For the immunoprecipitation, 1000 μg of protein was incubated with 50 μL anti-c-myc MicroBeads (Miltenyi Biotec) for 30 minutes on ice, then passed through the μMACS separator column. The column was washed twice with 200 μL of lysis buffer, washed twice with 200 μL of wash buffer 1, then washed once with 100 μL of wash buffer 2. The column was then incubated with 20 μL of heated elution buffer for 5 minutes, before the proteins were eluted with 50 μL of heated elution buffer. Of the eluted volume, 12 μL was used for Western Blotting.

### Western blotting

Proteins were resolved on a 4–15% SDS-PAGE gel (Bio-Rad) and transferred overnight onto nitrocellulose membrane (Bio-Rad). Venus-tagged proteins were detected with the rabbit monoclonal antibody ab290 (Abcam, 1:2000) and myc-tagged proteins were detected with 71D10 rabbit monoclonal antibody (Cell Signaling, 1:1000). Horseradish peroxidase-conjugated goat anti-rabbit secondary antibody (Jackson Laboratories, 1:5000) was used, followed by chemiluminescence detection with SuperSignal Femto Sensitivity Substrate (Life Technologies) and imaged with a Bio-Rad Imager. Venus-tagged protein levels were also detected using mouse monoclonal antibody B34 (Covance, 1:1000) and mouse monoclonal anti-Pgk1 antibody (ab113687, Abcam, 1:5000) was used to detect Pgk1 as a loading control.

### Live-cell imaging and image analysis

Exponentially growing cultures were washed and resuspended in water before being placed on minimal media agar pads, covered with a coverslip, and sealed with valap (a 1:1:1 mixture of Vaseline, lanolin, and paraffin by weight). Cells were imaged on a Deltavision (Applied Precision) microscope with an Olympus 100X 1.35NA objective. Fourteen 0.5 μm z sections were acquired and deconvolved with softWoRx software. Further image processing, including intensity measurements were performed using ImageJ. Intensity levels were quantified by taking the mean intensity in the nucleus, the cytoplasm, and a background measurement outside of the cell using a 3-pixel diameter circle. The ratio of background-subtracted nuclear fluorescence to background-subtracted cytoplasmic fluorescence was then calculated per cell. The total fluorescence was estimated by taking the background-subtracted nuclear fluorescence and adding it to 12.5 times the background-subtracted cytoplasmic fluorescence as an approximation of the ratio cytoplasmic to nuclear volume.

## Supporting information

S1 Fig Analysis of transcription elongation defects and loss of telomeric silencing in Paf1 complex subunit mutants. **a.** Ten-fold serial dilutions of log-phase cultures of strains with the indicated mutations in genes encoding Paf1 complex subunits were spotted onto non-selective complete synthetic medium (CSM), CSM + 50 μg/mL 6-azauracil (6-AU) to monitor for defects in transcriptional elongation, and CSM + 1 mg/mL 5-fluoroorotic acid (5FOA) to monitor for defects in silencing of a telomeric *URA3* gene. Plates were incubated at 30°C for 4 days before being photographed. **b.** The sensitivity of the *cdc73Δ* mutant to 5FOA in the TPE assay cannot be suppressed by sublethal concentrations of HU, indicating that loss of *CDC73* directly affects telomeric silencing rather indirectly causing 5FOA resistance through ribonucleotide reductase overexpression as seen for the *pol30-8* and *cac1Δ* alleles [42].
(PDF)

S2 Fig Identification of the starting chromosomal features on chromosome V by whole-genome sequencing. For each junction along chromosome V (junctions 5-A to 5-H), the evidence for each junction in the paired-end sequencing data is reported. The number preceding the slash is the number of junction-defining read pairs (those for which one read maps to one side of the junction and the other read maps to the other side of the junction). The number following the slash is the number of junction-sequencing reads (those that can be aligned to derive the sequence of the junction). “-/-” indicates a junction that could have been observed but was not observed, which is typically due to a GCR-related deletion. Note that some sequences are short enough that some read pairs span multiple junctions, e.g. junction 5-DE contains read pairs that span both junctions 5-D and 5-E.
(PDF)

S3 Fig Identification of the deletions of *CDC73*, *TEL1*, and *YKU80* by whole-genome sequencing. **Left.** Junctions are annotated as in S2 Fig with the addition that “n.a.” indicates a junction that could not have been observed as it was not present in the parental strain, such as the junctions associated with the deletions of *TEL1*, *CDC73* and *YKU80*. Right. Read depth analysis of the regions including *TEL1*, *CDC73*, and *YKU80* indicating that the expected deletions were observed for strains of each relevant genotype.
(PDF)

S4 Fig Identification of the starting chromosomal features on chromosomes other than chromosome V by whole-genome sequencing. Junctions are annotated as in S2 Fig.
(PDF)

S5 Fig Analysis of GCRs selected in the sGCR assay in a wild-type strain. Copy number analysis of the sequenced parental strain and GCR-containing strains shows that GCRs are associated with deletion of the *CAN1/URA3*-containing terminal portion of chromosome V L (left) and either duplication of a terminal region of a target chromosome or the junction sequence associated with a *de novo* telomere (right). The thick hashed blue arrow indicates sequences within the GCR; the thin dashed blue arrow indicates connectivity between portions of the GCR that map to different regions of the reference chromosome. Duplicated sequence involved in GCR-related HR events are shown as triangles; red triangles are Ty-related homologies and blue triangles are other homologies. Sequences in red correspond to the recovered sequence of the GCR junction; sequences in black are from the reference genome.
(PDF)

S6 Fig Analysis of GCRs selected in the sGCR assay in a *cdc73Δ* single mutant. Copy number analysis and breakpoint junction sequences of the sequenced parental strain and GCR-containing strains displayed as for S5 Fig. A hairpin-mediated inversion is indicated by the U-shaped arrow.
(PDF)

S7 Fig Analysis of GCRs produced in a *cdc73Δ tel1Δ* double mutant in the sGCR assay. Copy number analysis and breakpoint junction sequences of the sequenced parental strain and GCR-containing strains displayed as for S5 Fig and S6 Fig.
(PDF)

S8 Fig Analysis of GCRs selected in in the sGCR assay in a *cdc73Δ yku80Δ* double mutant. Copy number analysis and breakpoint junction sequences of the sequenced parental strain and GCR-containing strains displayed as for S5 Fig and S6 Fig.
(PDF)

S9 Fig Sequences of translocation junctions mediated by HR between the *P*_*LEU2*_*-NAT YCLWdelta5* fragment and Ty-related sequences elsewhere in the genome. **a, c, e, g.** Diagram of the HR event. **b, d, f, h.** Junction sequences and alignments between the GCR and the participating chromosomes identifies the novel junction sequences. Sequence of the junction between *YCLWdelta5* (yellow) and other delta sequence (red) that fuse chromosome V (light magenta) with the other target (light grey). Sequence that could have been derived from either *YCLWdelta5* or the other delta sequence is displayed with an orange background.
(PDF)

S10 Fig Sequences of translocation junctions mediated by HR between the *P*_*LEU2*_*-NAT SUP53* tRNA gene and tRNA genes elsewhere in the genome. **a, c.** Diagram of the HR event. **b, d.** Junction sequences and alignments between the GCR and the participating chromosomes identifies the novel junction sequences. Sequence of the junction between *SUP53* (blue) and other tRNA gene (green) that fuses chromosome V (light magenta) with the other target (light grey). Sequence that could have been derived from either *SUP53* or the other tRNA is displayed with a cyan background.
(PDF)

S11 Fig Sequences of *YCLWdelta5/YELWdelta1* junctions involved in formation of HR-mediated inverted duplications. **a.** Diagram of the HR event. **b.** Junction sequences displayed as in S9 Fig.
(PDF)

S12 Fig A mechanism that can explain the formation of the hairpin-mediated inverted duplication junctions observed. **a, c, e, g, i.** Inversion junction sequences identified in different GCRs. **b, d, f, h, j**. The inversion junction can be formed by 5’ resection from a DSB to generate a 3’-overhang. Intramolecular loop formation mediated by intra-strand base pairing generates a 3’ primer terminus that can be extended by DNA polymerases. This initial hairpin-capped chromosome will generate a dicentric chromosome upon replication, which is unstable and undergoes additional rounds of rearrangement. See Fig 7 for an alternative mechanism involving Break-induced Replication.
(PDF)

S13 Fig Sequences of secondary rearrangements involved in the resolution of inversion GCRs that are initially dicentric. **a, c, e, g, i, k, m, o.** Diagram of the secondary HR event. **b, d, f, h, j, l, n, p.** Junction sequences and alignments between the GCR and participating chromosomes identifies the novel junction sequences displayed as in S9 Fig. See Fig 7 for an alternative mechanism involving Break-induced Replication.
(PDF)

S14 Fig Identification of an unannotated delta, “*YELWdelta27*”, on the right arm of chromosome V. **a.** Dot plot comparing the sequence of *YELWdelta6* (y-axis) and chrV coordinates 448,000–450,000. All bases between the sequences are compared, regions of local similarity are shown as dots, and stretches of similar regions run diagonally. The annotated *YERCdelta20* is in the opposite orientation of the unannotated delta homology termed here “*YELWdelta27*”. **b.** Diagram of HR events between *YELWdelta1* and “*YELWdelta27*”. **c.** Junction sequences displayed as in S9 Fig.
(PDF)

S15 Fig Read depth histograms for *cdc73Δ yku80Δ* isolates containing a GCR. Copy number histograms for all sixteen chromosomes in the sequenced *cdc73Δ yku80Δ* GCR-containing isolates are shown. Duplicated chromosomes and chromosomal regions have twice the read depth as non-duplicated regions. Chromosomes duplicated by GCR-related events show a bimodal distribution (see for instance the partial duplication of chrXII in isolate 352). Only isolate 345 has a duplication of an entire chromosome, indicating disomy of chrXVI.
(PDF)

S16 Fig Evidence of senescence in the *cdc73Δ* single mutant and *cdc73Δ yku80Δ* and *cdc73Δ tel1Δ* double mutant strains. Sequential restreaking of freshly-generated *cdc73Δ* single mutant and *cdc73Δ yku80Δ* and *cdc73Δ tel1Δ* double mutant strains reveals a rapid loss in growth rate by restreak number 4, consistent with senescence due to defects in telomere maintenance. By restreak number 11, the *cdc73Δ* single mutant had an obvious improvement in growth, whereas the *cdc73Δ yku80Δ* and *cdc73Δ tel1Δ* double mutant strains continued to have growth defects and showed some recovery of growth but less than seen with the *cdc73Δ* single mutant. In contrast, the wild-type and *tel1Δ* single mutant strains showed no signs of growth defects.
(PDF)

S17 Fig Telomere defects in *cdc73Δ*, *tel1Δ*, and *yku80Δ* double mutants. **a.** TPE was tested in wild-type, *tel1Δ*, *yku80Δ*, *cdc73Δ*, *cdc73Δ tel1Δ*, and *cdc73Δ yku80Δ* strains with a telomeric *URA3* marker by monitoring growth of 10-fold serial dilutions strains on medium containing 5FOA. The sensitivity of strains with TPE defects could not be suppressed by sublethal concentrations of HU, indicating that loss of *CDC73* directly affects telomeric silencing rather indirectly causing 5FOA resistance through overexpression of ribonucleotide reductase as seen for the *pol30-8* and *cac1Δ* alleles [42]. **b.** TLC1 levels in wild-type, *tel1Δ*, *yku80Δ*, *cdc73Δ*, *cdc73Δ tel1Δ*, and *cdc73Δ yku80Δ* strains. Measurement of TLC1 was done in three biological replicates, and RNA levels were normalized against actin mRNA levels. Error bars are standard deviations. **c.** TERRA levels in wild-type, *tel1Δ*, *yku80Δ*, *cdc73Δ*, *cdc73Δ tel1Δ*, and *cdc73Δ yku80Δ* strains. Measurement of TERRA was done in triplicate and RNA levels relative to wild type of at least three independent biological replicates were normalized against actin mRNA levels. Error bars are standard deviations. One set of TERRA probes monitored TERRA expressed from 6 different telomeres that contain subtelomeric Y’ elements (6* Y’: from 8L, 8R, 12L, 12R, 13L, and 15R), and the other set of TERRA probes were specific to TERRA expressed from the telomere at the left arm of chromosome 15, which contains only X-elements. Primer concentrations and sequences were as previously described [115].
(PDF)

S18 Fig Only a subset of mutations causing short telomeres result in synergistic increases in GCR rates when combined with a *cdc73Δ* mutation. **a.** Genes in which mutations are known to cause short telomeres [46,47] and cause synergistic increases in GCR rates when combined with a *cdc73Δ* mutation as measured by patch tests in the dGCR assay [6] or as measured by fluctuation analysis in multiple GCR assays (Table 1). **b.** Genes in which mutations are known to cause short telomeres and do not cause synergistic increases in GCR rates when combined with a *cdc73Δ* mutation.
(PDF)

S19 Fig Introduction of a TLC1 overexpression plasmid causes higher TLC1 levels and increased the telomere length in the *cdc73Δ* single mutant and modestly increased lengths in the *cdc73Δ tel1Δ* double mutant. Southern blot of *Xho*I-digested genomic DNA isolated from strains of the indicated genotypes transformed either with an empty vector or with the TLC1 overexpression plasmid. TLC1 levels were also measured in these stains by quantitative reverse-transcription PCR. Introduction of the TLC1 overexpression plasmid increased the levels of TLC1 relative to the empty vector in all strains tested. In the wild-type strain raised the relative TLC1 levels from 1.0 ± 0.05 to 132.0 ± 5.3 fold, where the range is the standard error of the mean. In the *cdc73Δ* single mutant strain, the relative TLC1 levels raised from 0.2 ± 0.01 to 9.4 ± 0.3 fold. In the *cdc73Δ tel1Δ* double mutant strain, the relative TLC1 levels raised from 0.3 ± 0.02 to 6.9 ± 0.4 fold. In the *cdc73Δ yku80Δ* double mutant stain, the relative TLC1 levels raised from 0.5 ± 0.01 to 11.5 ± 0.7 fold.
(PDF)

S20 Fig Analysis of transcription elongation defects and loss of telomeric silencing in Paf1 complex subunit mutants. **a.** The *cdc73* truncation mutants that were tested in dilution analysis for defects in transcription elongation, as seen by sensitivity to 6-azauracil, and defects in telomeric silencing assessed in the telomere position effect assay, as seen by sensitivity to 5FOA. 10-fold dilutions of log phase cells were spotted onto non-selective complete synthetic medium (CSM), CSM + 50 μg/mL 6-azauracil, and CSM + 1 mg/mL 5FOA and incubated at 30°C for 4 days. The results are summarized in Fig 5A. **b.** To determine if the growth defects of cdc73 truncation mutations were due to overexpression of ribonucleotide reductase [42], the wild-type strain and strains containing the *cdc73Δ*, *cdc73Δ125–229*, and *cdc73*:*125–229* alleles were grown on CSM, CSM + 1 mg/mL 5FOA, and plates with sublethal concentrations of HU. Consistent with a true TPE defect, sublethal levels of HU did not restore growth on plates containing 5FOA.
(PDF)

S21 Fig Analysis of the minimal function region (residues 125–229) of Cdc73. **a.** The long-range disorder of the Cdc73 protein as predicted by IUPRED [82] reveals that the minimal functional region is more disordered on average than the folded C-terminal GTPase domain. **b.** Average conservation of residues in *S*. *cerevisiae* Cdc73 from an alignment of 191 fungal Cdc73 homologs generated by Clustal Omega [116] that reveals more extensive conservation in the C-terminal GTPase domain as well as a few conservation blocks in the N-terminal region. **c.** Chemical crosslinks to the minimal functional region of Cdc73 identified using the data of Xu et al. [81]. **d.** Lysates of *S*. *cerevisiae* strains containing a Paf1-myc fusion and a Cdc73-Venus fusion were subjected to immuneprecipitation with anti-myc antibodies, and the precipitated proteins were then probed with anti-Venus antibodies. Coimmunoprecipitation of Paf1 and Cdc73 was observed in the wild-type strain, the *leo1Δ* strain, and the *rtf1Δ* strain, but not the *ctr9Δ* strain.
(PDF)

S22 Fig The Paf1 complex has multiple nuclear localization signals. **a.** Cdc73 localizes to the nucleus in the absence of each other individual Paf1 complex subunit. Wild-type Cdc73 was tagged with Venus and each other Paf1 complex subunit was deleted, then cells were imaged by deconvolution microscopy. Scale bar is 2 μm. **b.** Cdc73 is not required for the nuclear localization of each other Paf1 complex subunit. Analogous to the experiments in panel a, each other complex subunit was tagged with Venus and imaged in a wild-type and *cdc73Δ* mutant strain. **c.** Deletion of *CDC73* has little if any effect on the levels of the other Paf1 complex subunits. For each of the strains shown in **b**, whole cell extracts were made by TCA extraction and individual complex subunit levels were determined by Western blot using an anti-GFP antibody. Pgk1 was monitored by Western blot with anti-Pgk1 antibodies as a loading control.
(PDF)

S23 Fig Computational predictions suggest nuclear localization signals are present in all Paf1 complex subunits except for Cdc73. Nuclear localization signals for the Paf1 complex subunits were predicted using cNLS Mapper (http://nls-mapper.iab.keio.ac.jp/cgi-bin/NLS_Mapper_form.cgi; [117]). Positions of the predicted signals are indicated in the subunit sequences (panels a, c, e, g, i), and the individual signals and their scores are displayed (panels b, d, f, h, j).
(PDF)

S1 Table Effect of deleting Paf1 complex-encoding genes on dGCR rates. (PDF)

S2 Table Effect of the *cdc73Δ* mutation on dGCR rates. (PDF)

S3 Table Statistics from whole genome sequencing of sGCR-containing isolates. (PDF)

S4 Table Summary of the evidence for the GCR-associated rearrangements in sGCR-containing isolates. (PDF)

S5 Table Effects of overexpressing *RNH1* and *TLC1* on genome instability in the uGCR assay. (PDF)

S6 Table Effect of *CDC73* truncations on dGCR rates. (PDF)

S7 Table *S*. *cerevisiae* strains used in this study. (PDF)
