# Supplementary figures and images for: Cdc73 suppresses genome instability by mediating telomere homeostasis
Source: PLoS Genet. 2018 Jan 10;14(1):e1007170. doi: 10.1371/journal.pgen.1007170 (PMC5779705; doi:10.1371/journal.pgen.1007170)

S1 Fig.

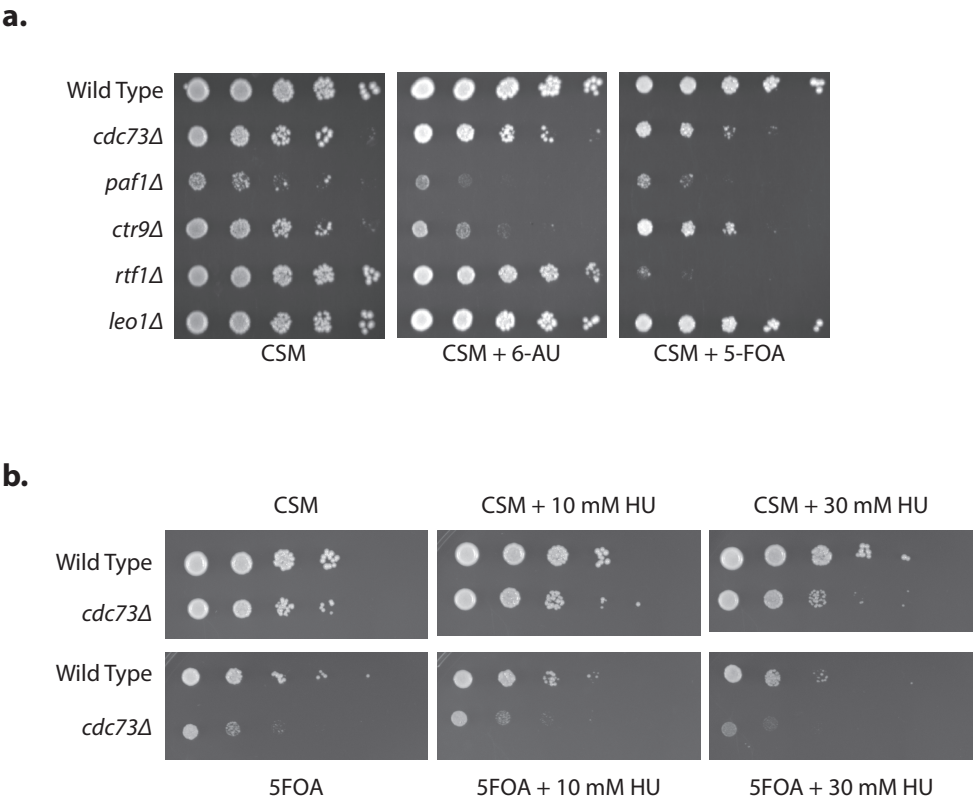

Supplement: S1 Fig — a. Ten-fold serial dilutions of log-phase cultures of strains with the indicated mutations in genes encoding Paf1 complex subunits were spotted onto non-selective complete synthetic medium (CSM), CSM + 50 μg/mL 6-azauracil (6-AU) to monitor for defects in transcriptional elongation, and CSM + 1 mg/mL 5-fluoroorotic acid (5FOA) to monitor for defects in silencing of a telomeric URA3 gene. Plates were incubated at 30°C for 4 days before being photographed. b. The sensitivity of the cdc73Δ mutant to 5FOA in the TPE assay cannot be suppressed by sublethal concentrations of HU, indicating that loss of CDC73 directly affects telomeric silencing rather indirectly causing 5FOA resistance through ribonucleotide reductase overexpression as seen for the pol30-8 and cac1Δ alleles [42]. (PDF) [file pgen.1007170.s001.pdf]

S2 Fig.

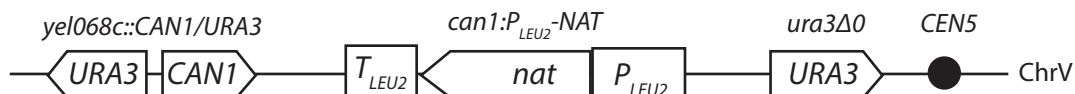[illegible]

Supplement: S2 Fig — For each junction along chromosome V (junctions 5-A to 5-H), the evidence for each junction in the paired-end sequencing data is reported. The number preceding the slash is the number of junction-defining read pairs (those for which one read maps to one side of the junction and the other read maps to the other side of the junction). The number following the slash is the number of junction-sequencing reads (those that can be aligned to derive the sequence of the junction). “-/-” indicates a junction that could have been observed but was not observed, which is typically due to a GCR-related deletion. Note that some sequences are short enough that some read pairs span multiple junctions, e.g. junction 5-DE contains read pairs that span both junctions 5-D and 5-E. (PDF) [file pgen.1007170.s002.pdf]

S5 Fig.

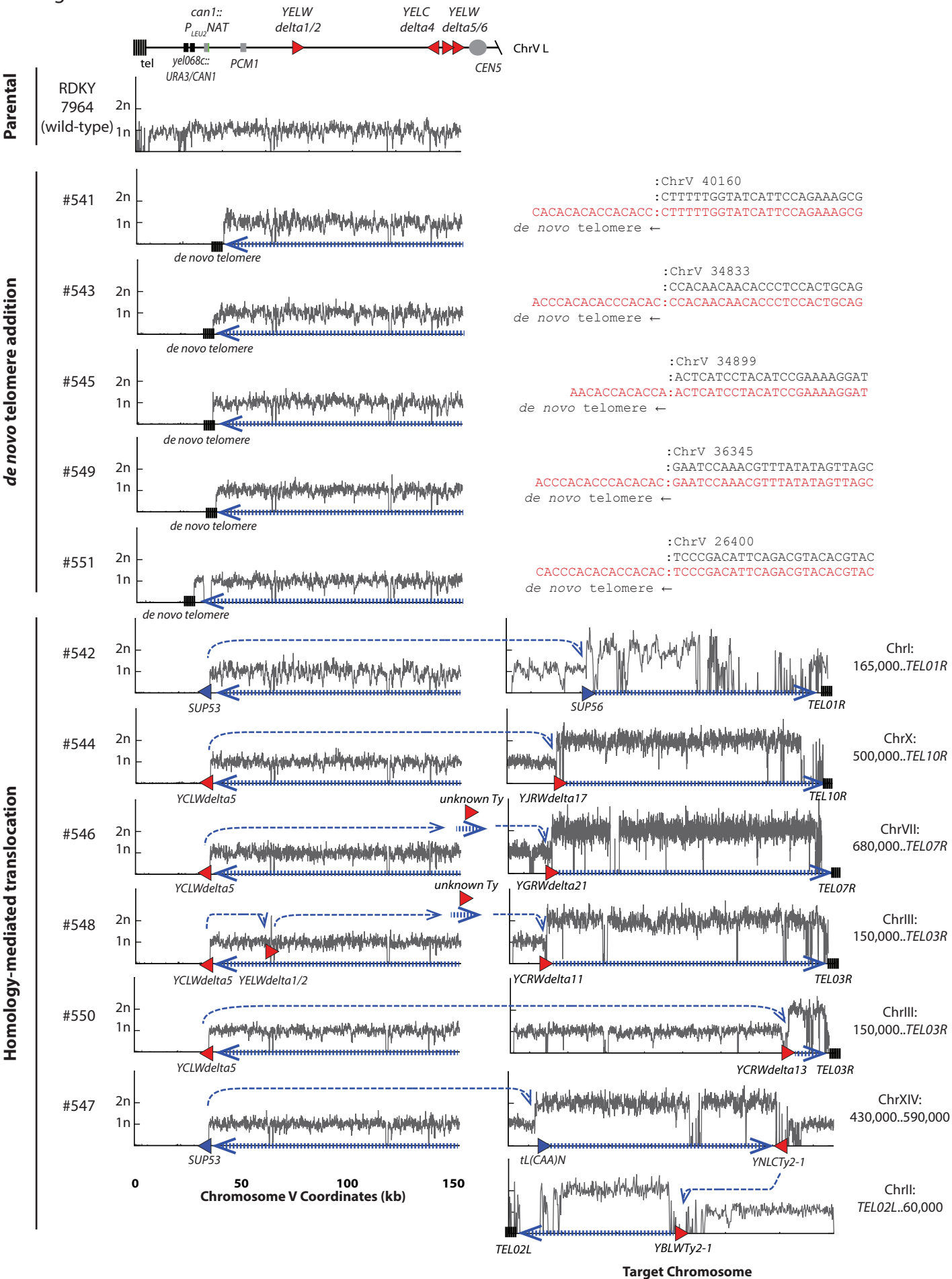

Supplement: S5 Fig — Copy number analysis of the sequenced parental strain and GCR-containing strains shows that GCRs are associated with deletion of the CAN1/URA3-containing terminal portion of chromosome V L (left) and either duplication of a terminal region of a target chromosome or the junction sequence associated with a de novo telomere (right). The thick hashed blue arrow indicates sequences within the GCR; the thin dashed blue arrow indicates connectivity between portions of the GCR that map to different regions of the reference chromosome. Duplicated sequence involved in GCR-related HR events are shown as triangles; red triangles are Ty-related homologies and blue triangles are other homologies. Sequences in red correspond to the recovered sequence of the GCR junction; sequences in black are from the reference genome. (PDF) [file pgen.1007170.s005.pdf]

S6 Fig.

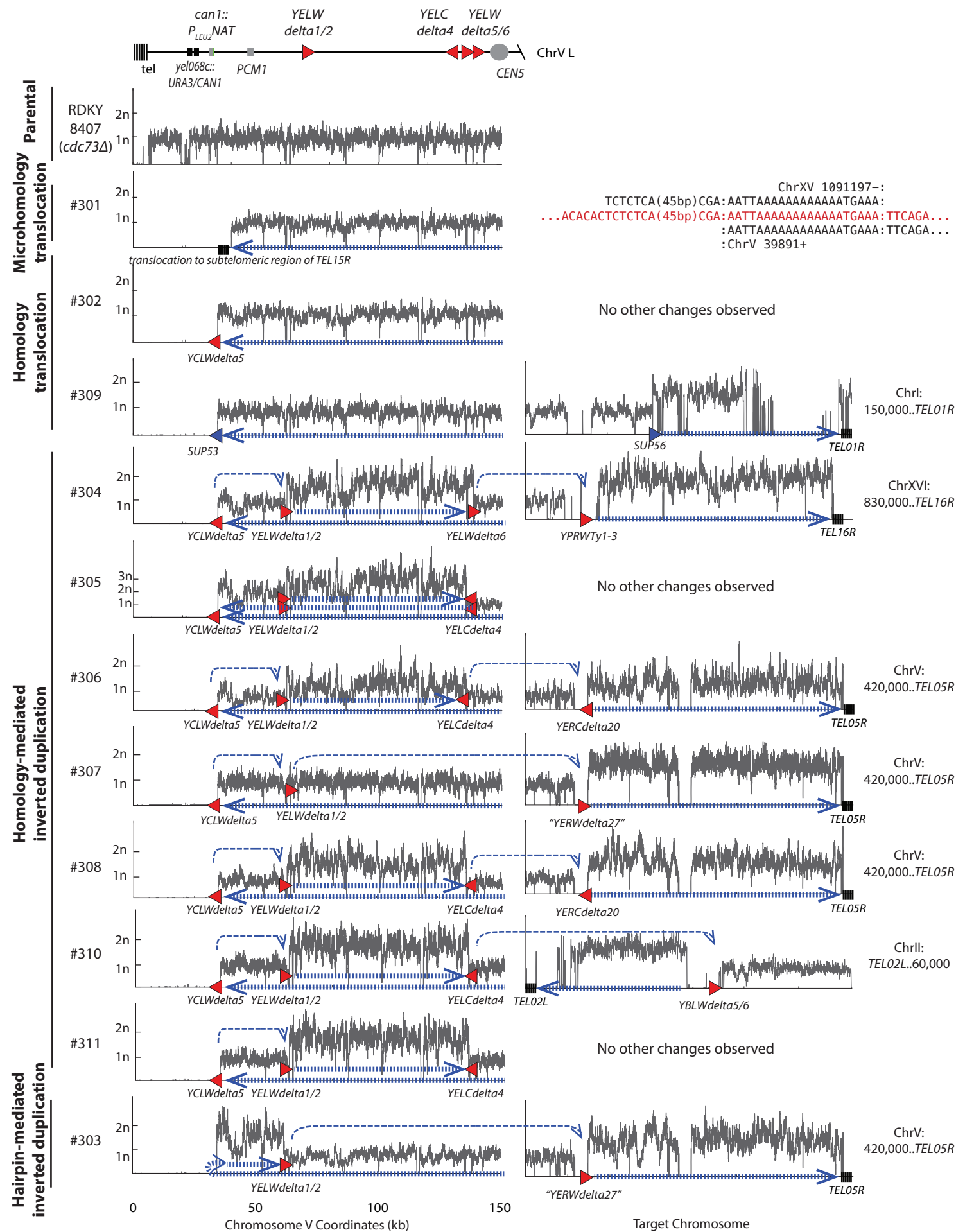

Supplement: S6 Fig — Copy number analysis and breakpoint junction sequences of the sequenced parental strain and GCR-containing strains displayed as for S5 Fig. A hairpin-mediated inversion is indicated by the U-shaped arrow. (PDF) [file pgen.1007170.s006.pdf]

S7 Fig.

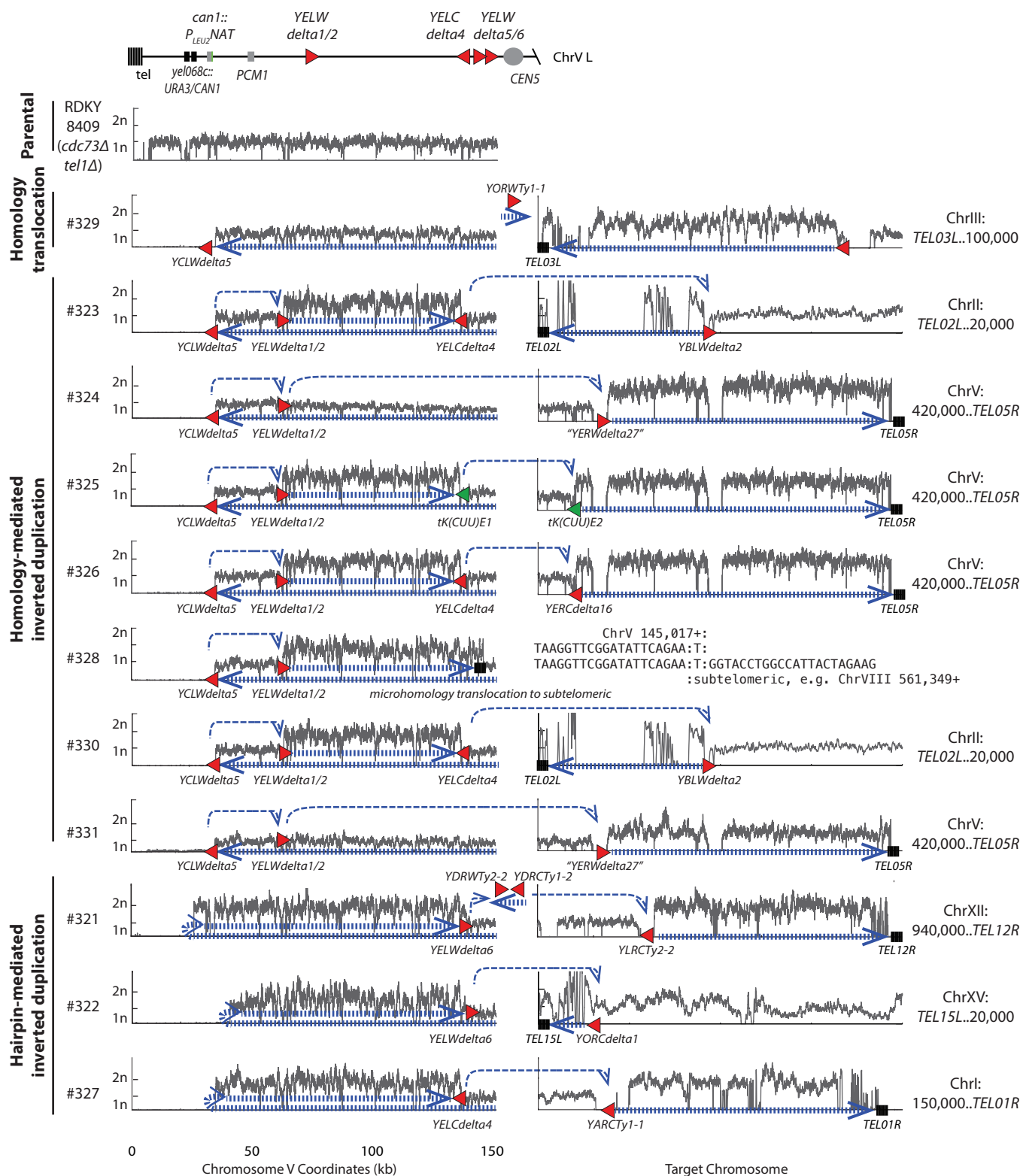

Supplement: S7 Fig — Copy number analysis and breakpoint junction sequences of the sequenced parental strain and GCR-containing strains displayed as for S5 Fig and S6 Fig. (PDF) [file pgen.1007170.s007.pdf]

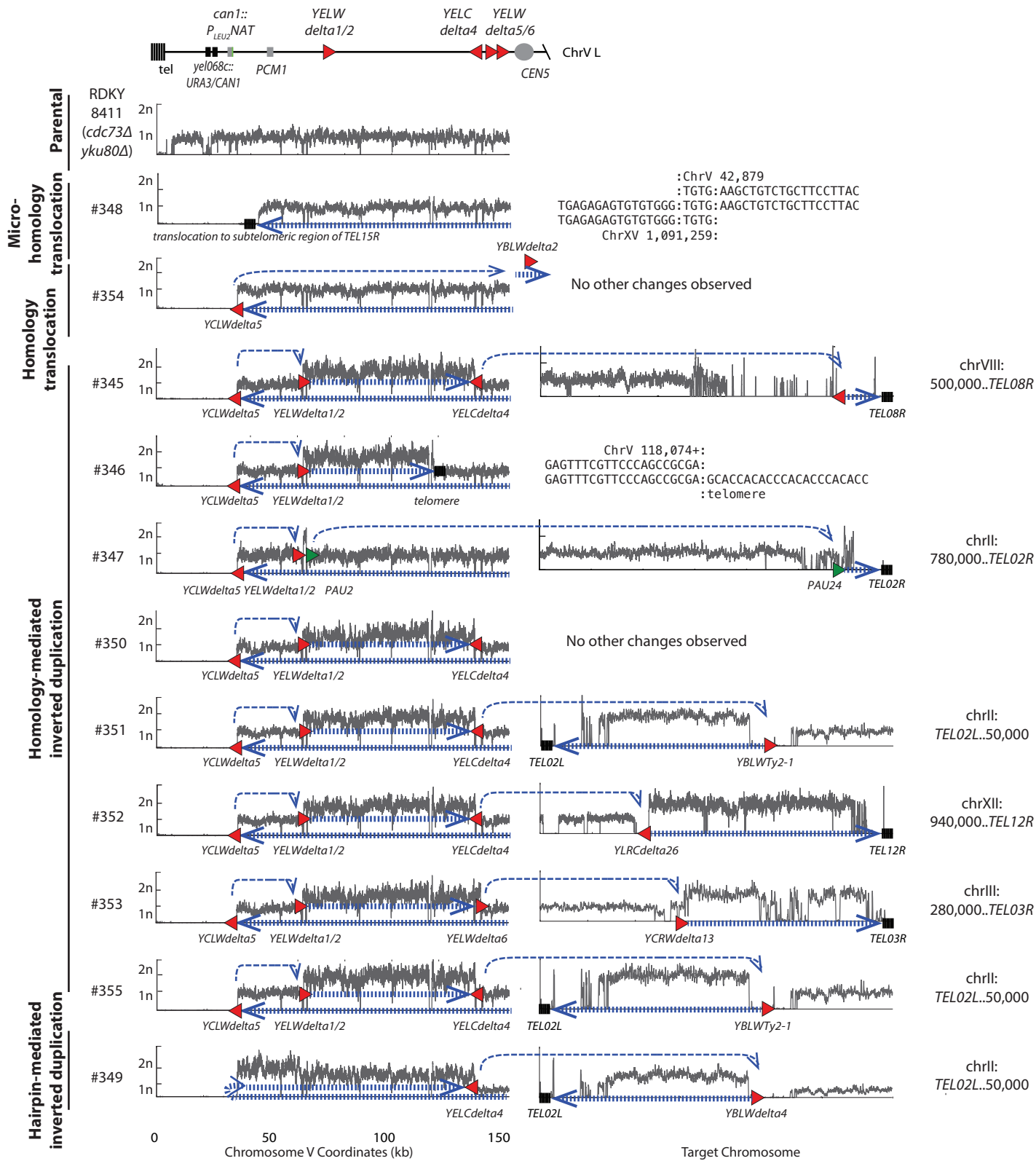

Supplement: S8 Fig — Copy number analysis and breakpoint junction sequences of the sequenced parental strain and GCR-containing strains displayed as for S5 Fig and S6 Fig. (PDF) [file pgen.1007170.s008.pdf]

S14 Fig.

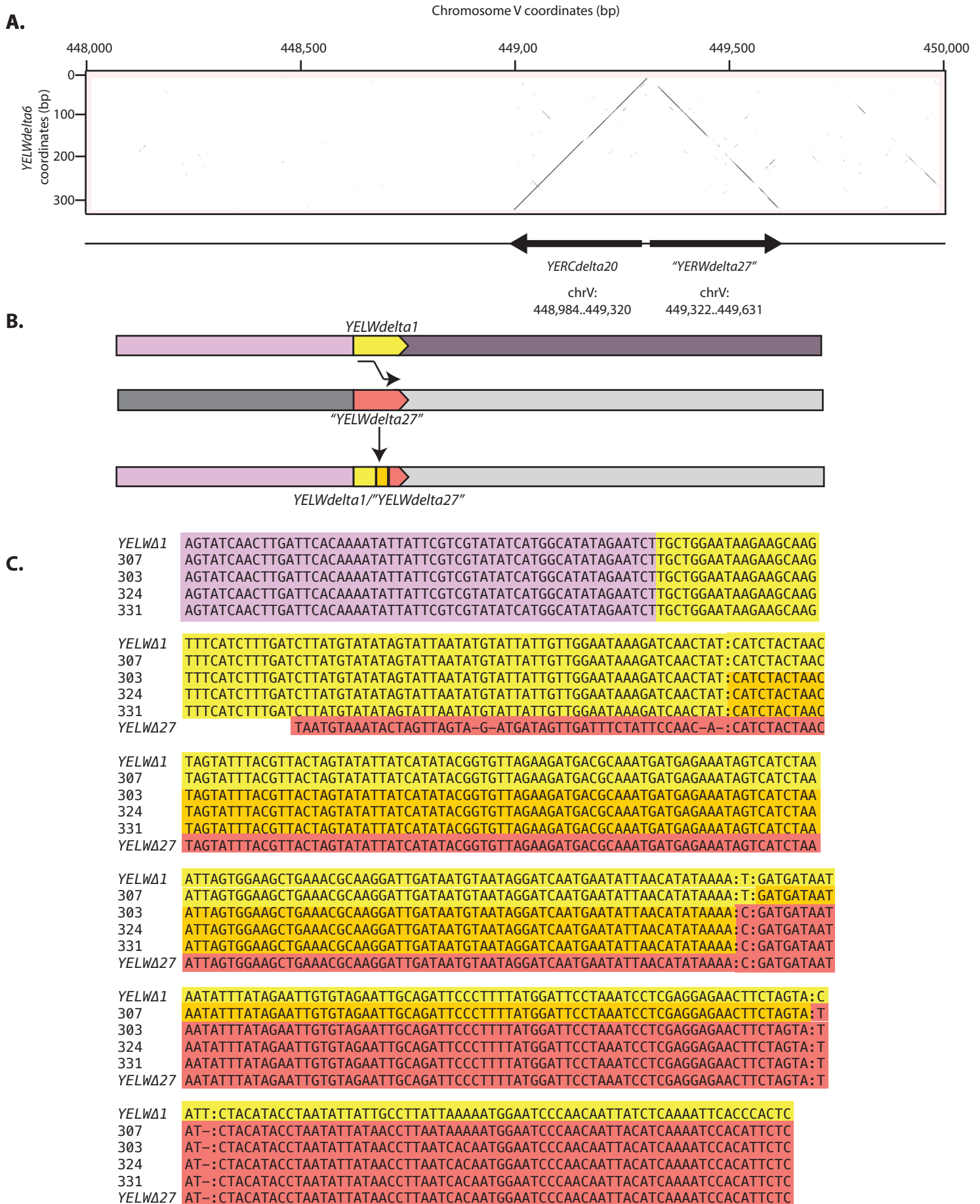

Supplement: S14 Fig — a. Dot plot comparing the sequence of YELWdelta6 (y-axis) and chrV coordinates 448,000–450,000. All bases between the sequences are compared, regions of local similarity are shown as dots, and stretches of similar regions run diagonally. The annotated YERCdelta20 is in the opposite orientation of the unannotated delta homology termed here “YELWdelta27”. b. Diagram of HR events between YELWdelta1 and “YELWdelta27”. c. Junction sequences displayed as in S9 Fig. (PDF) [file pgen.1007170.s014.pdf]

S15 Fig.

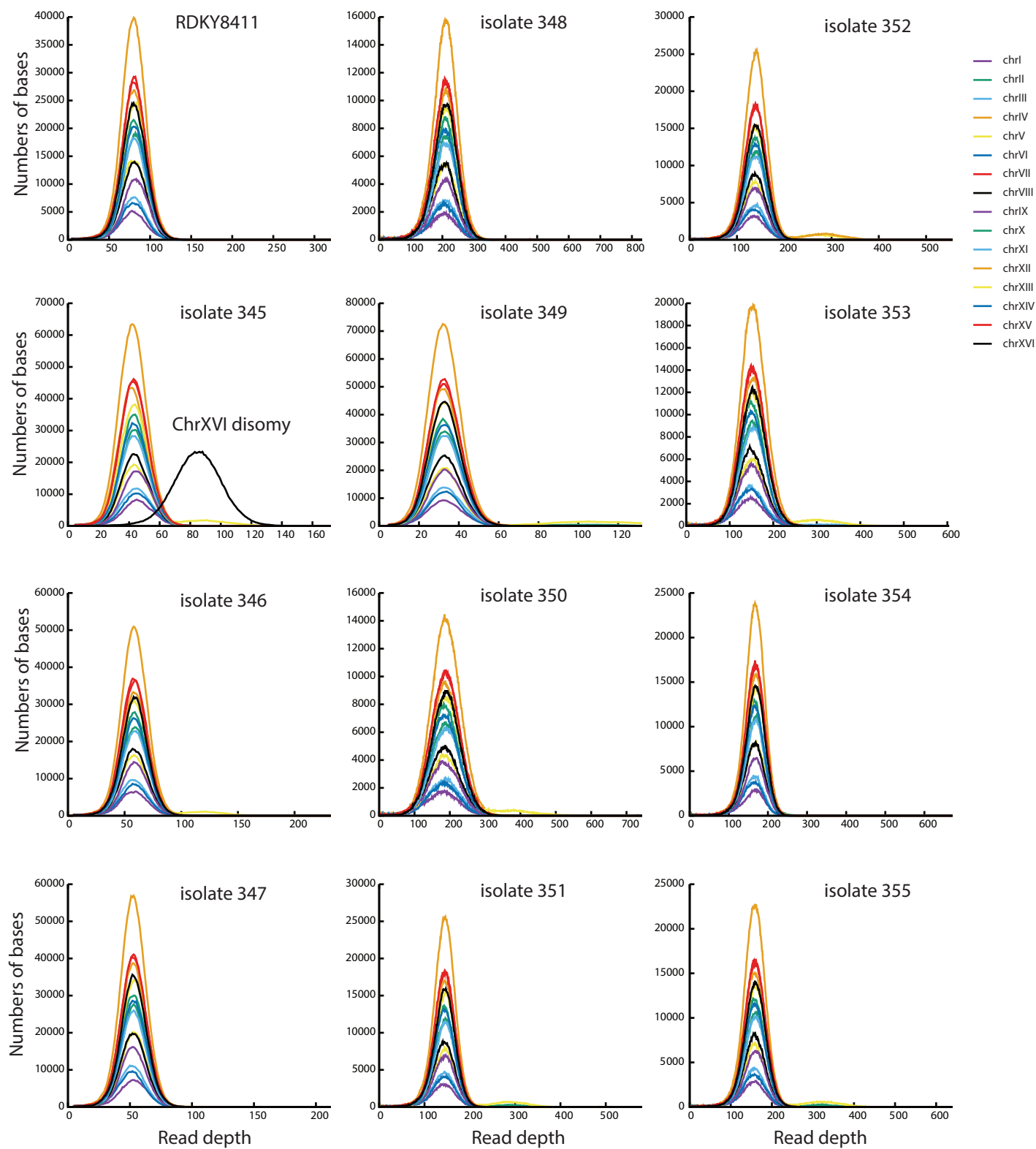

Supplement: S15 Fig — Copy number histograms for all sixteen chromosomes in the sequenced cdc73Δ yku80Δ GCR-containing isolates are shown. Duplicated chromosomes and chromosomal regions have twice the read depth as non-duplicated regions. Chromosomes duplicated by GCR-related events show a bimodal distribution (see for instance the partial duplication of chrXII in isolate 352). Only isolate 345 has a duplication of an entire chromosome, indicating disomy of chrXVI. (PDF) [file pgen.1007170.s015.pdf]

S16 Fig.

Streak 2

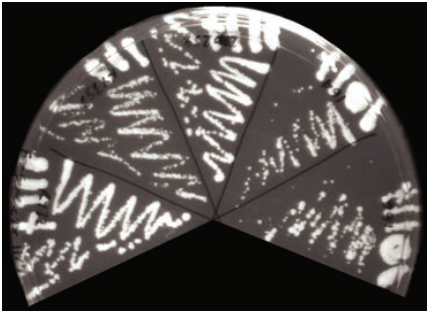

Streak 4

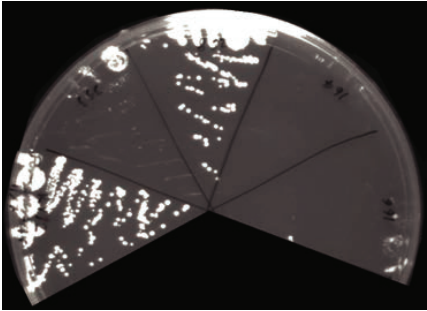

Streak 8

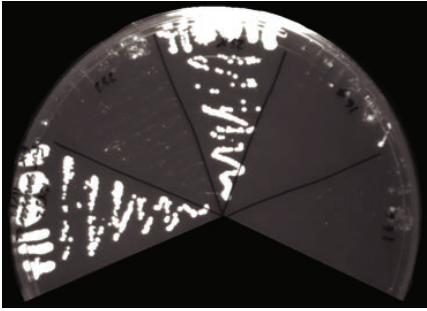

Streak 8

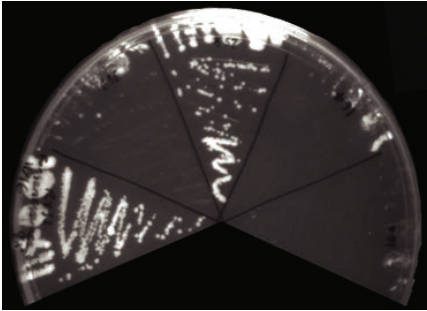

Streak 11

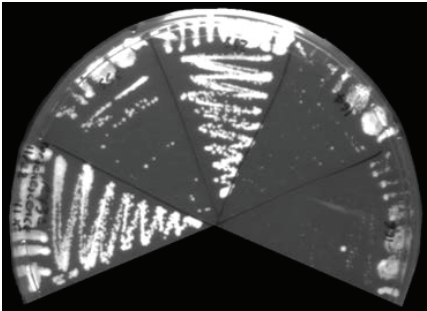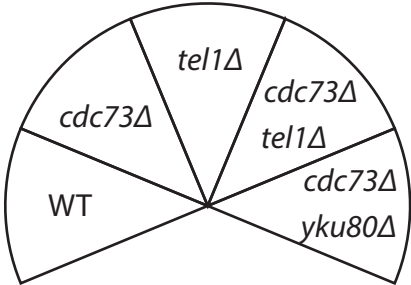

Supplement: S16 Fig — Sequential restreaking of freshly-generated cdc73Δ single mutant and cdc73Δ yku80Δ and cdc73Δ tel1Δ double mutant strains reveals a rapid loss in growth rate by restreak number 4, consistent with senescence due to defects in telomere maintenance. By restreak number 11, the cdc73Δ single mutant had an obvious improvement in growth, whereas the cdc73Δ yku80Δ and cdc73Δ tel1Δ double mutant strains continued to have growth defects and showed some recovery of growth but less than seen with the cdc73Δ single mutant. In contrast, the wild-type and tel1Δ single mutant strains showed no signs of growth defects. (PDF) [file pgen.1007170.s016.pdf]

S17 Fig.

**a.**

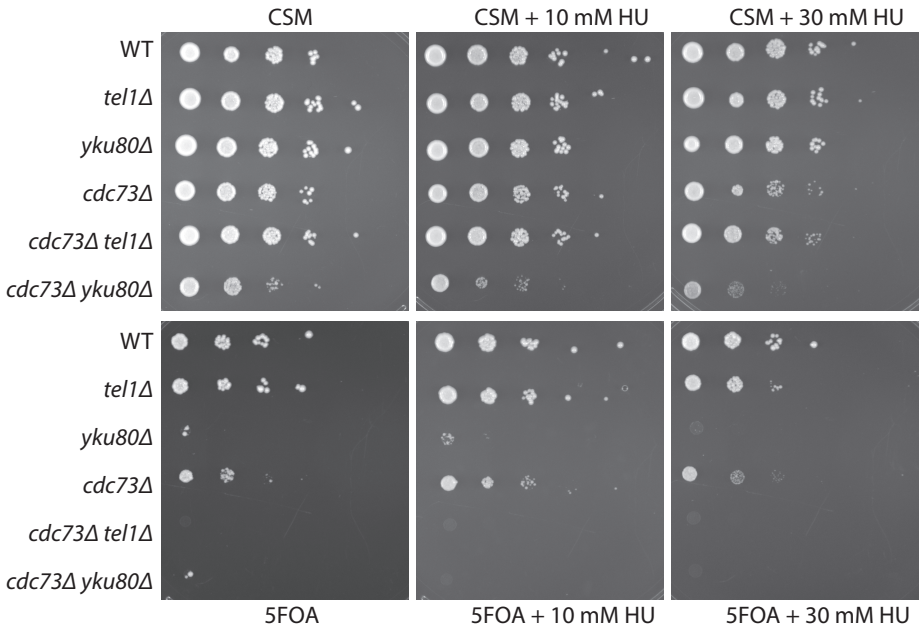

**b.**

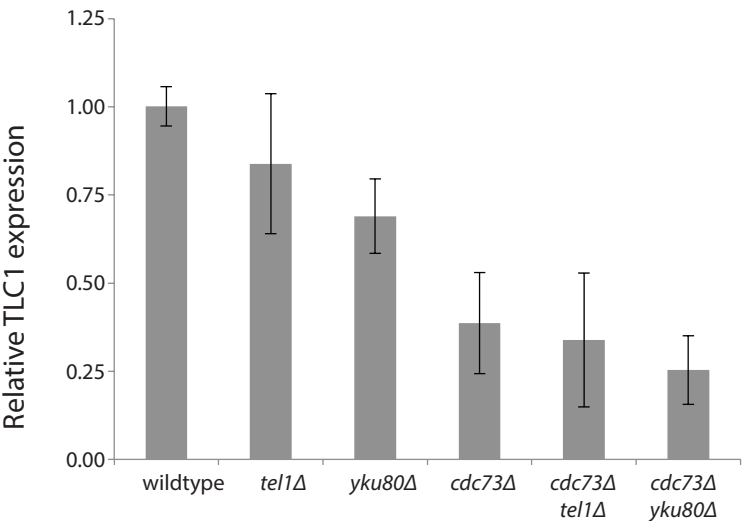

**c.**

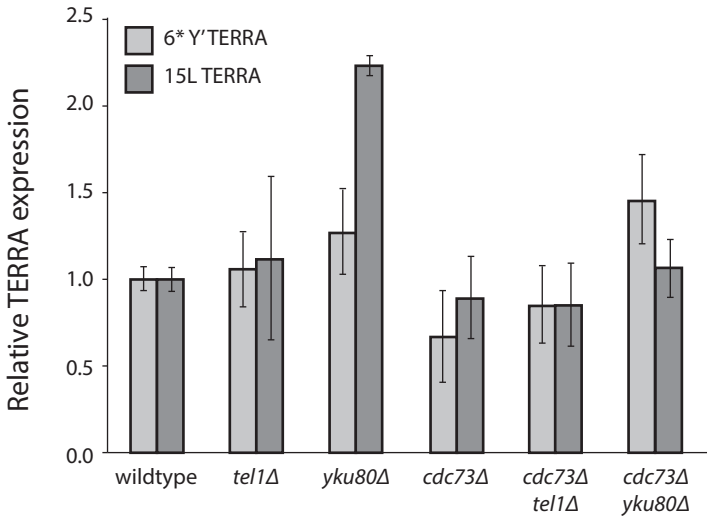

Supplement: S17 Fig — a. TPE was tested in wild-type, tel1Δ, yku80Δ, cdc73Δ, cdc73Δ tel1Δ, and cdc73Δ yku80Δ strains with a telomeric URA3 marker by monitoring growth of 10-fold serial dilutions strains on medium containing 5FOA. The sensitivity of strains with TPE defects could not be suppressed by sublethal concentrations of HU, indicating that loss of CDC73 directly affects telomeric silencing rather indirectly causing 5FOA resistance through overexpression of ribonucleotide reductase as seen for the pol30-8 and cac1Δ alleles [42]. b. TLC1 levels in wild-type, tel1Δ, yku80Δ, cdc73Δ, cdc73Δ tel1Δ, and cdc73Δ yku80Δ strains. Measurement of TLC1 was done in three biological replicates, and RNA levels were normalized against actin mRNA levels. Error bars are standard deviations. c. TERRA levels in wild-type, tel1Δ, yku80Δ, cdc73Δ, cdc73Δ tel1Δ, and cdc73Δ yku80Δ strains. Measurement of TERRA was done in triplicate and RNA levels relative to wild type of at least three independent biological replicates were normalized against actin mRNA levels. Error bars are standard deviations. One set of TERRA probes monitored TERRA expressed from 6 different telomeres that contain subtelomeric Y’ elements (6* Y’: from 8L, 8R, 12L, 12R, 13L, and 15R), and the other set of TERRA probes were specific to TERRA expressed from the telomere at the left arm of chromosome 15, which contains only X-elements. Primer concentrations and sequences were as previously described [115]. (PDF) [file pgen.1007170.s017.pdf]

S19 Fig.

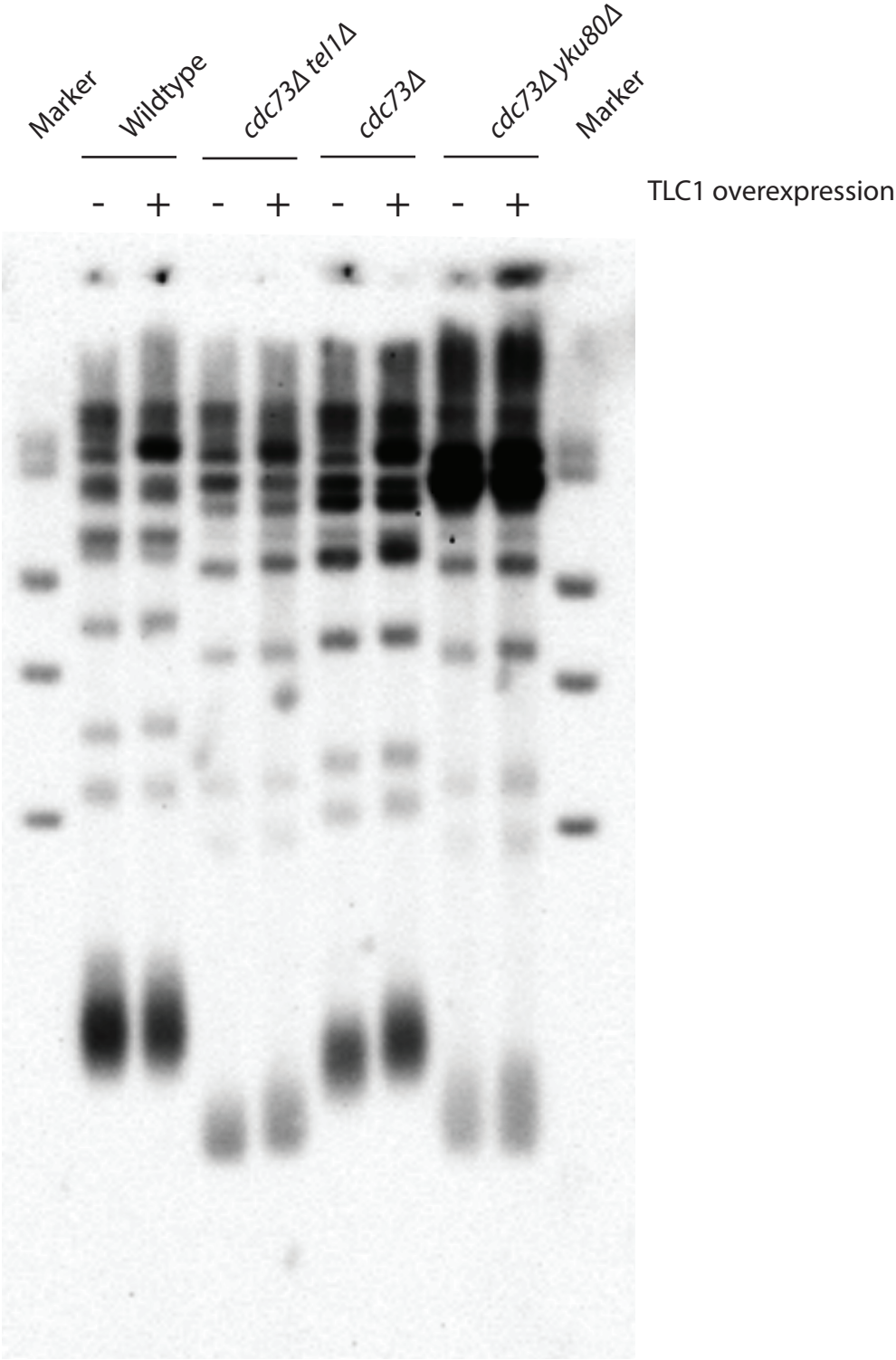

Supplement: S19 Fig — Southern blot of XhoI-digested genomic DNA isolated from strains of the indicated genotypes transformed either with an empty vector or with the TLC1 overexpression plasmid. TLC1 levels were also measured in these stains by quantitative reverse-transcription PCR. Introduction of the TLC1 overexpression plasmid increased the levels of TLC1 relative to the empty vector in all strains tested. In the wild-type strain raised the relative TLC1 levels from 1.0 ± 0.05 to 132.0 ± 5.3 fold, where the range is the standard error of the mean. In the cdc73Δ single mutant strain, the relative TLC1 levels raised from 0.2 ± 0.01 to 9.4 ± 0.3 fold. In the cdc73Δ tel1Δ double mutant strain, the relative TLC1 levels raised from 0.3 ± 0.02 to 6.9 ± 0.4 fold. In the cdc73Δ yku80Δ double mutant stain, the relative TLC1 levels raised from 0.5 ± 0.01 to 11.5 ± 0.7 fold. (PDF) [file pgen.1007170.s019.pdf]

S20 Fig.

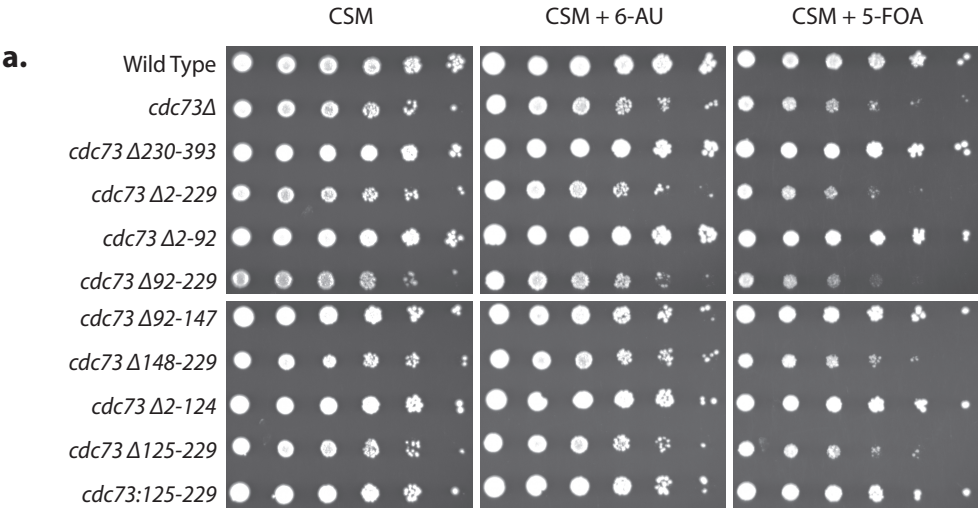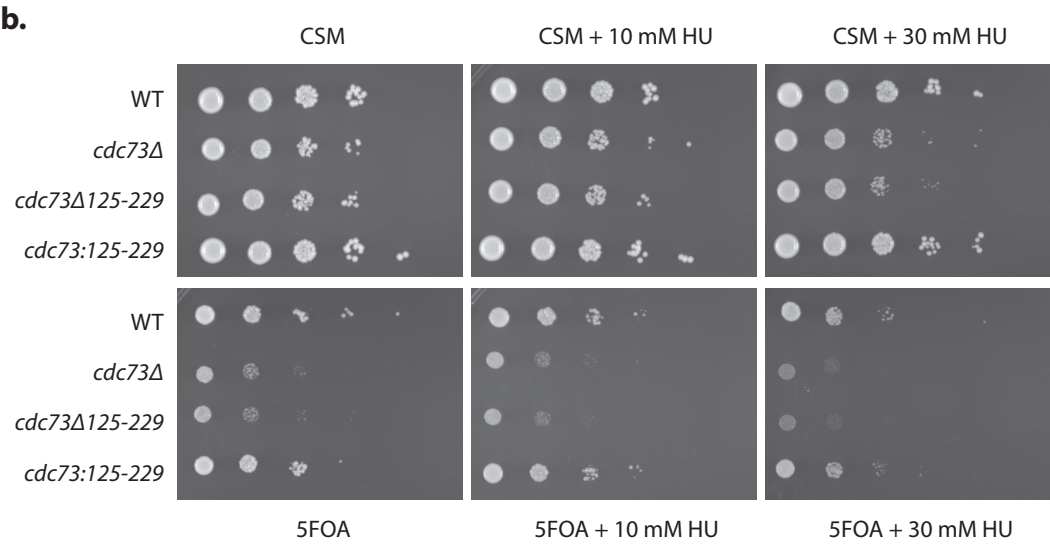

Supplement: S20 Fig — a. The cdc73 truncation mutants that were tested in dilution analysis for defects in transcription elongation, as seen by sensitivity to 6-azauracil, and defects in telomeric silencing assessed in the telomere position effect assay, as seen by sensitivity to 5FOA. 10-fold dilutions of log phase cells were spotted onto non-selective complete synthetic medium (CSM), CSM + 50 μg/mL 6-azauracil, and CSM + 1 mg/mL 5FOA and incubated at 30°C for 4 days. The results are summarized in Fig 5A. b. To determine if the growth defects of cdc73 truncation mutations were due to overexpression of ribonucleotide reductase [42], the wild-type strain and strains containing the cdc73Δ, cdc73Δ125–229, and cdc73:125–229 alleles were grown on CSM, CSM + 1 mg/mL 5FOA, and plates with sublethal concentrations of HU. Consistent with a true TPE defect, sublethal levels of HU did not restore growth on plates containing 5FOA. (PDF) [file pgen.1007170.s020.pdf]

S21 Fig.

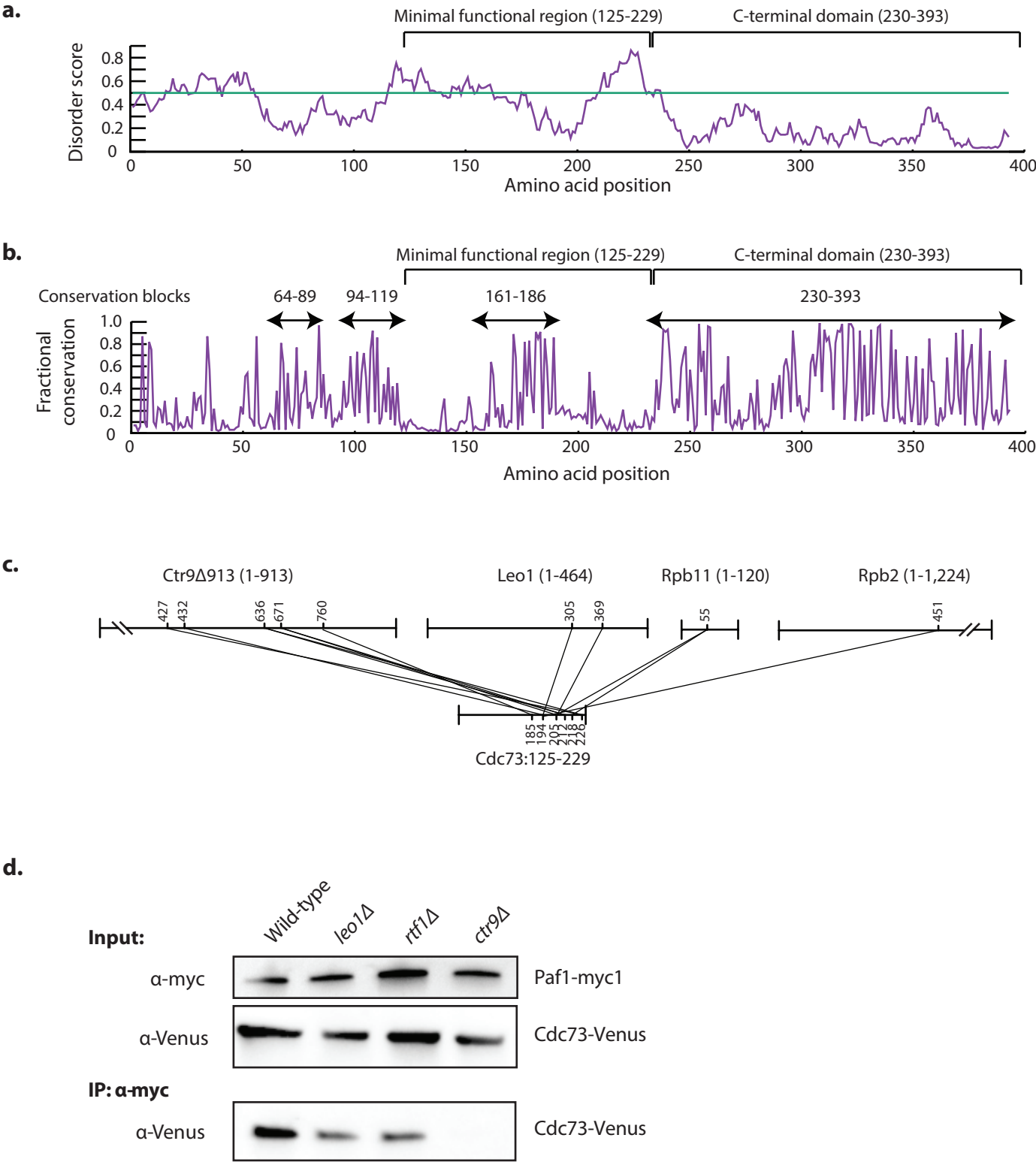

Supplement: S21 Fig — a. The long-range disorder of the Cdc73 protein as predicted by IUPRED [82] reveals that the minimal functional region is more disordered on average than the folded C-terminal GTPase domain. b. Average conservation of residues in S. cerevisiae Cdc73 from an alignment of 191 fungal Cdc73 homologs generated by Clustal Omega [116] that reveals more extensive conservation in the C-terminal GTPase domain as well as a few conservation blocks in the N-terminal region. c. Chemical crosslinks to the minimal functional region of Cdc73 identified using the data of Xu et al. [81]. d. Lysates of S. cerevisiae strains containing a Paf1-myc fusion and a Cdc73-Venus fusion were subjected to immuneprecipitation with anti-myc antibodies, and the precipitated proteins were then probed with anti-Venus antibodies. Coimmunoprecipitation of Paf1 and Cdc73 was observed in the wild-type strain, the leo1Δ strain, and the rtf1Δ strain, but not the ctr9Δ strain. (PDF) [file pgen.1007170.s021.pdf]

S22 Fig.

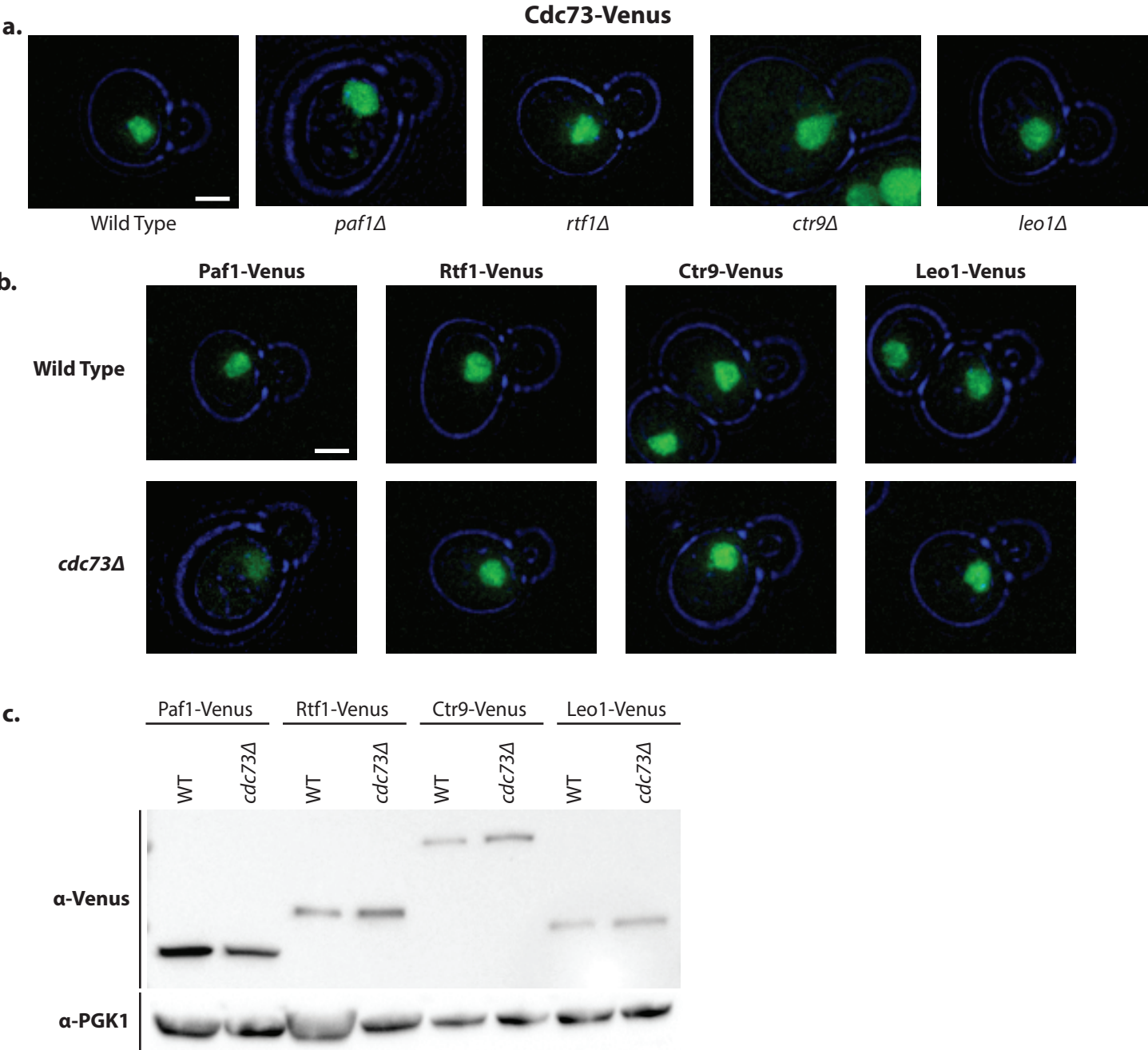

Supplement: S22 Fig — a. Cdc73 localizes to the nucleus in the absence of each other individual Paf1 complex subunit. Wild-type Cdc73 was tagged with Venus and each other Paf1 complex subunit was deleted, then cells were imaged by deconvolution microscopy. Scale bar is 2 μm. b. Cdc73 is not required for the nuclear localization of each other Paf1 complex subunit. Analogous to the experiments in panel a, each other complex subunit was tagged with Venus and imaged in a wild-type and cdc73Δ mutant strain. c. Deletion of CDC73 has little if any effect on the levels of the other Paf1 complex subunits. For each of the strains shown in b, whole cell extracts were made by TCA extraction and individual complex subunit levels were determined by Western blot using an anti-GFP antibody. Pgk1 was monitored by Western blot with anti-Pgk1 antibodies as a loading control. (PDF) [file pgen.1007170.s022.pdf]
