# Supplementary material for: Cdc73 suppresses genome instability by mediating telomere homeostasis
Source: PLoS Genet. 2018 Jan 10;14(1):e1007170. doi: 10.1371/journal.pgen.1007170 (PMC5779705; doi:10.1371/journal.pgen.1007170)

S3 Fig.

	Junction:	ChrII *tel1::G418* *TEL1* 2-A	2-B	ChrXII *cdc73::HIS3* *CDC73* 12-A	12-B	ChrXIII *yku80::G418* *YKU80* 13-A	13-B
wt	RDKY7964	n.a.	n.a.	n.a.	n.a.	n.a.	n.a.
	isolate 541	n.a.	n.a.	n.a.	n.a.	n.a.	n.a.
	isolate 542	n.a.	n.a.	n.a.	n.a.	n.a.	n.a.
	isolate 543	n.a.	n.a.	n.a.	n.a.	n.a.	n.a.
	isolate 544	n.a.	n.a.	n.a.	n.a.	n.a.	n.a.
	isolate 545	n.a.	n.a.	n.a.	n.a.	n.a.	n.a.
	isolate 546	n.a.	n.a.	n.a.	n.a.	n.a.	n.a.
	isolate 547	n.a.	n.a.	n.a.	n.a.	n.a.	n.a.
	isolate 548	n.a.	n.a.	n.a.	n.a.	n.a.	n.a.
	isolate 549	n.a.	n.a.	n.a.	n.a.	n.a.	n.a.
	isolate 550	n.a.	n.a.	n.a.	n.a.	n.a.	n.a.
	isolate 551	n.a.	n.a.	n.a.	n.a.	n.a.	n.a.
cdc73	RDKY8407	n.a.	n.a.	158/92	160/60	n.a.	n.a.
	isolate 301	n.a.	n.a.	303/182	392/160	n.a.	n.a.
	isolate 302	n.a.	n.a.	370/250	609/213	n.a.	n.a.
	isolate 303	n.a.	n.a.	182/107	249/84	n.a.	n.a.
	isolate 304	n.a.	n.a.	175/126	231/93	n.a.	n.a.
	isolate 305	n.a.	n.a.	140/80	158/64	n.a.	n.a.
	isolate 306	n.a.	n.a.	66/52	80/37	n.a.	n.a.
	isolate 307	n.a.	n.a.	126/89	176/40	n.a.	n.a.
	isolate 308	n.a.	n.a.	87/78	98/30	n.a.	n.a.
	isolate 309	n.a.	n.a.	109/87	149/43	n.a.	n.a.
	isolate 310	n.a.	n.a.	95/38	103/34	n.a.	n.a.
	isolate 311	n.a.	n.a.	100/68	122/38	n.a.	n.a.
cdc73	RDKY8409	310/84	354/77	247/153	308/107	n.a.	n.a.
tel1	isolate 321	282/77	305/76	206/123	284/90	n.a.	n.a.
	isolate 322	126/30	266/61	127/65	168/30	n.a.	n.a.
	isolate 323	252/72	272/66	212/119	236/65	n.a.	n.a.
	isolate 324	492/121	571/141	371/179	391/130	n.a.	n.a.
	isolate 325	342/77	345/83	215/135	287/90	n.a.	n.a.
	isolate 326	415/108	468/111	314/233	433/181	n.a.	n.a.
	isolate 327	179/27	284/75	140/120	178/79	n.a.	n.a.
	isolate 328	305/69	413/111	233/183	262/91	n.a.	n.a.
	isolate 329	168/48	264/62	143/104	166/69	n.a.	n.a.
	isolate 330	244/72	246/67	168/126	220/87	n.a.	n.a.
	isolate 331	106/31	209/57	131/29	89/120	n.a.	n.a.
cdc73	RDKY8411	n.a.	n.a.	115/123	159/61	163/41	189/57
yku80	isolate 345	n.a.	n.a.	66/50	84/51	101/20	83/23
	isolate 346	n.a.	n.a.	100/75	129/53	113/24	122/41
	isolate 347	n.a.	n.a.	83/61	119/45	114/26	126/37
	isolate 348	n.a.	n.a.	323/200	479/150	434/113	394/134
	isolate 349	n.a.	n.a.	51/46	56/34	68/17	87/22
	isolate 350	n.a.	n.a.	279/179	402/138	355/78	271/95
	isolate 351	n.a.	n.a.	200/149	289/126	277/77	251/81
	isolate 352	n.a.	n.a.	199/135	352/165	315/74	283/89
	isolate 353	n.a.	n.a.	236/163	351/134	330/61	293/85
	isolate 354	n.a.	n.a.	258/202	402/165	388/84	350/102
	isolate 355	n.a.	n.a.	198/176	330/148	304/61	305/107

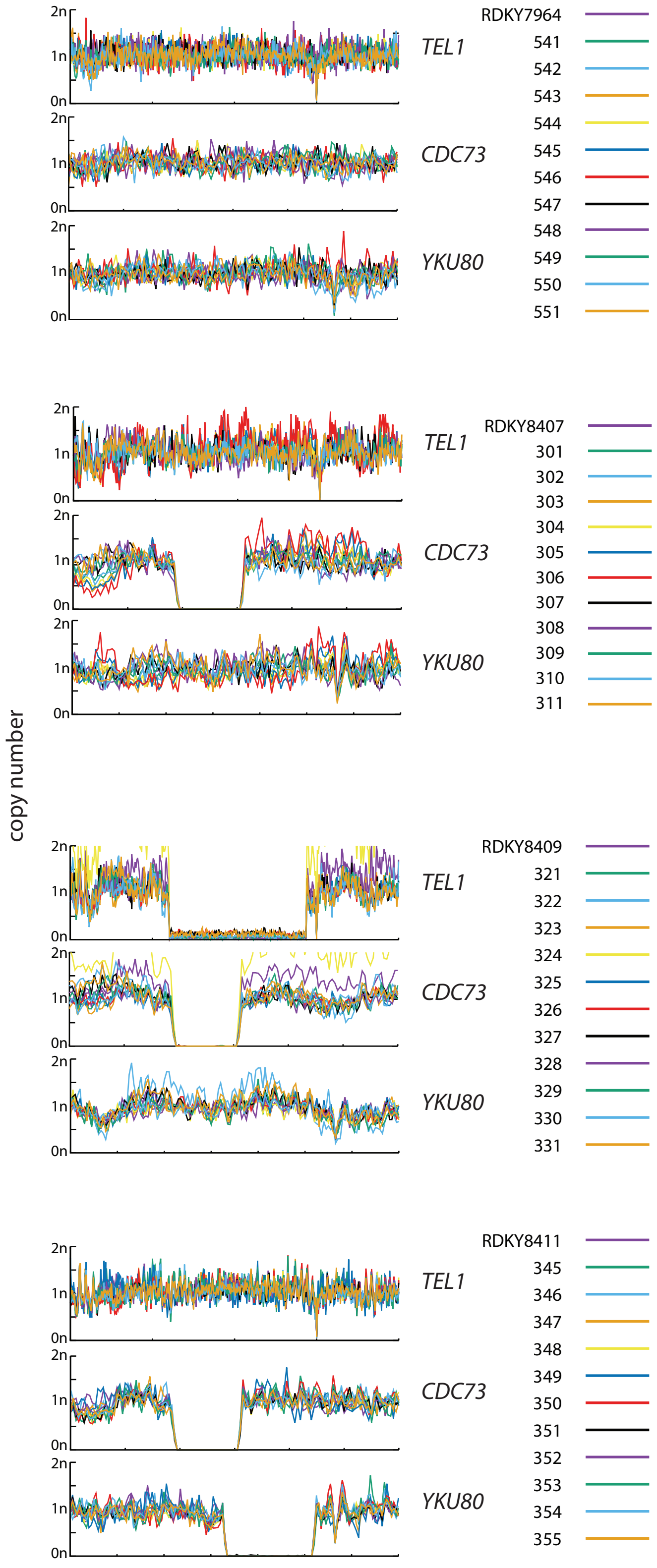

Supplement: S3 Fig — Left. Junctions are annotated as in S2 Fig with the addition that “n.a.” indicates a junction that could not have been observed as it was not present in the parental strain, such as the junctions associated with the deletions of TEL1, CDC73 and YKU80. Right. Read depth analysis of the regions including TEL1, CDC73, and YKU80 indicating that the expected deletions were observed for strains of each relevant genotype. (PDF) [file pgen.1007170.s003.pdf]
