# Supplementary material for: Cdc73 suppresses genome instability by mediating telomere homeostasis
Source: PLoS Genet. 2018 Jan 10;14(1):e1007170. doi: 10.1371/journal.pgen.1007170 (PMC5779705; doi:10.1371/journal.pgen.1007170)
Supplement: S4 Fig — Junctions are annotated as in S2 Fig. (PDF) [file pgen.1007170.s004.pdf]

S4 Fig.

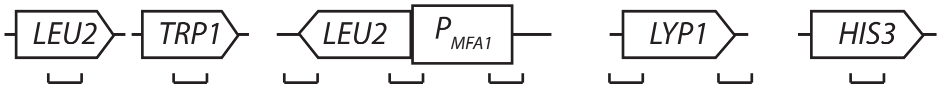

	Junction:	3-A	4-A	6-A	6-B	6-C	14-A	14-B	15-A
		ChrIII *leu2Δ0*	ChrIV *trp1Δ63*	ChrVI *iYFR016C::PMFA1-LEU2*			ChrXIV *lyp1::TRP1*		ChrXV *his3Δ200*
wt	RDKY7964	68/16	137/37	179/49	187/43	168/84	90/64	58/166	183/19
	isolate 541	95/23	129/34	160/69	199/31	196/105	127/66	73/146	158/26
	isolate 542	58/13	147/22	183/61	179/29	205/79	92/58	63/136	210/19
	isolate 543	74/14	136/26	158/64	193/21	154/61	88/45	67/139	176/30
	isolate 544	102/26	162/36	186/55	210/32	156/44	123/46	112/153	205/22
	isolate 545	98/32	149/24	182/59	197/24	177/64	129/52	96/126	222/20
	isolate 546	57/12	114/22	114/49	144/17	107/40	89/48	60/108	114/21
	isolate 547	77/17	143/20	136/53	172/32	164/62	118/41	85/137	137/12
	isolate 548	83/20	154/14	130/39	153/19	145/36	104/60	81/115	116/9
	isolate 549	109/27	160/29	202/60	184/27	176/67	124/66	64/166	166/22
	isolate 550	81/25	212/47	229/76	233/39	210/97	168/76	126/173	246/24
	isolate 551	91/32	148/27	179/57	162/38	162/65	117/61	87/109	188/23
cdc73	RDKY8407	108/20	184/28	170/52	207/33	185/98	129/56	65/139	157/16
	isolate 301	410/85	449/108	367/153	460/98	427/217	338/171	64/330	402/68
	isolate 302	565/76	594/148	543/191	642/127	595/252	519/230	95/127	607/108
	isolate 303	211/42	253/55	171/88	299/65	258/130	202/92	33/65	238/50
	isolate 304	286/49	276/59	201/76	292/52	245/149	284/103	45/226	226/38
	isolate 305	196/37	199/39	118/56	181/47	165/104	178/91	22/39	165/31
	isolate 306	118/33	105/26	53/35	97/21	79/54	89/50	12/81	73/12
	isolate 307	43/17	168/32	185/50	210/37	156/79	121/74	60/134	167/30
	isolate 308	60/19	90/34	138/51	152/22	166/75	62/62	33/133	98/26
	isolate 309	112/26	133/37	159/72	174/40	173/102	108/54	38/163	170/23
	isolate 310	73/13	131/24	149/47	140/17	149/80	71/57	46/127	133/26
	isolate 311	99/22	116/28	115/68	149/25	125/79	86/57	51/128	115/21
cdc73 tel1	RDKY8409	138/26	200/45	301/104	415/79	308/173	240/117	97/270	192/37
	isolate 321	180/34	244/62	283/99	367/60	331/176	182/112	75/235	231/42
	isolate 322	61/13	115/24	167/72	194/49	238/131	65/50	28/171	135/34
	isolate 323	155/26	253/61	274/81	320/57	327/165	157/89	73/222	262/36
	isolate 324	358/70	196/45	537/197	654/128	545/273	300/165	100/366	184/34
	isolate 325	213/40	302/84	329/131	356/74	335/181	193/126	79/280	262/38
	isolate 326	250/79	378/116	474/195	526/125	484/256	270/189	137/378	469/83
	isolate 327	114/26	187/37	262/99	217/49	233/141	138/95	70/203	154/45
	isolate 328	199/54	293/73	355/147	308/77	337/198	246/134	87/330	297/61
	isolate 329	122/24	154/30	206/80	260/50	251/150	111/63	47/176	197/35
	isolate 330	191/58	254/54	244/128	238/58	276/181	152/84	58/261	225/49
	isolate 331	81/18	126/34	161/80	160/32	186/121	69/59	27/145	113/22
cdc73 yku80	RDKY8411	120/29	190/54	192/86	191/83	152/144	129/69	65/206	179/31
	isolate 345	76/24	93/28	103/51	105/24	80/115	71/48	27/93	82/17
	isolate 346	105/19	128/31	132/60	124/21	112/86	97/63	35/143	103/25
	isolate 347	88/21	126/39	137/59	101/30	120/69	87/42	38/137	107/22
	isolate 348	399/70	508/114	445/166	483/137	398/226	386/159	109/358	472/102
	isolate 349	44/14	51/15	83/44	84/15	96/49	53/46	17/71	45/23
	isolate 350	411/63	483/107	342/157	456/124	317/236	341/160	80/299	407/78
	isolate 351	276/57	319/109	320/123	373/77	309/170	224/126	86/260	299/71
	isolate 352	259/52	344/76	372/153	361/74	279/159	256/145	74/267	331/70
	isolate 353	322/53	379/80	313/121	382/89	274/211	262/140	96/286	329/87
	isolate 354	283/65	424/127	379/137	439/93	371/224	329/162	98/336	388/69
	isolate 355	224/66	332/86	361/147	390/83	334/217	246/145	67/294	298/69
