# Supplementary material for: Cdc73 suppresses genome instability by mediating telomere homeostasis
Source: PLoS Genet. 2018 Jan 10;14(1):e1007170. doi: 10.1371/journal.pgen.1007170 (PMC5779705; doi:10.1371/journal.pgen.1007170)
Supplement: S9 Fig — a, c, e, g. Diagram of the HR event. b, d, f, h. Junction sequences and alignments between the GCR and the participating chromosomes identifies the novel junction sequences. Sequence of the junction between YCLWdelta5 (yellow) and other delta sequence (red) that fuse chromosome V (light magenta) with the other target (light grey). Sequence that could have been derived from either YCLWdelta5 or the other delta sequence is displayed with an orange background. (PDF) [file pgen.1007170.s009.pdf]

S9 Fig.

Isolate 302(*cdc73*), junction sequence obtained by linkage to chrV:34,339+ unique region

A.

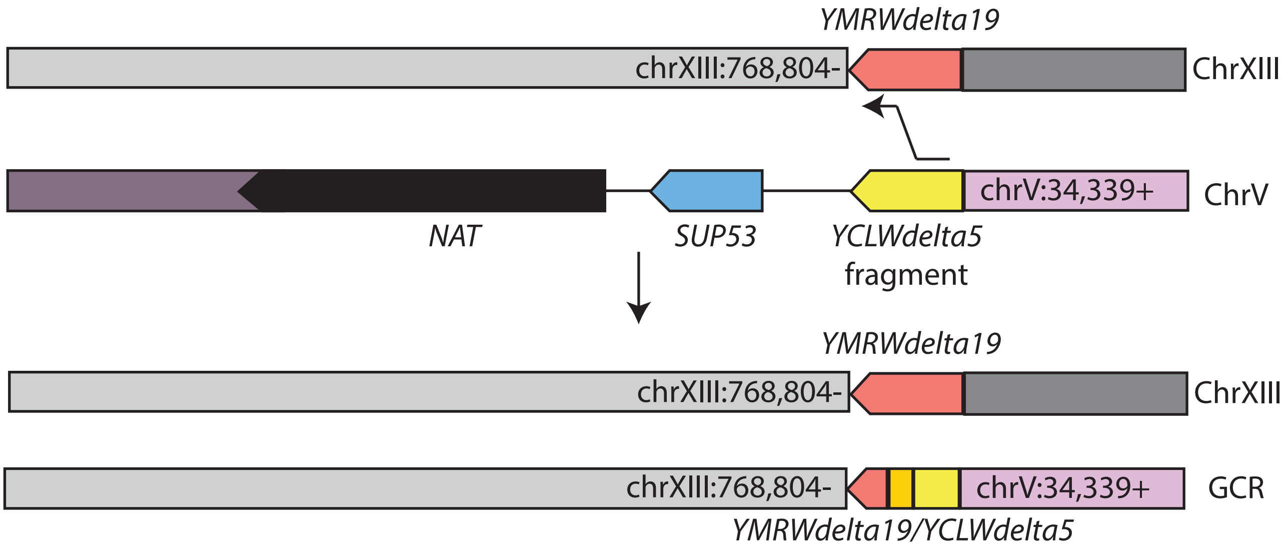

B.

```
ChrXIII:769209 AAATAGGTGACTTTTGAGATAGTTGTTGAATACCATTTCCATAACAATACAATTTGGACAAC
               ||||||||||||||||||||||||||||||||||||||||||||||||||||||||||||||
302            AAATAGGTGACTTTTGAGATAGTTGTTGAATACCATTTCCATAACAATACAATTTGGACAAC

ChrXIII:769147 ATTATCTTATACCGAATATACCTTAAGTTTTACTCGAAAATATAGGAATCCATAAAAGGATC
               ||||||||||||||||||||||||||||||||||||||||||||||||||||||||||||||
302            ATTATCTTATACCGAATATACCTTAAGTTTTACTCGAAAATATAGGAATCCATAAAAGGATC

ChrXIII:769085 GATGATTCTTCATAATAATATTATGGTTTCTCGTTCCACTTTATATGTTGTCACTCATTATC
               ||||||||||||||||||||||||||||||||||||||||||||||||||||||||||||||
302            GATGATTCTTCATAATAATATTATGGTTTCTCGTTCCACTTTATATGTTGTCACTCATTATC

ChrXIII:769023 CTATTGCATTATCAAGTCGTGCGTTCCACCCCCATTAAATTTGATGGCAGCTTTTAAATCTT
               ||||||||||||||||||||||||||||||||||||||||||||||||||||||||||||||
302            CTATTGCATTATCAAGTCGTGCGTTCCACCCCCATTAAATTTGATGGCAGCTTTTAAATCTT

ChrXIII:768961 TACCTTTATGCCATATTTCTACGTCGTATATGTAATAAGATGGATGCTAGACTATAGGGGGT
               ||||||||||||||||||||||||||||||||||||||||||||||||||||||||||||||
302            TACCTTTATGCCATATTTCTACGTCGTATATGTAATAAGATGGATGCTAGACTATAGGGGGT

ChrXIII:768899 AGGTGGTTCCTCTTCCGACATGT:AGAGAATGTGGATTTTGATGTAATTGTTGGGATTCCAT
               ||||||||||||||||||||||| ||||||||||||||||||||||||||||||||||||||
302            AGGTGGTTCCTCTTCCGACATGT:AGAGAATGTGGATTTTGATGTAATTGTTGGGATTCCAT
                                       ||||||||||||||||||||||||||||||||||||||
ChrIII:        aagtacaggacaattgattttga:AGAGAATGTGGATTTTGATGTAATTGTTGGGATTCCAT

ChrXIII:76837  T:GTGATTAAGGCTATAATATTAGGTATGTAGATATACTAGAAGTTCTCCTCGAGG:
               |  |   |||||| ||||||||||||||||||||||||||||||||||||||||||
302            T:TTTAATAAGGCAATAATATTAGGTATGTAGATATACTAGAAGTTCTCCTCGAGG:CTGGG
               | ||||||||||||||||||||||||||||||||||||||||||||||||||||||
ChrIII:        T:TTTAATAAGGCAATAATATTAGGTATGTAGATATACTAGAAGTTCTCCTCGAGG:
                                                                        |||||
ChrV:34339                                                             :CTGGG
```

S9 Fig.

Isolate 544 (wild-type), junction sequence obtained by linkage to chrV:34,339+ unique region

C.

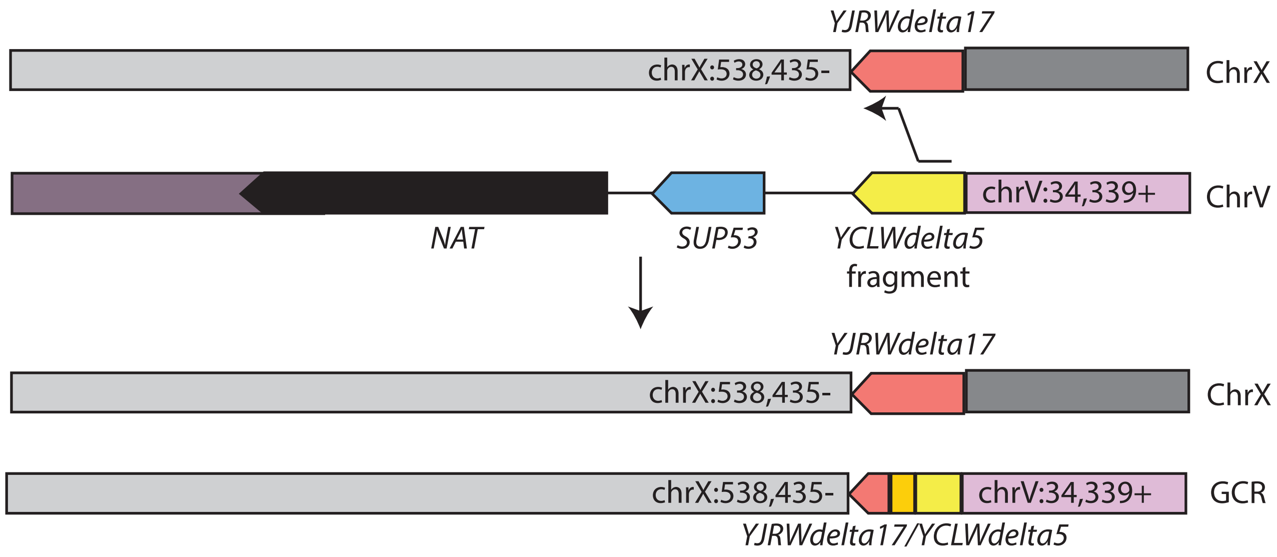

D.

```
ChrX:538534   AAATATGAGGTACCTAAGTTGCATAGTAATATTATTACGAAGCACTTCAAATTGAATGAATAAAC
              |||||||||||||||||||||||||||||||||||||||||||||||||||||||||||||||||
Junction      AAATATGAGGTACCTAAGTTGCATAGTAATATTATTACGAAGCACTTCAAATTGAATGAATAAAC


ChrX:538469   TTCGTGCAGAAAACTTACACGATGGAAATCAATA:TTGTAGAGAATGTGGATTTTGATGTAATTG
              |||||||||||||||||||||||||||||||||| ||||||||||||||||||||||||||||||
Junction      TTCGTGCAGAAAACTTACACGATGGAAATCAATA:TTGTAGAGAATGTGGATTTTGATGTAATTG
                                   |    |||  ||  ||||||||||||||||||||||||||||||
ChrIII:90784                      caggacaattgatt:TTGAAGAGAATGTGGATTTTGATGTAATTG


ChrX:538405   TTGGGATTCCATTTTTAATAAGGCAATAATATTAGGTATGTAGATATACTAGAAGTTCTCCTCGA
              |||||||||||||||||||||||||||||||||||||||||||||||||||||||||||||||||
Junction      TTGGGATTCCATTTTTAATAAGGCAATAATATTAGGTATGTAGATATACTAGAAGTTCTCCTCGA
              |||||||||||||||||||||||||||||||||||||||||||||||||||||||||||||||||
ChrIII:90736  TTGGGATTCCATTTTTAATAAGGCAATAATATTAGGTATGTAGATATACTAGAAGTTCTCCTCGA


ChrX:538339   GG
              ||
Junction      GG:CTGGGTTAGCTTGAAGCGACTTTCTTTCTCTACTAAAGGGAATGGTCAGATCATCAGGCCAA
              ||
ChrIII:90675  GG
                 ||||||||||||||||||||||||||||||||||||||||||||||||||||||||||||||
ChrV:34339      :CTGGGTTAGCTTGAAGCGACTTTCTTTCTCTACTAAAGGGAATGGTCAGATCATCAGGCCAA
```

S9 Fig.

Isolate 550 (wild-type), junction sequence obtained by linkage to chrV:34,339+ unique region

E.

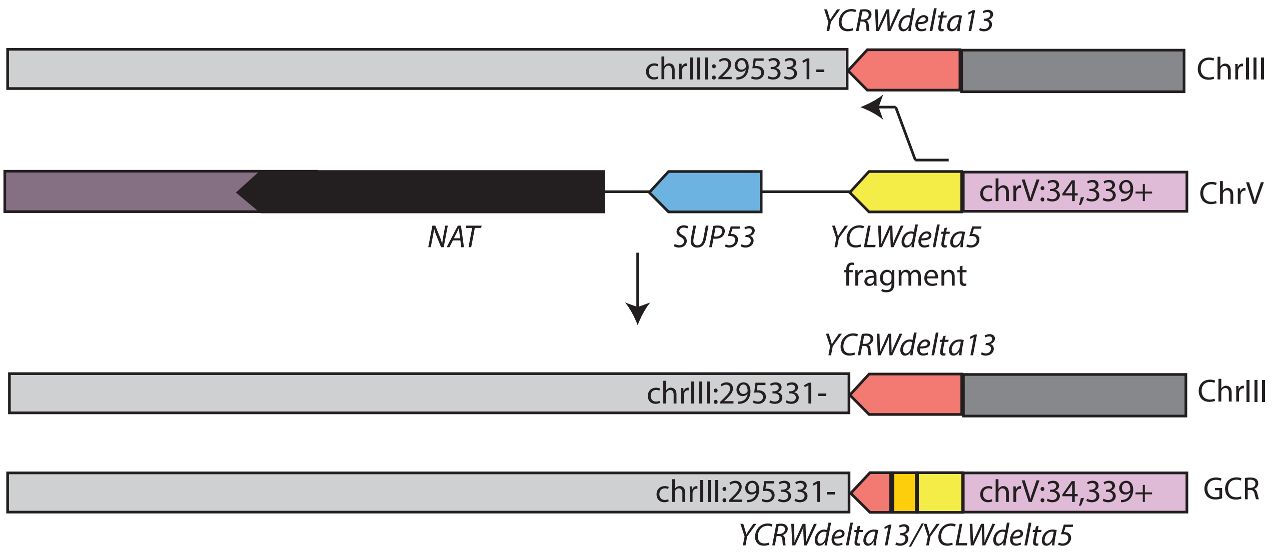

F.

```
ChrIII:295435 AAATAAAATTGGTAGAATGACCTAGAATGACCCATCCGCCGCGGAATCGACGTCGGATTTG
              |||||||||||||||||||||||||||||||||||||||||||||||||||||||||||||
Junction      AAATAAAATTGGTAGAATGACCTAGAATGACCCATCCGCCGCGGAATCGACGTCGGATTTG

ChrIII:295374 CGCTTGACAATTCTATATGCAAATTTAGGAAAGTAATCTGCAGATGCGGAATTGGTGAATT
              |||||||||||||||||||||||||||||||||||||||||||||||||||||||||||||
Junction      CGCTTGACAATTCTATATGCAAATTTAGGAAAGTAATCTGCAGATGCGGAATTGGTGAATT

ChrIII:295313 TTTATATTATTGTGGGGTTGCATTGTAGATAAAAGT:AATAATATTAGGTAT:atagaata
              |||||||||||||||||||||||||||||||||||| |||||||||||||||
Junction      TTTATATTATTGTGGGGTTGCATTGTAGATAAAAGT:AATAATATTAGGTAT:GTAGATATA
                                             ||    ||||||||||||||| |||||||||
ChrIII:90721                                 aaggc:AATAATATTAGGTAT:GTAGATATA

ChrV:34339                       CTGGGTTAGCTTGAAGCGACTTTCTTTCTCTACTAAAGGGA
                                 |||||||||||||||||||||||||||||||||||||||||
Junction      CTAGAAGTTCTCCTCGAGGCTGGGTTAGCTTGAAGCGACTTTCTTTCTCTACTAAAGGGA
              |||||||||||||||||||
ChrIII:90693  CTAGAAGTTCTCCTCGAGG
```

S9 Fig.

Isolate 354 (*cdc73Δ yku80Δ*), junction sequence obtained by linkage to chrV:34,339+ unique region

G.

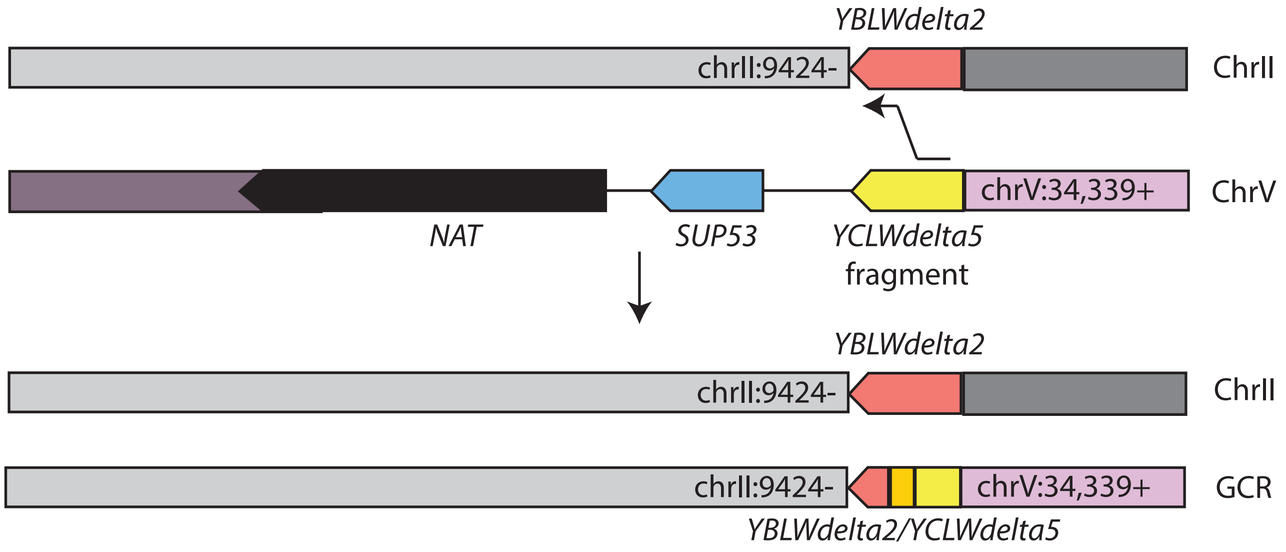

H.

```
ChrV   34398 GGCCTGATGATCTGACCATTCCCTTTAGTAGAGAAAGAAAGTCGCTTCAAGCTAACCCAG
             ||||||||||||||||||||||||||||||||||||||||||||||||||||||||||||
Junction     GGCCTGATGATCTGACCATTCCCTTTAGTAGAGAAAGAAAGTCGCTTCAAGCTAACCCAG

ChrIII 90672 CCTCGAGGAGAACTTCTAGTATATCTACATACCTAATATTATTGCCTTATTAAA:AATG
             |||||||||||||||||||||||||||||||||||||||||||||||||||||| ||||
Junction     CCTCGAGGAGAACTTCTAGTATATCTACATACCTAATATTATTGCCTTATTAAA:AATG
             ||| |||||||||||||||||||||||||||||||||||||| |||||| | |  ||||
ChrII  9327  CCTGGAGGAGAACTTCTAGTATATCTACATACCTAATATTATAGCCTTAATCAC:AATG

Sbjct: 90732 GAATCCCAACAATTACATCAAAATCCACATTCTCT:TCA
             |||||||||||||||||||||||||||||||||||  ||
Junction     GAATCCCAACAATTACATCAAAATCCACATTCTCT:ACACCAATACCATCGACGAGAGCTT
             ||||||||||||||||||||||||||||||||||| |||||||||||||||||||||||||
ChrII  9387  GAATCCCAACAATTACATCAAAATCCACATTCTCT:ACACCAATACCATCGACGAGAGCTT

Junction     CTAGTAAATTGTATACATAACAGTATAACCCTTACCAACAATGGAATCTCAAAGATTATT
             ||||||||||||||||||||||||||||||||||||||||||||||||||||||||||||
ChrII  9447  CTAGTAAATTGTATACATAACAGTATAACCCTTACCAACAATGGAATCTCAAAGATTATT

Junction     AAATTATTCACAGACTCTGAGGATTCGGGTAAAATAGGGTATTTAACTGGTTACCGGAAA
             ||||||||||||||||||||||||||||||||||||||||||||||||||||||||||||
ChrII  9507  AAATTATTCACAGACTCTGAGGATTCGGGTAAAATAGGGTATTTAACTGGTTACCGGAAA

Junction     GGTTTAGAAAATTCGTGGAGGGTTGGCCGAGTGGTCTAAGGCGGCAGACTTAAGATCTGT
             ||||||||||||||||||||||||||||||||||||||||||||||||||||||||||||
ChrII  9567  GGTTTAGAAAATTCGTGGAGGGTTGGCCGAGTGGTCTAAGGCGGCAGACTTAAGATCTGT

Junction     TGGACGGTTGTCCGCGCGAGTTCGAACCTCGCATCCTTCAGTATTTTTTTTGATGATTTA
             ||||||||||||||||||||||||||||||||||||||||||||||||||||||||||||
ChrII  9627  TGGACGGTTGTCCGCGCGAGTTCGAACCTCGCATCCTTCAGTATTTTTTTTGATGATTTA

Junction     ACGTACTATTAACTAGAATAATAGGGAAATGCAATTGCAGTTTGAG
             ||||||||||||||||||||||||||||||||||||||||||||||
ChrII  9687  ACGTACTATTAACTAGAATAATAGGGAAATGCAATTGCAGTTTGAG
```
