# Supplementary material for: Cdc73 suppresses genome instability by mediating telomere homeostasis
Source: PLoS Genet. 2018 Jan 10;14(1):e1007170. doi: 10.1371/journal.pgen.1007170 (PMC5779705; doi:10.1371/journal.pgen.1007170)
Supplement: S10 Fig — a, c. Diagram of the HR event. b, d. Junction sequences and alignments between the GCR and the participating chromosomes identifies the novel junction sequences. Sequence of the junction between SUP53 (blue) and other tRNA gene (green) that fuses chromosome V (light magenta) with the other target (light grey). Sequence that could have been derived from either SUP53 or the other tRNA is displayed with a cyan background. (PDF) [file pgen.1007170.s010.pdf]

S10 Fig.

Isolate 542 (wild-type), junction sequence obtained by linkage to chrV:34,339+ unique region
Isolate 309 (cdc73), junction sequence obtained by linkage to chrV:34,339+ unique region

A.

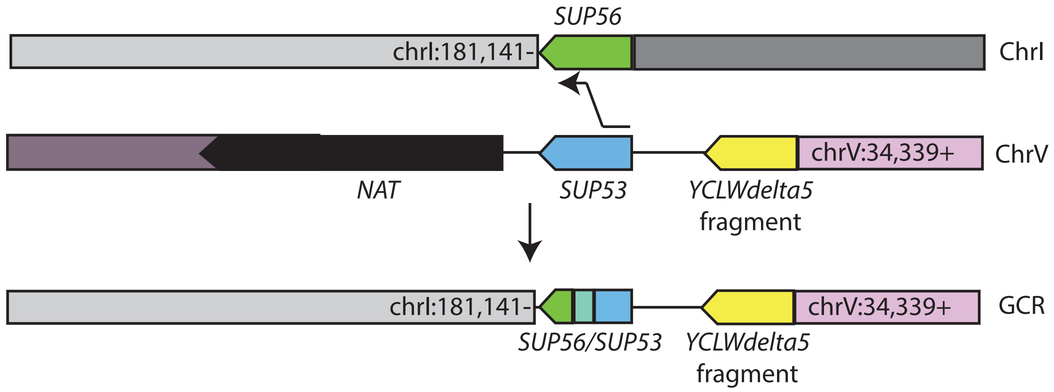

B.

```
ChrI:181341   ATCCACGAAACACACCCTCATTGGTATAATGTTGGTACTTTCATTCCTACGTAGATTATGAAGTA
              |||||||||||||||||||||||||||||||||||||||||||||||||||||||||||||||||
309           ATCCACGAAACACACCCTCATTGGTATAATGTTGGTACTTTCATTCCTACGTAGATTATGAAGTA
542           ATCCACGAAACACACCCTCATTGGTATAATGTTGGTACTTTCATTCCTACGTAGATTATGAAGTA

ChrI:181276   TAGGAGGAAAAAG:AAAAAATAATGGTTGCTAAGAGATTCGAACTCTTGCATCTTACGATACCTG
              ||||||||||||| |||||||||||||||||||||||||||||||||||||||||||||||||||
309           TAGGAGGAAAAAG:AAAAAATAATGGTTGCTAAGAGATTCGAACTCTTGCATCTTACGATACCTG
542           TAGGAGGAAAAAG:AAAAAATAATGGTTGCTAAGAGATTCGAACTCTTGCATCTTACGATACCTG
                   |   |    |||||||||||||||||||||||||||||||||||||||||||||||||||
ChrIII:90991  gttatgttgagga:AAAAAATAATGGTTGCTAAGAGATTCGAACTCTTGCATCTTACGATACCTG

ChrI:181211   AGTATTCCCACAGTT:cactgcggtcaagatatttcttgaatcaggcgccttagaccgctcggcc
              |||||||||||||||  ||||||||||||||||||||||||||||||||||||||||||||||||
309           AGTATTCCCACAGTT:AACTGCGGTCAAGATATTTCTTGAATCAGGCGCCTTAGACCGCTCGGCC
542           AGTATTCCCACAGTT:AACTGCGGTCAAGATATTTCTTGAATCAGGCGCCTTAGACCGCTCGGCC
              ||||||||||||||| |||||||||||||||||||||||||||||||||||||||||||||||||
ChrIII:90930  AGTATTCCCACAGTT:AACTGCGGTCAAGATATTTCTTGAATCAGGCGCCTTAGACCGCTCGGCC

ChrI:181146   aaacaaccacttatttgt
              |||||||| ||| ||||
309           AAACAACCAATTACTTGTTGAGAAATAGAGTATAATTATCCTATAAATATAACGTTTTTGAACAC
542           AAACAACCAATTACTTGTTGAGAAATAGAGTATAATTATCCTATAAATATAACGTTTTTGAACAC
              |||||||||||||||||||||||||||||||||||||||||||||||||||||||||||||||||
ChrIII:90866  AAACAACCAATTACTTGTTGAGAAATAGAGTATAATTATCCTATAAATATAACGTTTTTGAACAC


309           ACATGAACAAGGAAGTACAGGACAATTGATTTTGAAGAGAATGTGGATTTTGATGTAATTGTTGG
542           ACATGAACAAGGAAGTACAGGACAATTGATTTTGAAGAGAATGTGGATTTTGATGTAATTGTTGG
              |||||||||||||||||||||||||||||||||||||||||||||||||||||||||||||||||
ChrIII:90800  ACATGAACAAGGAAGTACAGGACAATTGATTTTGAAGAGAATGTGGATTTTGATGTAATTGTTGG

ChrV:34339                                                                   C
                                                                             |
309           GATTCCATTTTTAATAAGGCAATAATATTAGGTATGTAGATATACTAGAAGTTCTCCTCGAGGC
542           GATTCCATTTTTAATAAGGCAATAATATTAGGTATGTAGATATACTAGAAGTTCTCCTCGAGGC
              ||||||||||||||||||||||||||||||||||||||||||||||||||||||||||||||||
ChrIII:90734  GATTCCATTTTTAATAAGGCAATAATATTAGGTATGTAGATATACTAGAAGTTCTCCTCGAGG

ChrV:34340    TGGGTTAGCTTGAAGCGACTTTC
              |||||||||||||||||||||||
309           TGGGTTAGCTTGAAGCGACTTTC
542           TGGGTTAGCTTGAAGCGACTTTC
```

S10 Fig.

Isolate 547 (wild-type), junction sequence obtained by linkage to chrV:34,339+ unique region

C.

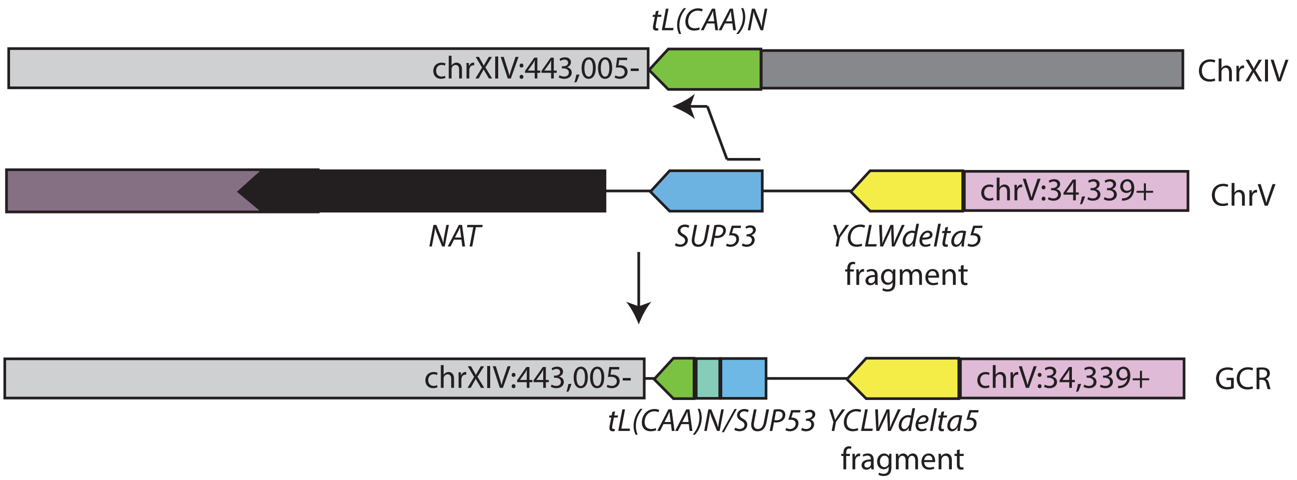

D.

```
ChrXIV:443186  ACATTTTGGAATACATATTGGGTTGATTTCCCAAGTTCTCGCTGCAATCAGATAAAAAGGA:AAAA
               ||||||||||||||||||||||||||||||||||||||||||||||||||||||||||||| ||||
Junction       ACATTTTGGAATACATATTGGGTTGATTTCCCAAGTTCTCGCTGCAATCAGATAAAAAGGA:AAAA
                                                                             ||||
ChrIII:90991                                                     gttatgttgagg:AAAA

ChrXIV:443125  AAATAATGGTTGCTAAGAGATTCGAACTCTTGCATCTTACGATACCTGAGTATTCCCACAGTTAA
               |||||||||||||||||||||||||||||||||||||||||||||||||||||||||||||||||
Junction       AAATAATGGTTGCTAAGAGATTCGAACTCTTGCATCTTACGATACCTGAGTATTCCCACAGTTAA
               |||||||||||||||||||||||||||||||||||||||||||||||||||||||||||||||||
ChrIII:90978   AAATAATGGTTGCTAAGAGATTCGAACTCTTGCATCTTACGATACCTGAGTATTCCCACAGTTAA

ChrXIV:443060  CTGCGGTCAAGATATTTCTTGAATCAGGCGCCTTAGACCGCTCGGCCAAACAACCA:cttatttg
               ||||||||||||||||||||||||||||||||||||||||||||||||||||||||  ||| |||
Junction       CTGCGGTCAAGATATTTCTTGAATCAGGCGCCTTAGACCGCTCGGCCAAACAACCA:ATTACTTG
               |||||||||||||||||||||||||||||||||||||||||||||||||||||||| ||||||||
ChrIII:90913   CTGCGGTCAAGATATTTCTTGAATCAGGCGCCTTAGACCGCTCGGCCAAACAACCA:ATTACTTG


Junction       TTGAGAAATAGAGTATAATTATCCTATAAATATAACGTTTTTGAACACACATGAACAAGGAAGTA
               |||||||||||||||||||||||||||||||||||||||||||||||||||||||||||||||||
ChrIII:90849   TTGAGAAATAGAGTATAATTATCCTATAAATATAACGTTTTTGAACACACATGAACAAGGAAGTA


Junction       CAGGACAATTGATTTTGAAGAGAATGTGGATTTTGATGTAATTGTTGGGATTCCATTTTTAATAA
               |||||||||||||||||||||||||||||||||||||||||||||||||||||||||||||||||
ChrIII:90784   CAGGACAATTGATTTTGAAGAGAATGTGGATTTTGATGTAATTGTTGGGATTCCATTTTTAATAA

ChrV:34339                                                    CTGGGTTAGCTTGAAGCGA
                                                              |||||||||||||||||||
Junction       GGCAATAATATTAGGTATGTAGATATACTAGAAGTTCTCCTCGAGGCTGGGTTAGCTTGAAGCGA
               ||||||||||||||||||||||||||||||||||||||||||||||
ChrIII:90719   GGCAATAATATTAGGTATGTAGATATACTAGAAGTTCTCCTCGAGG

ChrV:34358     CTTTCTTTCTCTACTAAAGGGAATGGTCA
               |||||||||||||||||||||||||||||
Junction       CTTTCTTTCTCTACTAAAGGGAATGGTCA
```
