# Supplementary material for: Cdc73 suppresses genome instability by mediating telomere homeostasis
Source: PLoS Genet. 2018 Jan 10;14(1):e1007170. doi: 10.1371/journal.pgen.1007170 (PMC5779705; doi:10.1371/journal.pgen.1007170)
Supplement: S11 Fig — a. Diagram of the HR event. b. Junction sequences displayed as in S9 Fig. (PDF) [file pgen.1007170.s011.pdf]

S11 Fig.

A. ***YCLWdelta5/YELWdelta1*, junction sequences obtained by linkage to chrV:34,339+ unique region**

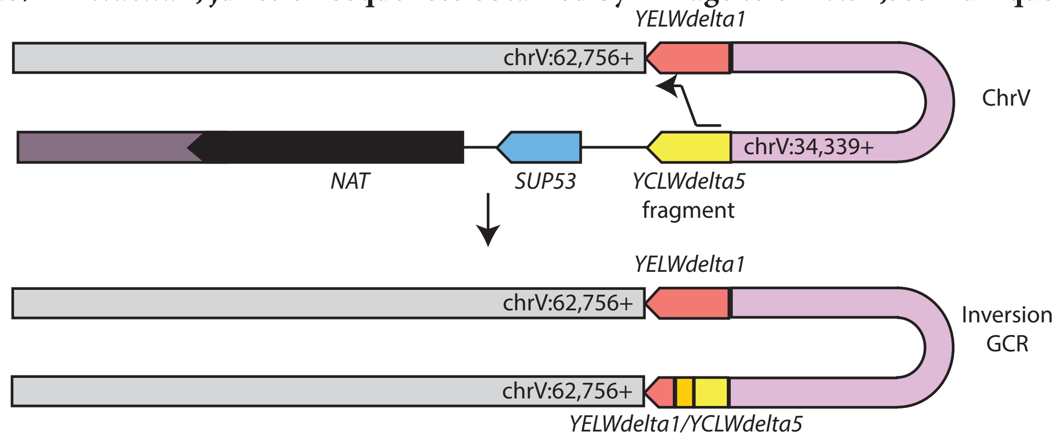

B.

```
                                   ChrV 34,339-:
parental GAGAAAGAAAGTCGCTTCAAGCTAACCCAG:CCTCGAGGAGAACTTCTAGTA:TA-:TCTACATACCTAATATTATTGCCTTATTAAAAATGGAA
         ||||||||||||||||||||||||||||||  ||||||||||||||||||||| :|  ||||||||||||||||||||||||||||||||||||||||
YELWΔ1                                 :CCTCGAGGAGAACTTCTAGTA:CAT:TCTACATACCTAATATTATTGCCTTATTAAAAATGGAA
                                        ||||||||||||||||||||| :|: ||||||||||||||||||||||||||||||||||||||||
352      GAGAAAGAAAGTCGCTTCAAGCTAACCCAG:CCTCGAGGAGAACTTCTAGTA:CAT:TCTACATACCTAATATTATTGCCTTATTAAAAATGGAA
350      GAGAAAGAAAGTCGCTTCAAGCTAACCCAG:CCTCGAGGAGAACTTCTAGTA:CAT:TCTACATACCTAATATTATTGCCTTATTAAAAATGGAA
324      GAGAAAGAAAGTCGCTTCAAGCTAACCCAG:CCTCGAGGAGAACTTCTAGTA:CAT:TCTACATACCTAATATTATTGCCTTATTAAAAATGGAA
304      GAGAAAGAAAGTCGCTTCAAGCTAACCCAG:CCTCGAGGAGAACTTCTAGTA:CAT:TCTACATACCTAATATTATTGCCTTATTAAAAATGGAA
310      GAGAAAGAAAGTCGCTTCAAGCTAACCCAG:CCTCGAGGAGAACTTCTAGTA:CAT:TCTACATACCTAATATTATTGCCTTATTAAAAATGGAA
328      GAGAAAGAAAGTCGCTTCAAGCTAACCCAG:CCTCGAGGAGAACTTCTAGTA:CAT:TCTACATACCTAATATTATTGCCTTATTAAAAATGGAA
331      GAGAAAGAAAGTCGCTTCAAGCTAACCCAG:CCTCGAGGAGAACTTCTAGTA:CAT:TCTACATACCTAATATTATTGCCTTATTAAAAATGGAA
353      GAGAAAGAAAGTCGCTTCAAGCTAACCCAG:CCTCGAGGAGAACTTCTAGTA:CAT:TCTACATACCTAATATTATTGCCTTATTAAAAATGGAA
548      GAGAAAGAAAGTCGCTTCAAGCTAACCCAG:CCTCGAGGAGAACTTCTAGTA:CAT:TCTACATACCTAATATTATTGCCTTATTAAAAATGGAA
346      GAGAAAGAAAGTCGCTTCAAGCTAACCCAG:CCTCGAGGAGAACTTCTAGTA:TA-:TCTACATACCTAATATTATTGCCTTATTAAAAATGGAA
330      GAGAAAGAAAGTCGCTTCAAGCTAACCCAG:CCTCGAGGAGAACTTCTAGTA:TA-:TCTACATACCTAATATTATTGCCTTATTAAAAATGGAA
326      GAGAAAGAAAGTCGCTTCAAGCTAACCCAG:CCTCGAGGAGAACTTCTAGTA:TA-:TCTACATACCTAATATTATTGCCTTATTAAAAATGGAA
325      GAGAAAGAAAGTCGCTTCAAGCTAACCCAG:CCTCGAGGAGAACTTCTAGTA:TA-:TCTACATACCTAATATTATTGCCTTATTAAAAATGGAA
308      GAGAAAGAAAGTCGCTTCAAGCTAACCCAG:CCTCGAGGAGAACTTCTAGTA:TA-:TCTACATACCTAATATTATTGCCTTATTAAAAATGGAA
307      GAGAAAGAAAGTCGCTTCAAGCTAACCCAG:CCTCGAGGAGAACTTCTAGTA:TA-:TCTACATACCTAATATTATTGCCTTATTAAAAATGGAA
305      GAGAAAGAAAGTCGCTTCAAGCTAACCCAG:CCTCGAGGAGAACTTCTAGTA:TA-:TCTACATACCTAATATTATTGCCTTATTAAAAATGGAA
306      GAGAAAGAAAGTCGCTTCAAGCTAACCCAG:CCTCGAGGAGAACTTCTAGTA:TA-:TCTACATACCTAATATTATTGCCTTATTAAAAATGGAA
311      GAGAAAGAAAGTCGCTTCAAGCTAACCCAG:CCTCGAGGAGAACTTCTAGTA:TA-:TCTACATACCTAATATTATTGCCTTATTAAAAATGGAA
351      GAGAAAGAAAGTCGCTTCAAGCTAACCCAG:CCTCGAGGAGAACTTCTAGTA:TA-:TCTACATACCTAATATTATTGCCTTATTAAAAATGGAA
355      GAGAAAGAAAGTCGCTTCAAGCTAACCCAG:CCTCGAGGAGAACTTCTAGTA:TA-:TCTACATACCTAATATTATTGCCTTATTAAAAATGGAA
347      GAGAAAGAAAGTCGCTTCAAGCTAACCCAG:CCTCGAGGAGAACTTCTAGTA:TA-:TCTACATACCTAATATTATTGCCTTATTAAAAATGGAA
323      GAGAAAGAAAGTCGCTTCAAGCTAACCCAG:CCTCGAGGAGAACTTCTAGTA:TA-:TCTACATACCTAATATTATTGCCTTATTAAAAATGGAA
345      GAGAAAGAAAGTCGCTTCAAGCTAACCCAG:CCTCGAGGAGAACTTCTAGTA:TA-:TCTACATACCTAATATTATTGCCTTATTAAAAATGGAA

parental TCCCAACAATTA:CA:TCAAAAT:C:CAC:ATT:CTCTTCAA:aatcaattgtcctgtacttccttgttcatgtgtgttcaaaaacgttatattt
         |||||||||||| :: ||||||| : ||| ::: ||||||||
YELWΔ1   TCCCAACAATTA:TC:TCAAAAT:T:CAC:CCA:CTCTTCAA:TTATCATATAGGACAGAATTAGTTGACAAAAAGTACGATGGGAAACAGCTAT
         |||||||||||| :: ||||||| : ||| ::: |||||||| ||||||||||||||||||||||||||||||||||||||||||||||||||||
352      TCCCAACAATTA:TC:TCAAAAT:C:CAC:CCA:CTCTTCAA:TTATCATATAGGACAGAATTAGTTGACAAAAAGTACGATGGGAAACAGCTAT
350      TCCCAACAATTA:TC:TCAAAAT:T:CAC:CCA:CTCTTCAA:TTATCATATAGGACAGAATTAGTTGACAAAAAGTACGATGGGAAACAGCTAT
324      TCCCAACAATTA:TC:TCAAAAT:T:CAC:CCA:CTCTTCAA:TTATCATATAGGACAGAATTAGTTGACAAAAAGTACGATGGGAAACAGCTAT
304      TCCCAACAATTA:TC:TCAAAAT:T:CAC:CCA:CTCTTCAA:TTATCATATAGGACAGAATTAGTTGACAAAAAGTACGATGGGAAACAGCTAT
310      TCCCAACAATTA:TC:TCAAAAT:T:CAC:CCA:CTCTTCAA:TTATCATATAGGACAGAATTAGTTGACAAAAAGTACGATGGGAAACAGCTAT
328      TCCCAACAATTA:TC:TCAAAAT:T:CAC:CCA:CTCTTCAA:TTATCATATAGGACAGAATTAGTTGACAAAAAGTACGATGGGAAACAGCTAT
331      TCCCAACAATTA:TC:TCAAAAT:T:CAC:CCA:CTCTTCAA:TTATCATATAGGACAGAATTAGTTGACAAAAAGTACGATGGGAAACAGCTAT
353      TCCCAACAATTA:TC:TCAAAAT:T:CAC:CCA:CTCTTCAA:TTATCATATAGGACAGAATTAGTTGACAAAAAGTACGATGGGAAACAGCTAT
548      TCCCAACAATTA:TC:TCAAAAT:T:CAC:CCA:CTCTTCAA:TTATCATATAGGACAGAATTAGTTGACAAAAAGTACGATGGGAAACAGCTAT
346      TCCCAACAATTA:TC:TCAAAAT:T:CAC:CCA:CTCTTCAA:TTATCATATAGGACAGAATTAGTTGACAAAAAGTACGATGGGAAACAGCTAT
330      TCCCAACAATTA:TC:TCAAAAT:T:CAC:CCA:CTCTTCAA:TTATCATATAGGACAGAATTAGTTGACAAAAAGTACGATGGGAAACAGCTAT
326      TCCCAACAATTA:TC:TCAAAAT:T:CAC:CCA:CTCTTCAA:TTATCATATAGGACAGAATTAGTTGACAAAAAGTACGATGGGAAACAGCTAT
325      TCCCAACAATTA:TC:TCAAAAT:T:CAC:CCA:CTCTTCAA:TTATCATATAGGACAGAATTAGTTGACAAAAAGTACGATGGGAAACAGCTAT
308      TCCCAACAATTA:TC:TCAAAAT:T:CAC:CCA:CTCTTCAA:TTATCATATAGGACAGAATTAGTTGACAAAAAGTACGATGGGAAACAGCTAT
307      TCCCAACAATTA:TC:TCAAAAT:T:CAC:CCA:CTCTTCAA:TTATCATATAGGACAGAATTAGTTGACAAAAAGTACGATGGGAAACAGCTAT
305      TCCCAACAATTA:TC:TCAAAAT:T:CAC:CCA:CTCTTCAA:TTATCATATAGGACAGAATTAGTTGACAAAAAGTACGATGGGAAACAGCTAT
306      TCCCAACAATTA:TC:TCAAAAT:T:CAC:CCA:CTCTTCAA:TTATCATATAGGACAGAATTAGTTGACAAAAAGTACGATGGGAAACAGCTAT
311      TCCCAACAATTA:TC:TCAAAAT:T:CAC:CCA:CTCTTCAA:TTATCATATAGGACAGAATTAGTTGACAAAAAGTACGATGGGAAACAGCTAT
351      TCCCAACAATTA:TC:TCAAAAT:T:CAC:CCA:CTCTTCAA:TTATCATATAGGACAGAATTAGTTGACAAAAAGTACGATGGGAAACAGCTAT
355      TCCCAACAATTA:TC:TCAAAAT:T:CAC:CCA:CTCTTCAA:TTATCATATAGGACAGAATTAGTTGACAAAAAGTACGATGGGAAACAGCTAT
347      TCCCAACAATTA:CA:TCAAAAT:T:CAC:CCA:CTCTTCAA:TTATCATATAGGACAGAATTAGTTGACAAAAAGTACGATGGGAAACAGCTAT
323      TCCCAACAATTA:CA:TCAAAAT:C:CAC:ATT:CTCTTCAA:TTATCATATAGGACAGAATTAGTTGACAAAAAGTACGATGGGAAACAGCTAT
345      TCCCAACAATTA:CA:TCAAAAT:C:CAC:ATT:CTCTTCAA:TTATCATATAGGACAGAATTAGTTGACAAAAAGTACGATGGGAAACAGCTAT
```
