# Supplementary material for: Cdc73 suppresses genome instability by mediating telomere homeostasis
Source: PLoS Genet. 2018 Jan 10;14(1):e1007170. doi: 10.1371/journal.pgen.1007170 (PMC5779705; doi:10.1371/journal.pgen.1007170)

S12 Fig.

A. **Isolate 303 (*cdc73*)**

```
                                         KX377639.1 (nat) 1,213+:
GCGCGTTGATGGGGCTCGCGACGGAGTTCGCCCGCGAGCGGGGCG:CCGGG:
GCGCGTTGATGGGGCTCGCGACGGAGTTCGCCCGCGAGCGGGGCG:CCGGG:AGTCCGGGTCGCCGTCCTCCCCGTCGTCCGATTCG
                                             :CCGGG:AGTCCGGGTCGCCGTCCTCCCCGTCGTCCGATTCG
                                             :KX377639.1 (nat) 1,033-
```

B. **Potential inversion formation mechanism**

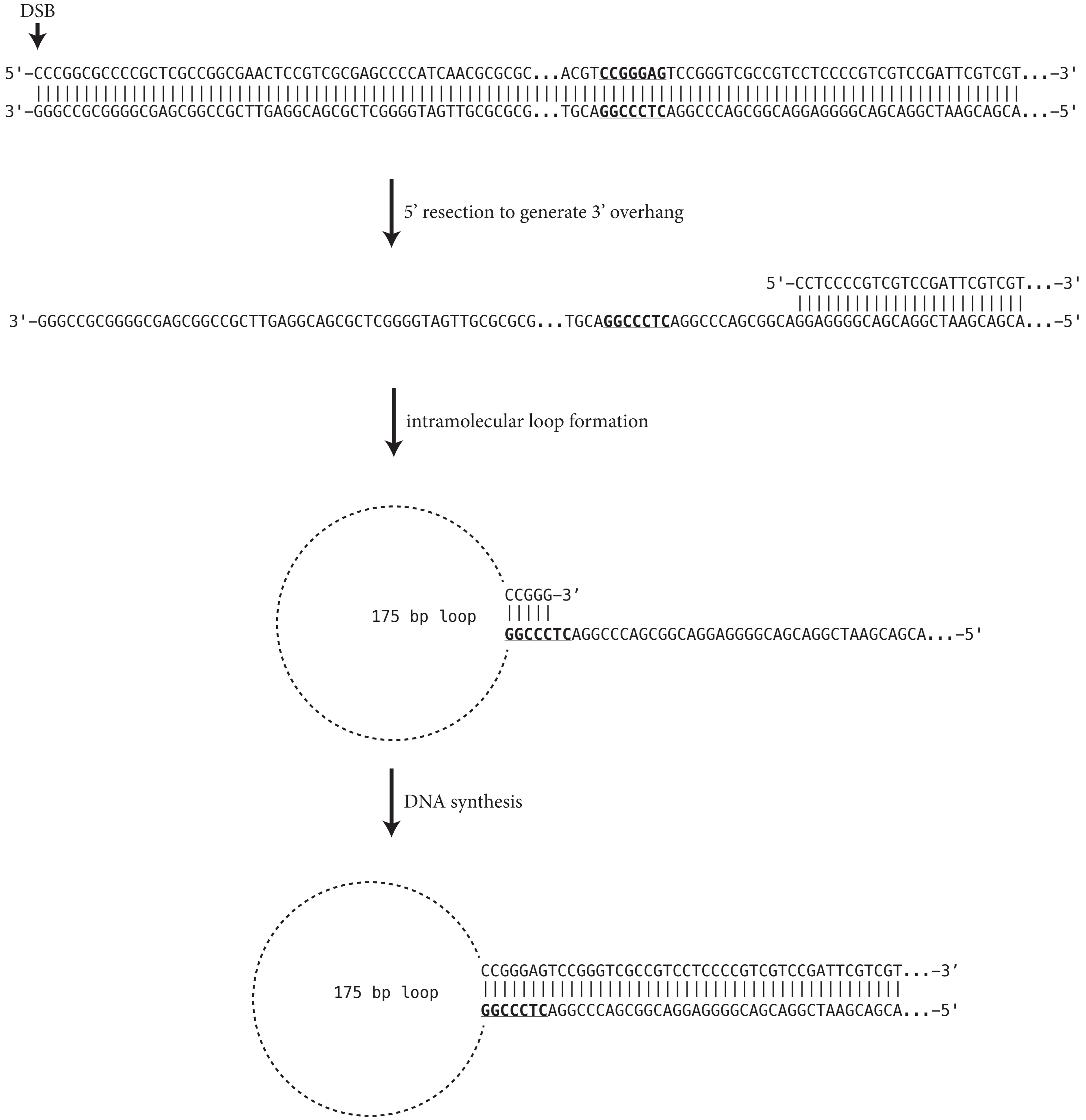

S12 Fig.

C. Isolate 321 (*tel1 cdc73*)

Mapped junction sequence (note that *CAN1* region is in the opposite orientation in sGCR strains)

```
                                        ChrV 32544+:
ATGGCTCTTGGAACGGATTTTCTGGGGTTTGCAG:CTTCAC:
ATGGCTCTTGGAACGGATTTTCTGGGGTTTGCAG:CTTCAC:ATTTCAAGGTACTGAACTAGTTGGTATCACTGCTGGTG
                                  :CTTCAC:ATTTCAAGGTACTGAACTAGTTGGTATCACTGCTGGTG
                                  :ChrV 32,585-
```

D. Potential inversion formation mechanism

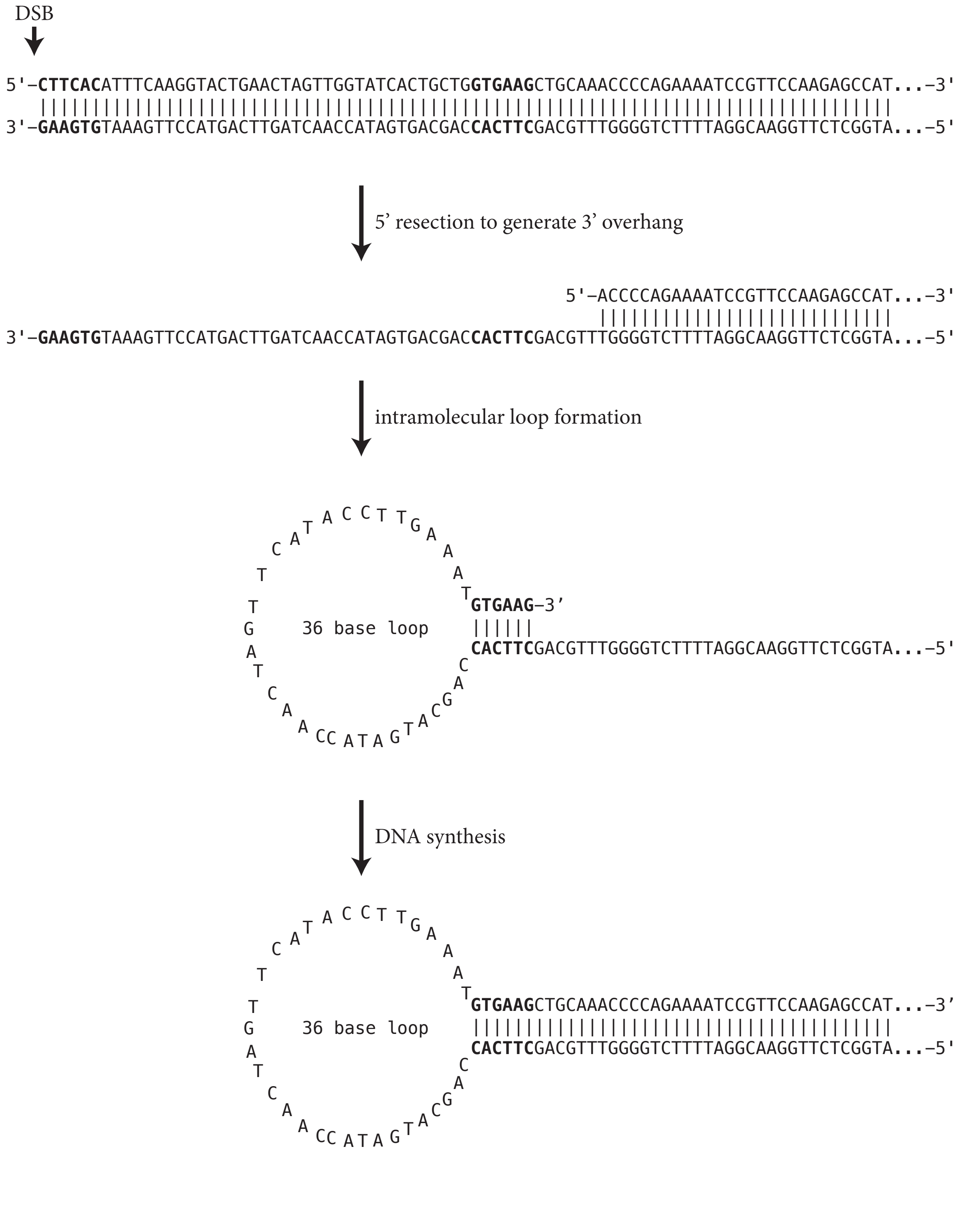

S12 Fig.

E. **Isolate 322 (*tel1 cdc73*)**

Mapped junction sequence

```
                                           ChrV 44,727-:
CTATCTTGTCTTGCAACCCCACTGGATTGAGCAATTTTCTT:TCCTGG:
CTATCTTGTCTTGCAACCCCACTGGATTGAGCAATTTTCTT:TCCTGG:CCCAATTCGAAAAATTCACTATCAGAACCTGGTTGCA
                                                 :TCCTGG:CCCAATTCGAAAAATTCACTATCAGAACCTGGTTGCA
                                                 :ChrV 40,308+
```

F. **Potential inversion formation mechanism**

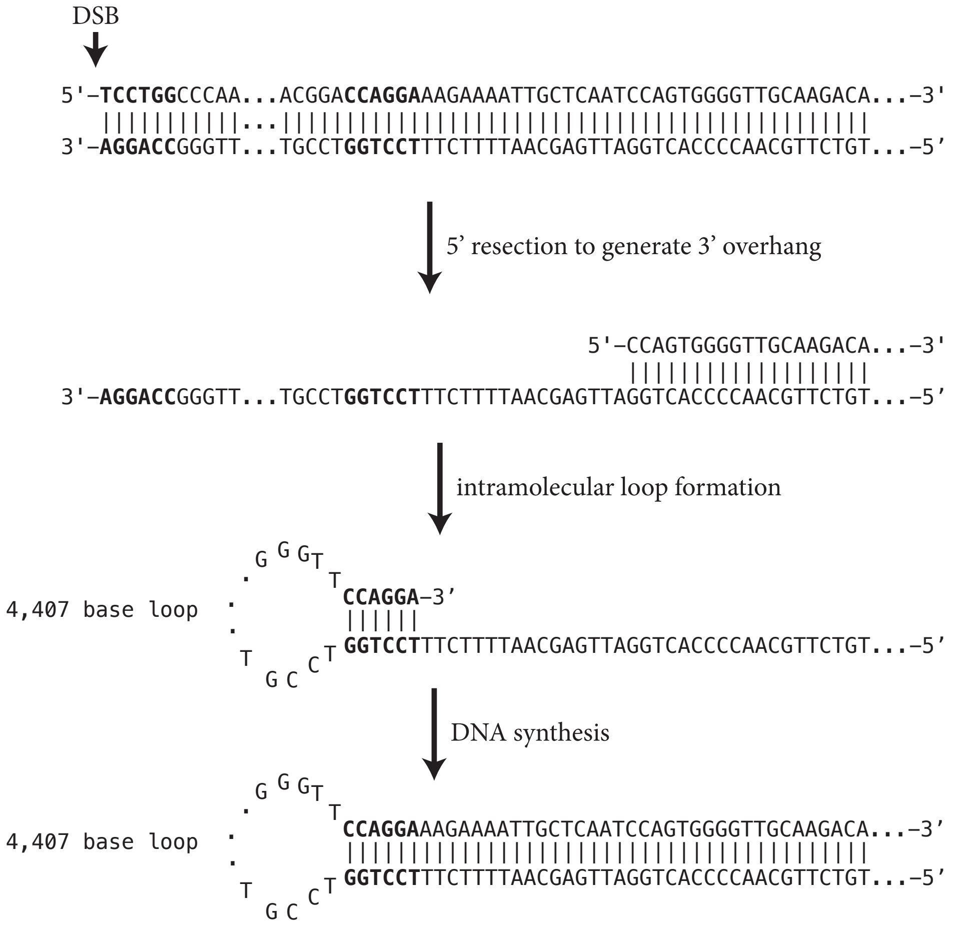

S12 Fig.

G. **Isolate 327 (*tel1 cdc73*)**

Mapped junction sequence in *NAT* marker

```
                                               KX377639.1 977-:
TGCGGGGGGTGCGGGTGGACCCGCCCCTGACCAAGGTGTT:CCCCG:
TGCGGGGGGTGCGGGTGGACCCGCCCCTGACCAAGGTGTT:CCCCG:GGACACTGGTGCGGTACCGGTAAGCCGTGTCGTCAAGAGTGGTACC
                                        :CCCCG:GGACACTGGTGCGGTACCGGTAAGCCGTGTCGTCAAGAGTGGTACC
                                        :KX377639.1 847-
```

H. **Potential inversion formation mechanism**

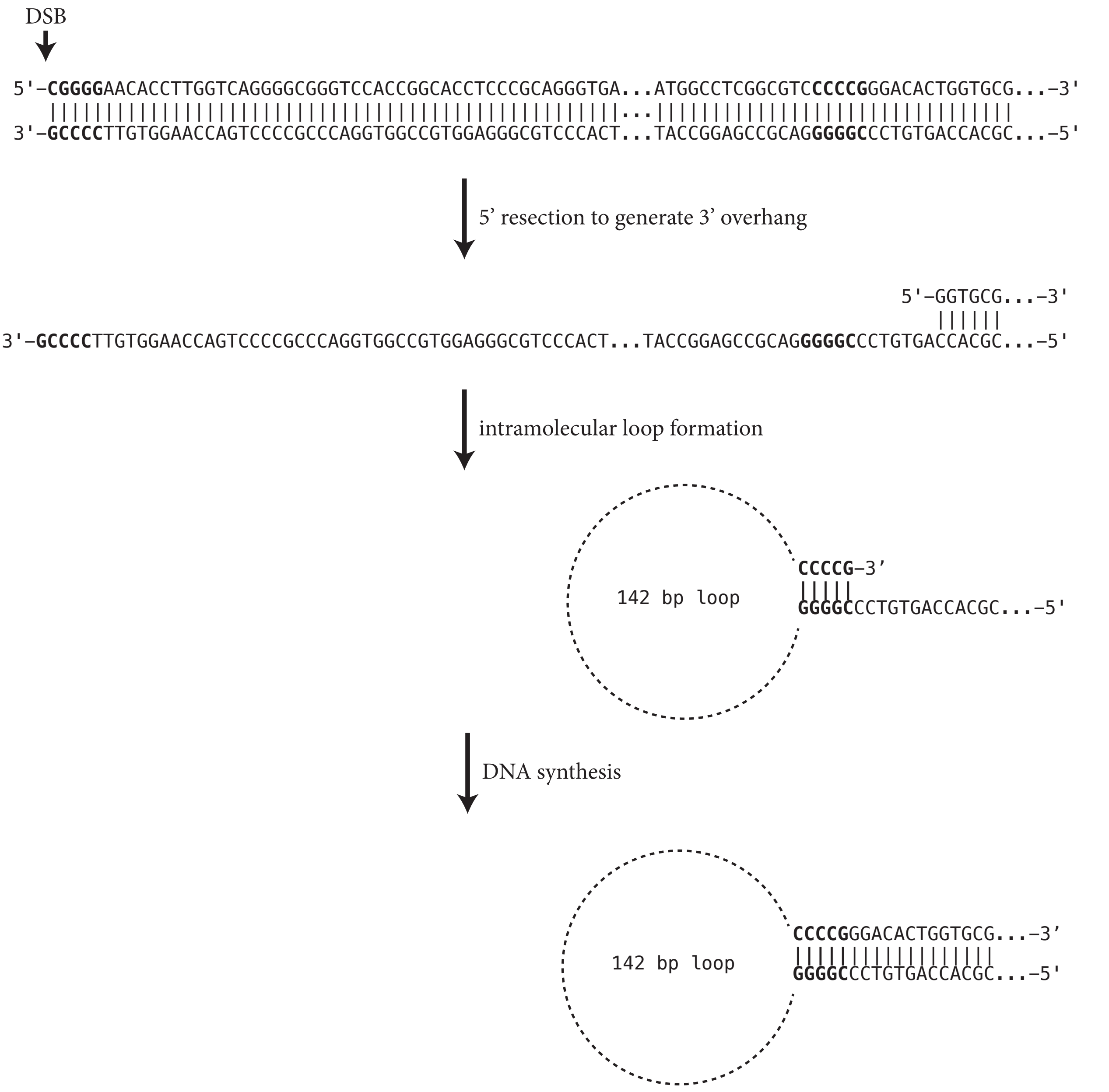

S12 Fig.

I. Isolate 349 (*yku80 cdc73*)

Mapped junction sequence in *NAT* marker

```
                  :KX377639.1 1219–
                  :GAGGTGCC:CGGCGCCCCGCTCGCGGGCGAACTCCGTCGCGAGCCCCATCAACGCGCGCC
                   |||||||| |||||||||||||||||||||||||||||||||||||||||||||||||||
    GCTTCACCCTGCGG:GAGGTGCC:CGGCGCCCCGCTCGCGGGCGAACTCCGTCGCGAGCCCCATCAACGCGCGCC
    |||||||||||||| ||||||||
    GCTTCACCCTGCGG:GAGGTGCC:
           KX377639.1 944+:
```

J. Potential inversion formation mechanism

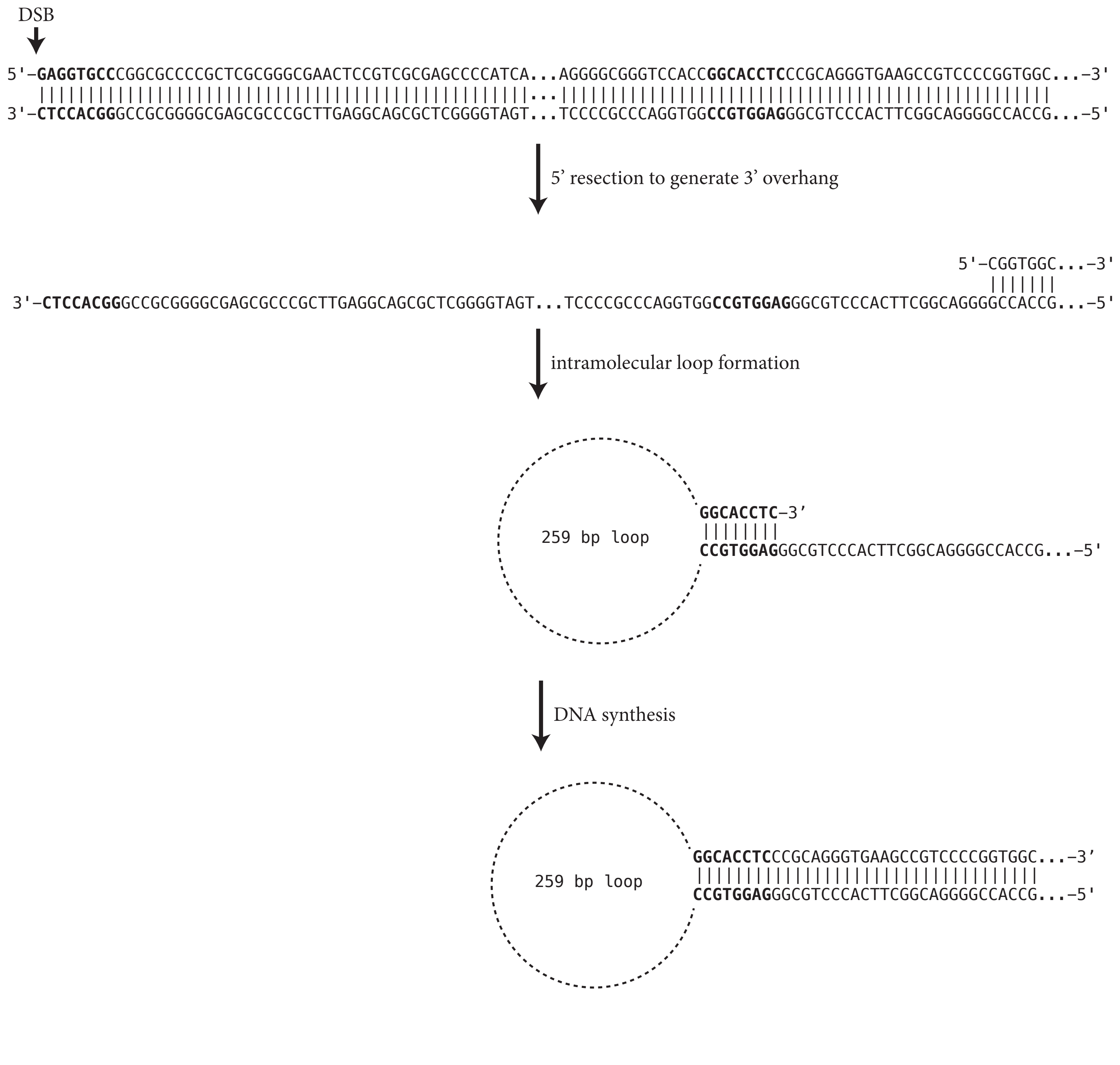

Supplement: S12 Fig — a, c, e, g, i. Inversion junction sequences identified in different GCRs. b, d, f, h, j. The inversion junction can be formed by 5’ resection from a DSB to generate a 3’-overhang. Intramolecular loop formation mediated by intra-strand base pairing generates a 3’ primer terminus that can be extended by DNA polymerases. This initial hairpin-capped chromosome will generate a dicentric chromosome upon replication, which is unstable and undergoes additional rounds of rearrangement. See Fig 7 for an alternative mechanism involving Break-induced Replication. (PDF) [file pgen.1007170.s012.pdf]
