# Supplementary material for: Cdc73 suppresses genome instability by mediating telomere homeostasis
Source: PLoS Genet. 2018 Jan 10;14(1):e1007170. doi: 10.1371/journal.pgen.1007170 (PMC5779705; doi:10.1371/journal.pgen.1007170)
Supplement: S13 Fig — a, c, e, g, i, k, m, o. Diagram of the secondary HR event. b, d, f, h, j, l, n, p. Junction sequences and alignments between the GCR and participating chromosomes identifies the novel junction sequences displayed as in S9 Fig. See Fig 7 for an alternative mechanism involving Break-induced Replication. (PDF) [file pgen.1007170.s013.pdf]

S13 Fig.

Isolate 349 (*yku80 cdc73*), junction sequence obtained by linkage to chrV:135,468-135,656 unique region

A.

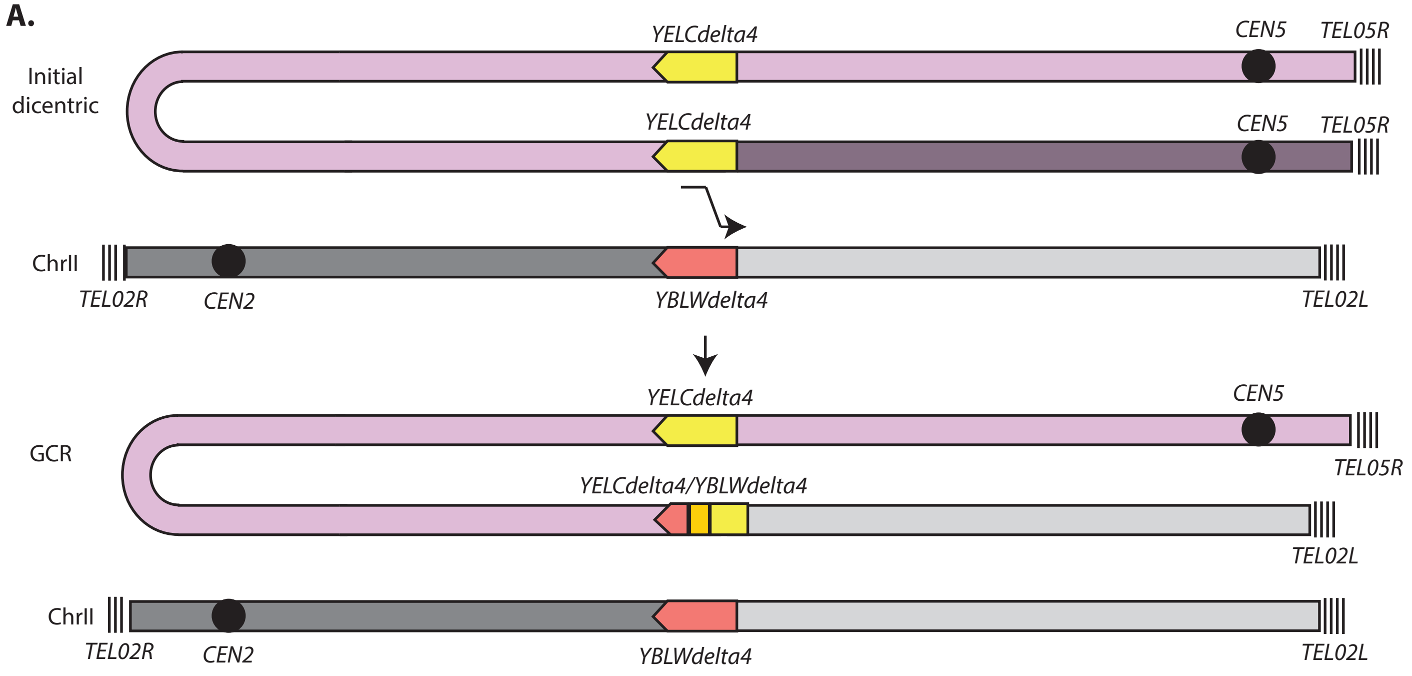

B.

```
ChrII    29972                 AGAGAATGTGGATTTTGATGTAATTGTTGGGATTCCATTGTGAT
                               ||||||||||||||||||||||||||||||||||||||||||||
Junction       TAATTAGGGTTATTGAAGAGAATGTGGATTTTGATGTAATTGTTGGGATTCCATTGTGAT
               ||||||||||||||||||||||||||||||||||||||||||||||||||||||||||||
ChrV    135595 TAATTAGGGTTATTGAAGAGAATGTGGATTTTGATGTAATTGTTGGGATTCCATTGTGAT

ChrII    29928 TAAGGCTATAATATTAGGTATGTAGATATACTAGAAGTTCTCCTCAAGGATTTAGGAATC
               ||||||||||||||||||||||||||||||||||||||||||||| ||||||||||||||
Junction 500   TAAGGCTATAATATTAGGTATGTAGATATACTAGAAGTTCTCCTCGAGGATTTAGGAATC
               ||||||||||||||||||||||||||||||||||||||||||||||||||||||||||||
ChrV    135655 TAAGGCTATAATATTAGGTATGTAGATATACTAGAAGTTCTCCTCGAGGATTTAGGAATC

ChrII    29868 CATG:AAAGGGAATCTGCAATTCTACACAATTCTATAAATATTATTATCATC:ATTTTATAT
               |||  ||||||||||||||||||||||||||||||||||||||||||||||| |||||||||
Junction       CATA:AAAGGGAATCTGCAATTCTACACAATTCTATAAATATTATTATCATC:ATTTTATAT
               |||| |||||||||||||||||||||||||||||||||||||||||||||||  ||||||||
ChrV    135715 CATA:AAAGGGAATCTGCAATTCTACACAATTCTATAAATATTATTATCATC:GTTTTATAT

ChrII    29808 GTTAATATTCATTGATCCTATTACATTATCAATCCTTGCGTTTCAGCTTCCACTAATTTA
               ||||||||||||||||||||||||||||||||||||||||||||||||||||||||||||
Junction       GTTAATATTCATTGATCCTATTACATTATCAATCCTTGCGTTTCAGCTTCCACTAATTTA
               ||||||||||||||||||||||||||||||||||||||||||||||||||||||||||||
ChrV    135775 GTTAATATTCATTGATCCTATTACATTATCAATCCTTGCGTTTCAGCTTCCACTAATTTA

ChrII    29748 GATGACTATTTCTCATCATTTGTGTCATCTTCTAACACCGTATATGATAATATACTAGTA
               ||||||||||||||||||||||||||||||||||||||||||||||||||||||||||||
Junction       GATGACTATTTCTCATCATTTGTGTCATCTTCTAACACCGTATATGATAATATACTAGTA
               |||||||||||||||||||||| |||||||||||||||||||||||||||||||||||||
ChrV    135835 GATGACTATTTCTCATCATTTGCGTCATCTTCTAACACCGTATATGATAATATACTAGTA

ChrII    29688 ACGTAAATACTAGTTAGTAGATGATAGTTGATTTTTATTCCAACAATTATATGTGCTTGT
               ||||||||||||||||||||||||||||||||||||||||||||||||||||||||||||
Junction       ACGTAAATACTAGTTAGTAGATGATAGTTGATTTTTATTCCAACAATTATATGTGCTTGT
               ||||||||||||||||||||||||||||||||||||||||||||| ||||
ChrV    135895 ACGTAAATACTAGTTAGTAGATGATAGTTGATTTTTATTCCAACAGTTAT

ChrII    29628 ATTCAGCTCTTTATTGAGTTTGATCACTGTTTCTCAATCTTCATGTCATTCTCTTCCACC
               ||||||||||||||||||||||||||||||||||||||||||||||||||||||||||||
Junction       ATTCAGCTCTTTATTGAGTTTGATCACTGTTTCTCAATCTTCATGTCATTCTCTTCCACC
```

S13 Fig.

Isolate 352 (*yku80 cdc73*), junction sequence obtained by linkage to chrV:135,468-135,656 unique region

C.

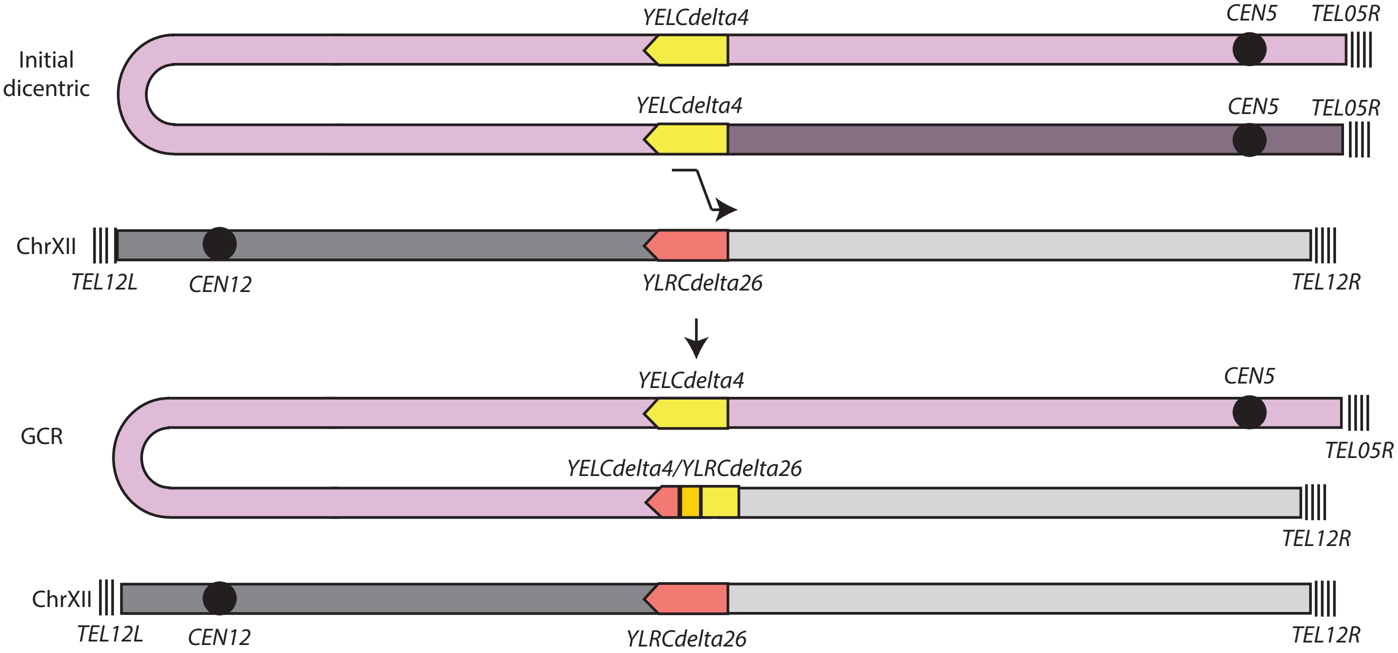

D.

```
ChrV    135554 AAACGAAGCATTGGTTGTTCATTGATTGCAACAGTAATGAATAATTAGGGTTATTGAAGA
               ||||||||||||||||||||||||||||||||||||||||||||||||||||||||||||
Junction       AAACGAAGCATTGGTTGTTCATTGATTGCAACAGTAATGAATAATTAGGGTTATTGAAGA
                                                                        |||
ChrXII  981372                                                          AGA

ChrV    135614 GAATGTGGATTTTGATGTAATTGTTGGGATTCCATTGTGATTAAGGCTATAATATTAGGT
               ||||||||||||||||||||||||||||||||||||||||||||||||||||||||||||
Junction       GAATGTGGATTTTGATGTAATTGTTGGGATTCCATTGTGATTAAGGCTATAATATTAGGT
               ||||||||||||||||||||||||||||||||||||||||||||||||||||||||||||
ChrXII  981375 GAATGTGGATTTTGATGTAATTGTTGGGATTCCATTGTGATTAAGGCTATAATATTAGGT

ChrV    135674 ATGTAGATATACTAGAAGTTCTCCTCG:AGGATTTAGGAATCCATAAAAGGGAATCTGCAA
               ||||||||||||||||||||||||||| ||||||||||||||||||||||||||||||||
Junction       ATGTAGATATACTAGAAGTTCTCCTCG:AGGATTTAGGAATCCATAAAAGGGAATCTGCAA
               ||||||||||||||||||||||||||  ||||||||||||||||||||||||||||||||
ChrXII  981435 ATGTAGATATACTAGAAGTTCTCCTCA:AGGATTTAGGAATCCATAAAAGGGAATCTGCAA

ChrV    135734 TTCTACACAATTCTATAAATATTATTATCATCGTTTTATATGTTAATATTCATTGATCCT
               ||||||||||||||||||||||||||||||||||||||||||||||||||||||||||||
Junction       TTCTACACAATTCTATAAATATTATTATCATCGTTTTATATGTTAATATTCATTGATCCT
               ||||||||||||||||||||||||||||||||||||||||||||||||||||||||||||
ChrXII  981495 TTCTACACAATTCTATAAATATTATTATCATCGTTTTATATGTTAATATTCATTGATCCT

ChrV    135794 ATTACATTATCAATCCTTGCGTTTCAGCTTCCACTAATTTAGATGACTATTTCTCATCAT
               ||||||||||||||||||||||||||||||||||||||||||||||||||||||||||||
Junction       ATTACATTATCAATCCTTGCGTTTCAGCTTCCACTAATTTAGATGACTATTTCTCATCAT
               ||||||||||||||||||||||||||||||||||||||||||||||||||||||||||||
ChrXII  981555 ATTACATTATCAATCCTTGCGTTTCAGCTTCCACTAATTTAGATGACTATTTCTCATCAT

ChrV    135854 TTGCGTCATCTTCTAACACCGTATATGATAATATACTAGTAACGTAAATACTAGTTAGTA
               ||||||||||||||||||||||||||||||||||||||||||||||||||||||||||||
Junction       TTGCGTCATCTTCTAACACCGTATATGATAATATACTAGTAACGTAAATACTAGTTAGTA
               ||||||||||||||||||||||||||||||||||||||||||||||||||||||||||||
ChrXII  981615 TTGCGTCATCTTCTAACACCGTATATGATAATATACTAGTAACGTAAATACTAGTTAGTA

ChrV    135914 GATGATAGTTGATTTTTATTCCAACAG:TT
               |||||||||||||||||||||||||||  |
Junction       GATGATAGTTGATTTTTATTCCAACAG:GTTCACACGAAGATTGAAAGTGTGGCCACGCTA
               ||||||||||||||||||||||||||| ||||||||||||||||||||||||||||||||
ChrXII  981675 GATGATAGTTGATTTTTATTCCAACAG:GTTCACACGAAGATTGAAAGTGTGGCCACGCTA

Junction       ATTTAGTTTATTAGGGAAATGCTCCTGGTCAGTTCTAATTCACCCAACGTAGCGCGGAAT
               ||||||||||||||||||||||||||||||||||||||||||||||||||||||||||||
ChrXII  981735 ATTTAGTTTATTAGGGAAATGCTCCTGGTCAGTTCTAATTCACCCAACGTAGCGCGGAAT
```

S13 Fig.

Isolate 330 (*tel1 cdc73*), junction sequence obtained by linkage to chrV:135,468-135,656 unique region

E.

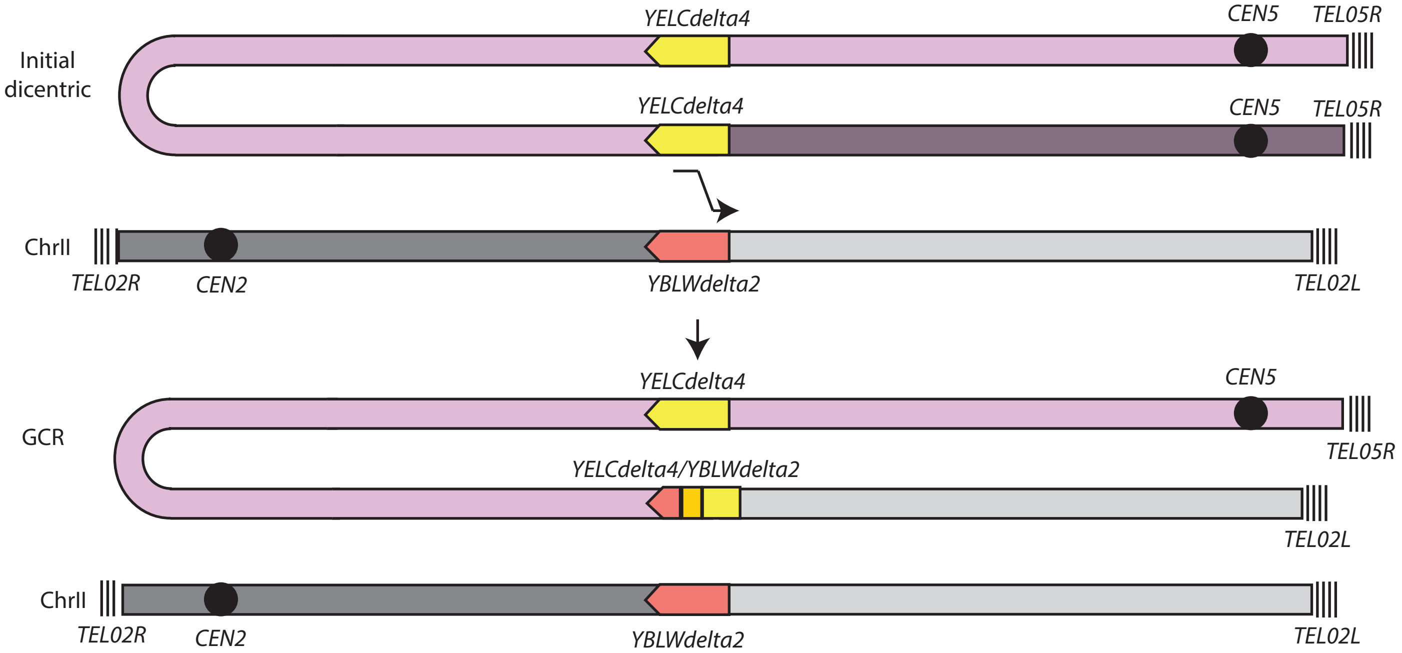

F.

```
Junction        TGTCTGTGTATGTCTAAAACGAAGCATTGGTTGTTCATTGATTGCAACAGTAATGAATAA
                ||||||||||||||||||||||||||||||||||||||||||||||||||||||||||||
ChrV    135538  TGTCTGTGTATGTCTAAAACGAAGCATTGGTTGTTCATTGATTGCAACAGTAATGAATAA

ChrII     9428        TTGGTGTAGAGAATGTGGATTTTGATGTAATTGTTGGGATTCCATTGTGATTAA
                      ||  || ||||||||||||||||||||||||||||||||||||||||||||||||
Junction        TTAGGGTTATTGAAGAGAATGTGGATTTTGATGTAATTGTTGGGATTCCATTGTGATTAA
                ||||||||||||||||||||||||||||||||||||||||||||||||||||||||||||
ChrV    135598  TTAGGGTTATTGAAGAGAATGTGGATTTTGATGTAATTGTTGGGATTCCATTGTGATTAA

ChrII     9374  GGCTATAATATTAGGTATGTAGATATACTAGAAGTTCTCCTCC:AGGATTTAGGAATCCAT
                ||||||||||||||||||||||||||||||||||||||||||  |||||||||||||||||
Junction        GGCTATAATATTAGGTATGTAGATATACTAGAAGTTCTCCTCG:AGGATTTAGGAATCCAT
                ||||||||||||||||||||||||||||||||||||||||||| |||||||||||||||||
ChrV    135658  GGCTATAATATTAGGTATGTAGATATACTAGAAGTTCTCCTCG:AGGATTTAGGAATCCAT

ChrII     9314  AAAAGGGAATCTGCAATTCTACACAATTCTATAAATATTATTATCATCGTTTTATATGTT
                ||||||||||||||||||||||||||||||||||||||||||||||||||||||||||||
Junction        AAAAGGGAATCTGCAATTCTACACAATTCTATAAATATTATTATCATCGTTTTATATGTT
                ||||||||||||||||||||||||||||||||||||||||||||||||||||||||||||
ChrV    135718  AAAAGGGAATCTGCAATTCTACACAATTCTATAAATATTATTATCATCGTTTTATATGTT

ChrII     9254  AATATTCATTGATCCTATTACATTATCAATCCTTGCGTTTCAGCTTCCACTAATTTAGAT
                ||||||||||||||||||||||||||||||||||||||||||||||||||||||||||||
Junction        AATATTCATTGATCCTATTACATTATCAATCCTTGCGTTTCAGCTTCCACTAATTTAGAT
                ||||||||||||||||||||||||||||||||||||||||||||||||||||||||||||
ChrV    135778  AATATTCATTGATCCTATTACATTATCAATCCTTGCGTTTCAGCTTCCACTAATTTAGAT

ChrII     9194  GACTATTTCTCATCATTTGCGTCATCTTCTAACACCGTATATGATAATATACTAGTAACG
                ||||||||||||||||||||||||||||||||||||||||||||||||||||||||||||
Junction        GACTATTTCTCATCATTTGCGTCATCTTCTAACACCGTATATGATAATATACTAGTAACG
                ||||||||||||||||||||||||||||||||||||||||||||||||||||||||||||
ChrV    135838  GACTATTTCTCATCATTTGCGTCATCTTCTAACACCGTATATGATAATATACTAGTAACG

ChrII     9134  TAAATACTAGTTAGTAGATGATAGTTGATTTTTATTCCAACA:ATTGGAATCCTCAAAATG
                |||||||||||||||||||||||||||||||||||||||||| ||||||||||||||||||
Junction        TAAATACTAGTTAGTAGATGATAGTTGATTTTTATTCCAACA:ATTGGAATCCTCAAAATG
                ||||||||||||||||||||||||||||||||||||||||||  ||
ChrV    135898  TAAATACTAGTTAGTAGATGATAGTTGATTTTTATTCCAACA:GTT

ChrII     9074  GAATCGGTATTTCCACATAATAGTATTACTACTTTTTTTTTTTTTTTTTTTTCATTTAT
                |||||||||||||||||||||||||||||||||||||||||||||||||||||||||||
Junction        GAATCGGTATTTCCACATAATAGTATTACTACTTTTTTTTTTTTTTTTTTTTCATTTAT
```

S13 Fig.

Isolate 326 (*tel1 cdc73*), junction sequence obtained by linkage to chrV:135,468-135,656 unique region

G.

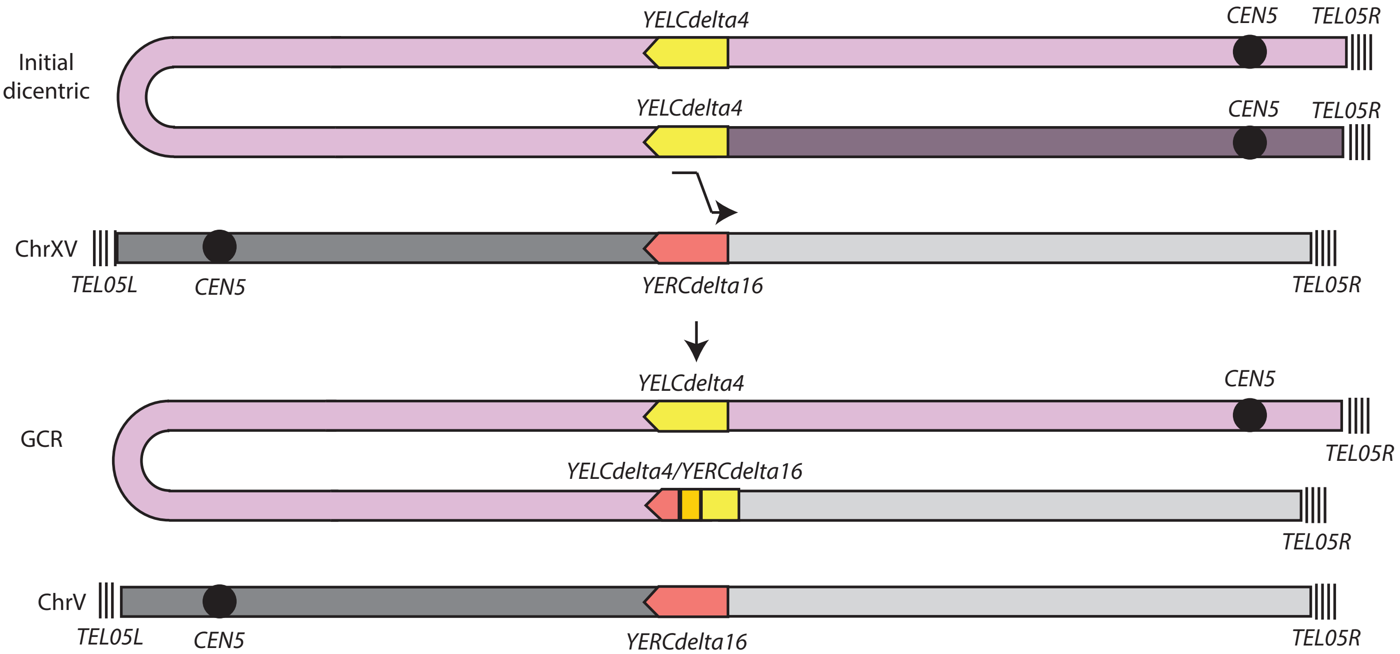

H.

```
Junction        TGTCTGTGTATGTCTAAAACGAAGCATTGGTTGTTCATTGATTGCAACAGTAATGAATAA
                ||||||||||||||||||||||||||||||||||||||||||||||||||||||||||||
ChrV    135538  TGTCTGTGTATGTCTAAAACGAAGCATTGGTTGTTCATTGATTGCAACAGTAATGAATAA

ChrV    435946            TGTAGAGAATGTGGATTTTGATGTAATTGTTGGGATTCCATTGTGATTAA
                          || |||||||||||||||||||||||||||||||||||||||||||||||
Junction        TTAGGGTTATTGAAGAGAATGTGGATTTTGATGTAATTGTTGGGATTCCATTGTGATTAA
                ||||||||||||||||||||||||||||||||||||||||||||||||||||||||||||
ChrV    135598  TTAGGGTTATTGAAGAGAATGTGGATTTTGATGTAATTGTTGGGATTCCATTGTGATTAA

ChrV    435996  GGCTATAATATTAGGTATGTAGATATACTAGAAGTTCTCCTCT:AGGATTTAGGAATCCAT
                ||||||||||||||||||||||||||||||||||||||||||  |||||||||||||||||
Junction        GGCTATAATATTAGGTATGTAGATATACTAGAAGTTCTCCTCG:AGGATTTAGGAATCCAT
                |||||||||||||||||||||||||||||||||||||||||||  |||||||||||||||||
ChrV    135658  GGCTATAATATTAGGTATGTAGATATACTAGAAGTTCTCCTCG:AGGATTTAGGAATCCAT

ChrV    436056  AAAAGGGAATCTGCAATTCTACACAATTCTATAAATATTATTATCATCGTTTTATATGTT
                ||||||||||||||||||||||||||||||||||||||||||||||||||||||||||||
Junction        AAAAGGGAATCTGCAATTCTACACAATTCTATAAATATTATTATCATCGTTTTATATGTT
                ||||||||||||||||||||||||||||||||||||||||||||||||||||||||||||
ChrV    135718  AAAAGGGAATCTGCAATTCTACACAATTCTATAAATATTATTATCATCGTTTTATATGTT

ChrV    436116  AATATTCATTGATCCTATTACATTATCAATCCTTGCGTTTCAGCTTCCACTAATTTAGAT
                ||||||||||||||||||||||||||||||||||||||||||||||||||||||||||||
Junction        AATATTCATTGATCCTATTACATTATCAATCCTTGCGTTTCAGCTTCCACTAATTTAGAT
                ||||||||||||||||||||||||||||||||||||||||||||||||||||||||||||
ChrV    135778  AATATTCATTGATCCTATTACATTATCAATCCTTGCGTTTCAGCTTCCACTAATTTAGAT

ChrV    436176  GACTATTTCTCATCATTTGCGTCATCTTCTAACACCGTATATGATAATATACTAGTAACG
                ||||||||||||||||||||||||||||||||||||||||||||||||||||||||||||
Junction        GACTATTTCTCATCATTTGCGTCATCTTCTAACACCGTATATGATAATATACTAGTAACG
                ||||||||||||||||||||||||||||||||||||||||||||||||||||||||||||
ChrV    135838  GACTATTTCTCATCATTTGCGTCATCTTCTAACACCGTATATGATAATATACTAGTAACG

ChrV    436236  TAAATACTAGTTAGTAGATGATAGTTGATTTTTATTCCAACA:CTATTAATCATACAAATA
                |||||||||||||||||||||||||||||||||||||||||| ||||||||||||||||||
Junction        TAAATACTAGTTAGTAGATGATAGTTGATTTTTATTCCAACA:CTATTAATCATACAAATA
                ||||||||||||||||||||||||||||||||||||||||||
ChrV    135898  TAAATACTAGTTAGTAGATGATAGTTGATTTTTATTCCAACA:

ChrV    436296  CTTTGAAAACTTCTTTAAATAATCTCATAAATCTTTACATTGAACTAGGATGATAAAA
                ||||||||||||||||||||||||||||||||||||||||||||||||||||||||||
Junction        CTTTGAAAACTTCTTTAAATAATCTCATAAATCTTTACATTGAACTAGGATGATAAAA
```

S13 Fig.

Isolate 306, 308 (*cdc73*), junction sequence obtained by linkage to chrV:134,200-135,652 unique region

I.

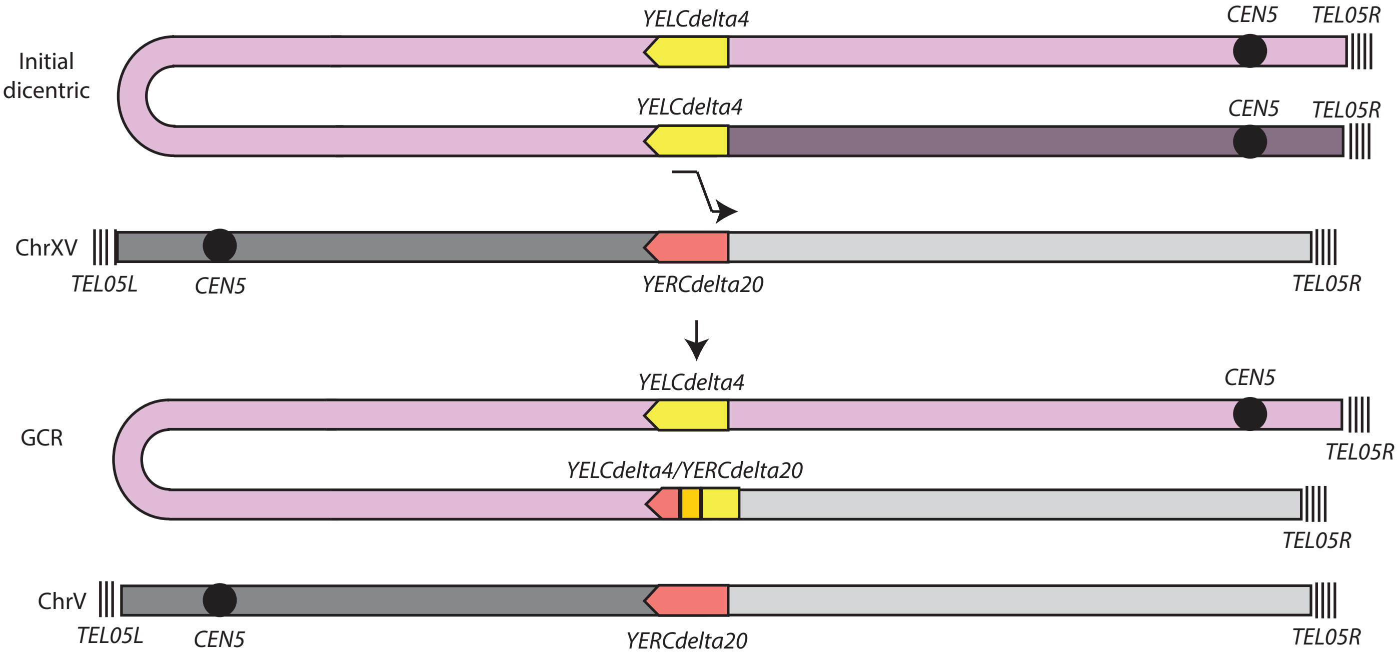

J.

```
ChrV   135590 ATGAATAATTAGGGTTATTGAAGAGAATGTGGATTTTGATGTAATTGTTGGGATTCCATT
306           ATGAATAATTAGGGTTATTGAAGAGAATGTGGATTTTGATGTAATTGTTGGGATTCCATT
308           ATGAATAATTAGGGTTATTGAAGAGAATGTGGATTTTGATGTAATTGTTGGGATTCCATT

ChrV   135650 GTGATTAAGGCTATAATATTAGGTATGTAGATATACTAGAAGTTCTCCTCGAGGATTTAG
306           GTGATTAAGGCTATAATATTAGGTATGTAGATATACTAGAAGTTCTCCTCGAGGATTTAG
308           GTGATTAAGGCTATAATATTAGGTATGTAGATATACTAGAAGTTCTCCTCGAGGATTTAG

ChrV   135710 GAATCCATAAAAGGGAATCTGCAATTCTACACAATTCTATAAATATTATTATCATC:GTTT
306           GAATCCATAAAAGGGAATCTGCAATTCTACACAATTCTATAAATATTATTATCATC:GTTT
308           GAATCCATAAAAGGGAATCTGCAATTCTACACAATTCTATAAATATTATTATCATC:GTTT
ChrV 449147                                                           :GTTT

ChrV   135770 TATATGTT:A:ATATTCATTGATCCTATTACATTATCAATCCTTGCGTTTCAGCTTCCACTA
306           TATATGTT:A:ATATTCATTGATCCTATTACATTATCAATCCTTGCGTTTCAGCTTCCACTA
308           TATATGTT:A:ATATTCATTGATCCTATTACATTATCAATCCTTGCGTTTCAGCTTCCACTA
ChrV   449151 TATATGTT:T:ATATTCATTGATCCTATTACATTATCAATCCTTGCGTTTCAGCTTCCACTA

ChrV   135830 ATTTAGATGACTATTTCTCATCATTTGCGTCATCTTCTAACACCGTATATGATAATATAC
306           ATTTAGATGACTATTTCTCATCATTTGCGTCATCTTCTAACACCGTATATGATAATATAC
308           ATTTAGATGACTATTTCTCATCATTTGCGTCATCTTCTAACACCGTATATGATAATATAC
ChrV   449211 ATTTAGATGACTATTTCTCATCATTTGCGTCATCTTCTAACACCGTATATGATAATATAC

ChrV   135890 TAGTAA:C:GTAAATACTAGTTAGTAGATGATAGTTGATTT:TTATTCCAACA:
306           TAGTAA:C:GTAAATACTAGTTAGTAGATGATAGTTGATTT:CTATTCCAACA:CATCTAC
308           TAGTAA:C:GTAAATACTAGTTAGTAGATGATAGTTGATTT:CTATTCCAACA:CATCTAC
ChrV   449271 TAGTAA:T:GTAAATACTAGTTAGTAGATGATAGTTGATTT:CTATTCCAACA:CATCTAC

306            TAACTAGTATTTACGTTACTAGTATATTATCATATACG
308            TAACTAGTATTTACGTTACTAGTATATTATCATATACG
ChrV   449328  TAACTAGTATTTACGTTACTAGTATATTATCATATACG
```

S13 Fig.

Isolate 322 (*tel1 cdc73*), junction sequence obtained by linkage to chrV:138,000..138,400 region

K.

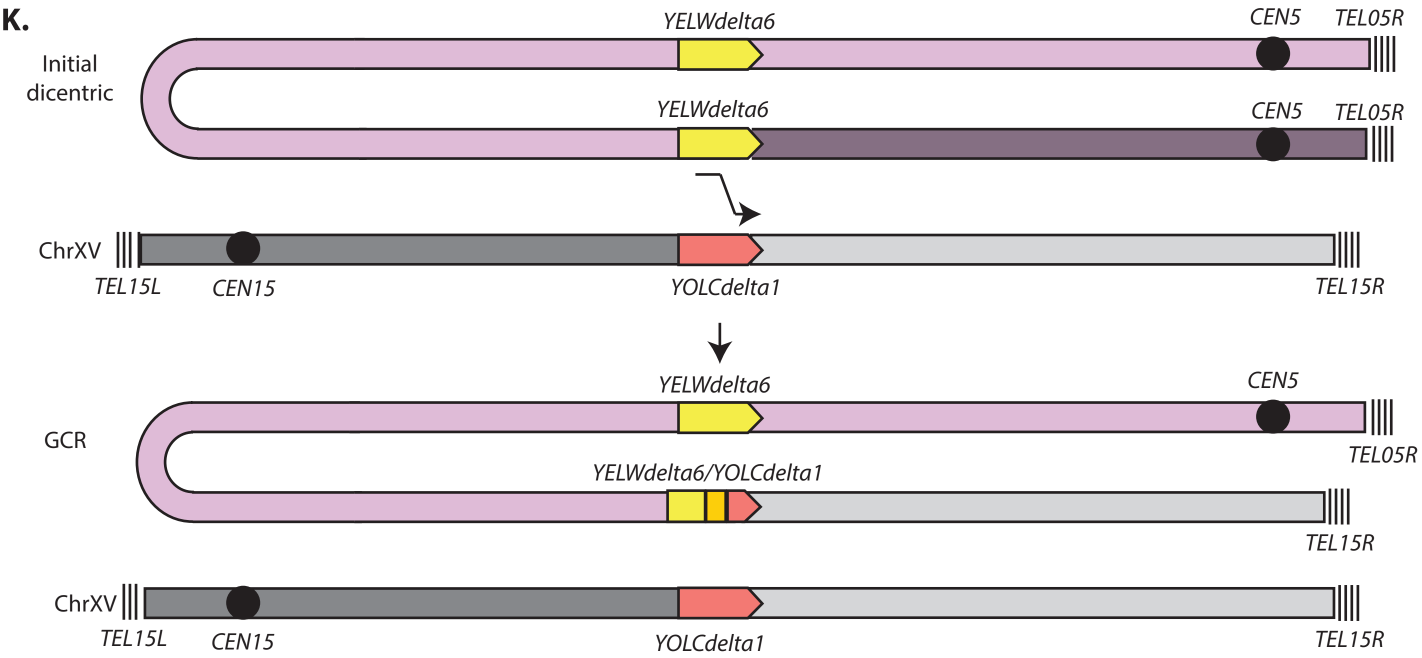

L.

```
ChrV 138321   TCATCGAAGTTAGAAGAAGCTGAAATGCAAGGATTGATAATGTAATAGAATTTAATGAAACATATAAAACGGAATGAGGA
              ||||||||||||||||||||||||||||||||||||||||||||||||||||||||||||||||||||||||||||||||
Junction      TCATCGAAGTTAGAAGAAGCTGAAATGCAAGGATTGATAATGTAATAGAATTTAATGAAACATATAAAACGGAATGAGGA
              || || ||  ||   |||  |||||| |||||| |||||||||||||| |  ||||||||||||||||||||||||||||
Chr XV 3124   TCGTCCAATCTAATGGAAAATGAAATACAAGGAGTGATAATGTAATAGGA--TAATGAAACATATAAAACGGAATGAGGA

ChrV 138401   ATAATCGTAATATTAGTATGTAAAAATATGGATTCCATTTTGAGGATTCCTATATCCATGAGGAGAACTTCTAGTATATG
              ||||||||||||||||||||||||||||||||||||||||||||||||||||||||||||||||||||||||||||||||
Junction      ATAATCGTAATATTAGTATGTAAAAATATGGATTCCATTTTGAGGATTCCTATATCCATGAGGAGAACTTCTAGTATATG
              |||||||||||||||||||| | |||||||||||||||||||||||||||| |||||  |||||||||||||||||||| 
ChrXV 3046    ATAATCGTAATATTAGTATGCAGAAATATGGATTCCATTTTGAGGATTCCTGTATCCTCGAGGAGAACTTCTAGTATATT

ChrV 138481   CTGTATACATAATAC:TATAGCCTT:GATCAACAATGGAACCCCAACAATTATCTCACAATTCACCCATTTCTC
              ||||||||||||||| |||||||||  ||||||||||||| |||||||||| ||||| |||||||||| |||||
Junction      CTGTATACATAATAC:TATAGCCTT:TATCAACAATGGAATCCCAACAATTCTCTCAAAATTCACCCAATTCTCACCATCAA
              |||| ||| |||||  ||||||||| ||||||||||||||||||||||||||||||||||||||||||||||||||||||||
ChrXV 2966    CTGTGTACCTAATAT:TATAGCCTT:TATCAACAATGGAATCCCAACAATTCTCTCAAAATTCACCCAATTCTCACCATCAA

Junction      AGAAGAAGATTTAATGGTTTGAAATAAACTGAAGAATAAAATTTACATAATAAAGCGTATATTCTTCAACTTTTTGAATT
              ||||||||||||||||||||||||||||||||||||||||||||||||||||||||||||||||||||||||||||||||
ChrXV 2886    AGAAGAAGATTTAATGGTTTGAAATAAACTGAAGAATAAAATTTACATAATAAAGCGTATATTCTTCAACTTTTTGAATT
```

S13 Fig.

Isolate 304 (*cdc73*), junction sequence obtained by linkage to chrV:138,279- unique region

M.

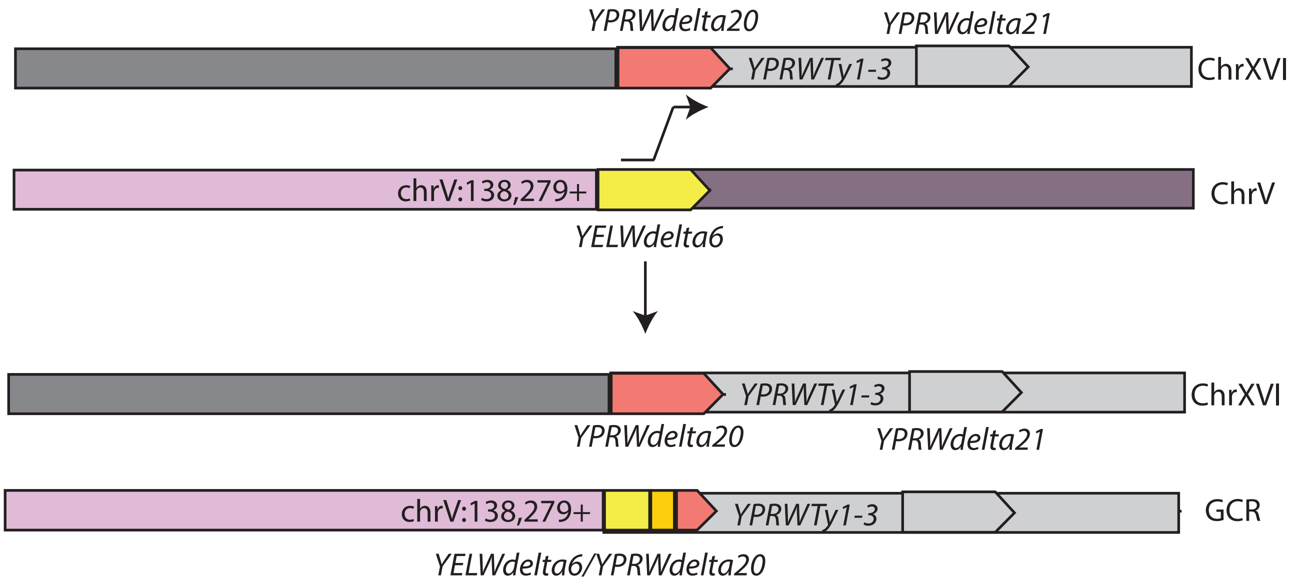

N.

```
ChrV:138241     ATCGTCTATCAACTAGTAGCTATACTACTAATATATTATCATATACGGTGTTAGATGATGACATAAGTTATGAGAAGC
                ||||||||||||||||||||||||||||||||||||||||||||||||||||||||||||||||||||||||||||||
302             ATCGTCTATCAACTAGTAGCTATACTACTAATATATTATCATATACGGTGTTAGATGATGACATAAGTTATGAGAAGC

ChrV:138319     TGTCATCGAAGTTAGAAGAAGCTGAAATGCAAGGATTGATAATGTAATAGAATTTAATG:AAACATATAAAACGGAAT
                ||||||||| ||||||||||||||||||||||||||||||||||||||||||||||||| ||||||||||||||||||
302             TGTCATCGATGTTAGAAGAAGCTGAAATGCAAGGATTGATAATGTAATAGAATTTAATG:AAACATATAAAACGGAAT
                                                                            ||||||||||||||||||
ChrXVI:844574                                                              :AAACATATAAAACGGAAT

ChrV:138396     GAGGAATAATCGTAATATTAGTATGTAAAAATATG:GATTCCATTTTGAGGATTCCTATATCC:ATGAGGAGAACTTC
                ||||||||||||||||||||||||||||||||||| |||||||||||||||||||||||||||
302             GAGGAATAATCGTAATATTAGTATGTAAAAATATG:GATTCCATTTTGAGGATTCCTATATCC:TCGAGGAGAACTTC
                ||||||||||||||||||||||||||| ||||||  ||||||||||||||||||||||||||||||||||||||||||
ChrXVI:844592   GAGGAATAATCGTAATATTAGTATGTAGAAATATA:GATTCCATTTTGAGGATTCCTATATCC:TCGAGGAGAACTTC

302             TAGTATATTCTGTATACCTAATATTATAGCCTTTATCAACAATGGAATCCCAACAATTATCTAATTACCCACATATAT
                ||||||||||||||||||||||||||||||||||||||||||||||||||||||||||||||||||||||||||||||
ChrXVI:844668   TAGTATATTCTGTATACCTAATATTATAGCCTTTATCAACAATGGAATCCCAACAATTATCTAATTACCCACATATAT
```

S13 Fig.

Isolate 353 (*yku80cdc73*), junction sequence obtained by linkage to chrV:137,890..138,319 unique region

O.

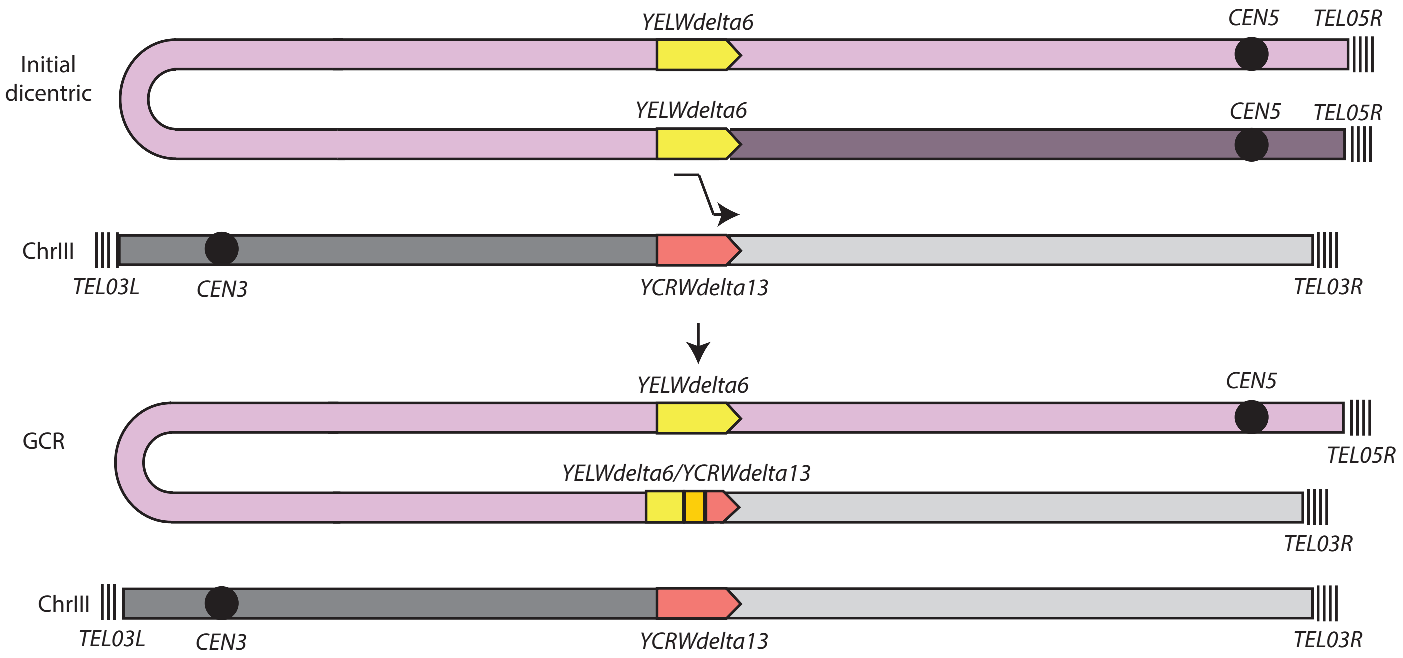

P.

```
ChrV    138135 ATTTCTATATTTTTATTCACAATATGGAAAGGAAAAACAATCTCTTAGGTTGTCCAAGAA
               ||||||||||||||||||||||||||||||||||||||||||||||||||||||||||||
Junction       ATTTCTATATTTTTATTCACAATATGGAAAGGAAAAACAATCTCTTAGGTTGTCCAAGAA

ChrV    138195 AACTTCTCTCCCTGTCATACTCAAAGTGTTGGAACAAAAATCAACTATCGTCTATCAACT
               ||||||||||||||||||||||||||||||||||||||||||||||||||||||||||||
Junction       AACTTCTCTCCCTGTCATACTCAAAGTGTTGGAACAAAAATCAACTATCGTCTATCAACT
                |||| |  |  | |  | || ||| |||||||| ||||| |||||||| ||||||||||
ChrIII  294981 CACTT-TAGCGTTTTT-TCCT-AAA-TGTTGGAATAAAAAACAACTATCATCTATCAACT

ChrV    138255 AGTAGCTATACTACTAATATATTATCAT-ATACGGTGTTAGATG:ATGACATAAG:TTATGA
               |||||||||||||||||||||||||||| ||||||||||||||| ||||||||||  |||||
Junction       AGTAGCTATACTACTAATATATTATCAT-ATACGGTGTTAGATG:ATGACATAAG:GTATGA
               |||||  | ||||| ||| | ||||||| |||| |||||| |   ||||||||||| |||||
ChrIII  295037 AGTAGTCACACTACCAATGTGTTATCATTATACTGTGTTAAACA:ATGACATAAG:GTATGA

Junction       AAATTTGTCAACGAAGTTAGAGAAAGCTGGATGCAAGGATTGATAATGTGGTAGGAAAAT
               ||||||||||||||||||||||||||||||||||||||||||||||||||||||||||||
ChrIII  295097 AAATTTGTCAACGAAGTTAGAGAAAGCTGGATGCAAGGATTGATAATGTGGTAGGAAAAT

Junction       GAAACATATAACGGAATGAGGAATAATCGTAATATCAGTATATAGAAATATAGATTCCCT
               ||||||||||||||||||||||||||||||||||||||||||||||||||||||||||||
ChrIII  295157 GAAACATATAACGGAATGAGGAATAATCGTAATATCAGTATATAGAAATATAGATTCCCT

Junction       TTTGAGGATTCCTATATCCTCGAGGAGAACTTCTAGTATATTCTATATACCTAATATTAT
               ||||||||||||||||||||||||||||||||||||||||||||||||||||||||||||
ChrIII  295217 TTTGAGGATTCCTATATCCTCGAGGAGAACTTCTAGTATATTCTATATACCTAATATTAT

Junction       TACTTTTATCTACAATGCAACCCCACAATAATATAAAAATTCACCAATTCCGCATCTGCA
               ||||||||||||||||||||||||||||||||||||||||||||||||||||||||||||
ChrIII  295277 TACTTTTATCTACAATGCAACCCCACAATAATATAAAAATTCACCAATTCCGCATCTGCA
```
