# Supplementary material for: Cdc73 suppresses genome instability by mediating telomere homeostasis
Source: PLoS Genet. 2018 Jan 10;14(1):e1007170. doi: 10.1371/journal.pgen.1007170 (PMC5779705; doi:10.1371/journal.pgen.1007170)
Supplement: S18 Fig — a. Genes in which mutations are known to cause short telomeres [46,47] and cause synergistic increases in GCR rates when combined with a cdc73Δ mutation as measured by patch tests in the dGCR assay [6] or as measured by fluctuation analysis in multiple GCR assays (Table 1). b. Genes in which mutations are known to cause short telomeres and do not cause synergistic increases in GCR rates when combined with a cdc73Δ mutation. (PDF) [file pgen.1007170.s018.pdf]

S18 Fig.

a. Genes in which mutations cause short telomeres and synergistic increases in GCR rates when combined with a *cdc73Δ* mutation

YKU70, YKU80
TEL1
RAD6
MRC1
NUP60
VPS20
SIR3, SIR4

b. Genes in which mutations cause short telomeres but do not cause synergistic increases in GCR rates when combined with a *cdc73Δ* mutation

Paf1 Complex: RTF1

RNA Pol II Mediator: PGD1, GAL11

Rpd3L Histone Deacetylase: DEP1

COMPASS Histone Methylase: BRE2

Set3C Histone Deacetylase: SIF2

C-terminal Domain Kinase: CTK1

Other Transcription and Chromatin: YTA7, WHI2, BRE1

Nonsense-mediated mRNA decay: NAM7

P-body mRNA degradation: XRN1

Vacuolar Trafficking and Homeostasis: PEP3, VAM7, VAM6, VPS53

Phospho-signalling: PTC1, GPB2

MRX Complex (Saturates assay as single mutants): MRE11, RAD50, XRS2

Ctf18-RFC-like complex: DCC1, CTF8

Other: LST7, SMI1, RPL34B

Unknown: YDL118W
