# Supplementary material for: Cdc73 suppresses genome instability by mediating telomere homeostasis
Source: PLoS Genet. 2018 Jan 10;14(1):e1007170. doi: 10.1371/journal.pgen.1007170 (PMC5779705; doi:10.1371/journal.pgen.1007170)
Supplement: S23 Fig — Nuclear localization signals for the Paf1 complex subunits were predicted using cNLS Mapper (http://nls-mapper.iab.keio.ac.jp/cgi-bin/NLS_Mapper_form.cgi; [117]). Positions of the predicted signals are indicated in the subunit sequences (panels a, c, e, g, i), and the individual signals and their scores are displayed (panels b, d, f, h, j). (PDF) [file pgen.1007170.s023.pdf]

S23 Fig.

**a.** Paf1

```
  1 MSKKQEYIAP IKYQNSLPVP QLPPKLLVYP ESPETNADSS QLINSLYIKT NVTNLIQQDE
 61 DLGMPVDLMK FPGLLNKLDS KLLYGFDNVK LDKDDRILLR DPRIDRLTKT DISKVTFLRR
121 TEYVSNTIAA HDNTSLKRKR RLDDGDSDDE NLDVNHIISR VEGTFNKTDK WQHPVKKGVK
181 MVKKWDLLPD TASMDQVYFI LKFMGSASLD TKEKKSLNTG IFRPVELEED EWISMYATDH
241 KDSAILENEL EKGMDEMDDD SHEGKIYKFK RIRDYDMKQV AEKPMTELAI RLNDKDGIAY
301 YKPLRSKIEL RRRRVNDIIK PLVKEHDIDQ LNVTLRNPST KEANIRDKLR MKFDPINFAT
361 VDEEDDEDEE QPEDVKKESE GDSKTEGSEQ EGENEKDEEI KQEKENEQDE ENKQDENRAA
421 DTPETSDAVH TEQKPEEEKE TLQEE*
```

**b.**

Predicted monopartite NLS

```
Pos 134      TSLKRKRRLD     Score 7
Pos 136      LKRKRRLDDG     Score 9
```

Predicted bipartite NLS

None

**c.** Ctr9

```
   1 MTNAMKVEGY PSMEWPTSLD IPLKASEELV GIDLETDLPD DPTDLKTLLV EENSEKEHWL
  61 TIALAYCNHG KTNEGIKLIE MALDVFQNSE RASLHTFLTW AHLNLAKGQS LSVETKEHEL
 121 TQAELNLKDA IGFDPTWIGN MLATVELYYQ RGHYDKALET SDLFVKSIHA EDHRSGRQSK
 181 PNCLFLLLRA KLLYQKKNYM ASLKIFQELL VINPVLQPDP RIGIGLCFWQ LKDSKMAIKS
 241 WQRALQLNPK NTSASILVLL GEFRESFTNS TNDKTFKEAF TKALSDLNNI FSENQHNPVL
 301 LTLLQTYYYF KGDYQTVLDI YHHRILKMSP MIAKIVLSES SFWCGRAHYA LGDYRKSFIM
 361 FQESLKKNED NLLAKLGLGQ TQIKNNLLEE SIITFENLYK TNESLQELNY ILGMLYAGKA
 421 FDAKTAKNTS AKEQSNLNEK ALKYLERYLK LTLATKNQLV ISRAYLVISQ LYELQNQYKT
 481 SLDYLSKALE EMEFIKKEIP LEVLNNLACY HFINGDFIKA DDLFKQAKAK VSDKDESVNI
 541 TLEYNIARTN EKNDCEKSES IYSQVTSLHP AYIAARIRNL YLKFAQSKIE DSDMSTEMNK
 601 LLDLNKSDLE IRSFYGWYLK NSKERKNNEK STTHNKETLV KYNSHDAYAL ISLANLYVTI
 661 ARDGKKSRNP KEQEKSKHSY LKAIQLYQKV LQVDPFNIFA AQGLAIIFAE SKRLGPALEI
 721 LRKVRDSLDN EDVQLNLAHC YLEMREYGKA IENYELVLKK FDNEKTRPHI LNLLGRAWYA
 781 RAIKERSVNF YQKALENAKT ALDLFVKESS KSKFIHSVKF NIALLHFQIA ETLRRSNPKF
 841 RTVQQIKDSL EGLKEGLELF RELNDLKEFN MIPKEELEQR IQLGETTMKS ALERSLNEQE
 901 EFEKEQSAKI DEARKILEEN ELKEQGWMKQ EEEARRLKLE KQAEEYRKLQ DEAQKLIQER
 961 EAMAISEHNV KDDSDLSDKD NEYDEEKPRQ KRKRSTKTKN SGESKRRKAA KKTLSDSDED
1021 DDDVVKKPSH NKGKKSQLSN EFIEDSDEEE AQMSGSEQNK NDDNDENNDN DDNDGLF*
```

**d.**

Predicted monopartite NLS

```
Pos 988      PRQKRKRSTK     Score 13
Pos 1002     GESKRRKAA      Score 5
```

Predicted bipartite NLS

```
Pos 986      EKPRQKRKRSTKTKNSGESKR-
     RKARQKRKRSTKTKNSGESKRRKA Score 12.1
```

**e.** Rtf1

```
  1 MSDLDEDLLA LAGADESEEE DQVLTTTSAK RAKNNDQSLS KKRRIEVGSV EDDDEEDDYN
 61 PYSVGNADYG SEEEEEANPF PLEGKYKDES DREHLESLPE MERETLLFER SQIMQKYQER
121 KLFRARGRDM KEQQQRAKND EDSRKTRAST RSTHATGHSD IKASKLSQLK KQRARKNRHY
181 SDNEDEDDEE DYREEDYKDD EGSEYGDDEE YNPFDRRDTY DKREEVEWAE EEDEQDREPE
241 ISDFNKLRIG RSFVAKFCFY PGFEDAVKGC YGRVNVGTDK RTGKTSYRMV RIERVFLQKP
301 YNMGKFYTNQ YFGVTQGKDR KVFQMNYFSD GLFAEDEYQR YLRALDNSQM IKPSLHSLSN
361 KTKEVMDFVN TPLTDKTTDE VVRHRMQFNK KLSGTNAVLE KTVLREKLQY AKETNNEKDI
421 AKYSAQLRNF EKRMSVYEKH HENDQSDIKK LGELTSKNRK LNMSNIRNAE HVKKEDSNNF
481 DSKSDPFSRL KTRTKVYYQE IQKEENAKAK EIAQQEKLQE DKDAKDKREK ELLVAQFRRL
541 GGLERMVGEL DIKFDLKF*
```

**f.**

Predicted monopartite NLS

```
Pos 36       DQSLSKKRRIE    Score 6.5
```

Predicted bipartite NLS

```
Pos 27       TSAKRAKNNDQS-
     LSKKRRIE                 Score 5
```

**g.** Leo1

```
  1 MSSESPQDQP QKEQISNNVG VTTNSTSNEE TSRSQDDNVK EVNGNDDTKE EEQEEDAELD
 61 DLFGDDNDDD DDDDVKKSET EKSDSDSDED DEGENINHRS RHRESLGLDD DEAEEQAMYT
121 RKFYGEDANN FSDQDETTHT FKEENVELVR HIIPSKANVN ETASHNEIFY ARIPNFLTID
181 PIPFDPPSFE AKVNERASNS ASREDQLDDR LIDENTVRWR YSRDKDQHVF KESNTQIVQW
241 SDGTYSLKVG EECTDILVND TSNTFLTVSH DQQELIQCYE GGEIKKTLMF IPTSTNSKIH
301 QKLSKAVIRR NQRQSKGPGT YIVSMDPEVE KKELERKQSQ ILRDRRRRQL KEKEKQESPD
361 AAFETGFRKQ NSPTTYGASR RNEYEEDDFL VDDDEEEEAA FDDEEDDNEE EEEEEDADEE
421 NASRLRNLKR EGAAMYREEE EEEKDRSETK RRRVAVIEDD EDED*
```

**h.**

Predicted monopartite NLS

```
Pos 445      DRSETKRRRVA    Score 7.5
```

Predicted bipartite NLS

```
Pos 424      RLRNLKREGAAMYR-
     EEEEEEEKDRSETKRRRV       Score 6.9
```

**i.** Cdc73

```
  1 MANSLDRLRE HLKNGDKLVL KNNEGQSTDD ITKATMVETL SSDGSTQDSF PLNEETEIEI
 61 DGSLVQLRII VHCWMNKDSS AADYLADCQN KQLTNVSFLQ RTDLINWLSG NTESSQYLKA
121 PGQKGETSDK VDIENKTLAG ELSTVKSTTS ASLENDSEVS DPVVVETMKH ERILVDHNSA
181 LRGAKPINFG YLIKDAELKL VQSIKGSLRG SKLPPGHKGA HGRISKTNGS SGGPRKDPII
241 LIPSAASSIL TVANIKQFLL ESKYVNPRNL PSVPNGLVNI EKNFERISRP IRFIIVDNTR
301 MFTKPEYWDR VVAIFTTGHT WQFNNYQWNS PQELFQRCKG YYFHFAGDSV PQHVQQWNVE
361 KVELDKNKRF KDVEVVRYFW HSLEKELISR GYR*
```

**j.**

Predicted monopartite NLS

None

Predicted bipartite NLS

None
