# Supplementary material for: Cdc73 suppresses genome instability by mediating telomere homeostasis
Source: PLoS Genet. 2018 Jan 10;14(1):e1007170. doi: 10.1371/journal.pgen.1007170 (PMC5779705; doi:10.1371/journal.pgen.1007170)
Supplement: S1 Table — (PDF) [file pgen.1007170.s024.pdf]

Supplemental Table 1. Effect of deleting Paf1 complex-encoding genes on dGCR rates.

	Wild-type *CDC73*		*yku80*Δ Mutation		*tel1*Δ Mutation	
Genotype	***RDKY***	***Can^R $5FOA^R$ Rate*** †	***RDKY***	***Can^R $5FOA^R$ Rate*** †	***RDKY***	***Can^R $5FOA^R$ Rate*** †
Wild-type	7635	8.1 [6.4-15] x 10^{-8} (1)	8339	3.29 [1.5-10] x 10^{-7} (4.1)	8340	3.38 [2.0-4.9] x 10^{-7} (4.2)
cdc73Δ	7986	7.54 [3.5-22] x 10^{-7} (9.3)	8323	1.31 [0.7-3.4] x 10^{-5} (162)	8324	1.91 [0.7-3.3] x 10^{-5} (236)
paf1Δ	7990	1.77 [0.8-2.3] x 10^{-6} (22)	8325	1.85 [0.5-56] x 10^{-5} (228)	8326	2.11 [0.3-3.8] x 10^{-5} (261)
ctr9Δ	8063	6.94 [3.4-11] x 10^{-7} (8.6)	8327	3.67 [0.6-5.8] x 10^{-6} (45)	8328	5.78 [1.9-11] x 10^{-6} (71)
rtf1Δ	7988	3.12 [0.4-14] x 10^{-7} (3.8)	8329	8.43 [3.0-13] x 10^{-7} (10)	8330	8.73 [6.1-49] x 10^{-8} (1.1)
leo1Δ	8061	3.17 [2.3-9.7] x 10^{-7} (3.9)	8331	3.75 [2.8-4.5] x 10^{-7} (4.6)	8332	2.55 [1.4-3.2] x 10^{-7} (3.2)

†Rate of accumulating Can^R $5FOA^R$ progeny. The numbers in square brackets [ ] are the 95% confidence interval limits. The number in parenthesis ( ) is the fold increase relative to the wild-type dGCR assay.
