# Supplementary material for: Cdc73 suppresses genome instability by mediating telomere homeostasis
Source: PLoS Genet. 2018 Jan 10;14(1):e1007170. doi: 10.1371/journal.pgen.1007170 (PMC5779705; doi:10.1371/journal.pgen.1007170)
Supplement: S2 Table — (PDF) [file pgen.1007170.s025.pdf]

Supplemental Table 2. Effect of the *cdc73Δ* mutation on dGCR rates.

	Wild-type *CDC73*		*cdc73*Δ Mutation	
Genotype	***RDKY***	***Can^R $5FOA^R$ Rate†***	***RDKY***	***Can^R $5FOA^R$ Rate†***
Wild-type	7635	8.1 [6.4-15] x 10^{-8} (1)	7986	7.54 [3.5-22] x 10^{-7} (9.3)
yku70Δ	8420	3.82 [2.6-9.0] x 10^{-7} (4.7)	8427	1.62 x 10^{-5} (200)
yku80Δ	8339	3.29 [1.5-10] x 10^{-7} (4.1)	8323	1.31 [0.7-3.4] x 10^{-5} (162)
tel1Δ	8340	3.38 [2.0-4.9] x 10^{-7} (4.2)	8324	1.91 [0.7-3.3] x 10^{-5} (236)
exo1Δ	8419	2.21 [1.6-2.7] x 10^{-7} (2.7)	8428	1.02 [0.8-3.5] x 10^{-5} (126)
chl4Δ	8421	8.14 [5.5-18] x 10^{-8} (1.0)	8429	2.50 [0.5-5.7] x 10^{-5} (309)
thp1Δ	8422	2.15 [0.8-12] x 10^{-7} (2.6)	8430	6.05 [4.9-6.8] x 10^{-6} (75)
mid1Δ	8423	7.66 [1.2-22] x 10^{-8} (1.0)	8431	5.12 [2.9-12] x 10^{-6} (63)
sir2Δ	8172	5.26 [3.0-7.7] x 10^{-7} (6.5)	8432	6.38 [5.2-12] x 10^{-6} (79)
sir3Δ	8173	5.40 [3.1-14] x 10^{-7} (6.7)	8433	1.63 [0.7-7.6] x 10^{-5} (201)
sir4Δ	8424	6.49 [3.1-8.9] x 10^{-7} (8.0)	8434	3.50 [1.0-5.0] x 10^{-6} (43)
rad52Δ	8425	7.00 [2.9-31] x 10^{-9} (0.09)	8435	3.44 [1.6-5.5] x10^{-7} (4.3)
dnl4Δ	8426	1.87 [1.2-2.4] x 10^{-7} (2.3)	8436	6.96 [4.6-33] x10^{-7} (8.6)
exo1Δ, yku80Δ	8463	1.44 [1.2-2.2] x 10^{-7} (1.8)	8465	6.28 [2.7-9.4] x 10^{-5} (775)
exo1Δ, tel1Δ	8464	6.13 [3.2-12] x 10^{-7} (7.6)	8466	2.05 [0.9-6.1] x 10^{-6} (25)
yku80Δ, tel1Δ	8467	2.27 [1.3-3.0] x 10^{-6} (28)	8468	1.64 [0.2-3.7] x 10^{-4} (2024)
yku80Δ, rad52Δ		ND	8437	5.12 [2.3-9.5] x 10^{-7} (6.3)
yku80Δ, dnl4Δ		ND	8438	5.19 [2.0-51] x 10^{-5} (641)
tel1Δ, rad52Δ		ND	8439	4.24 [1.7-5.8] x 10^{-7} (5.2)
tel1Δ, dnl4Δ		ND	8440	2.05 [1.9-3.8] x 10^{-5} (253)

†Rate of accumulating Can^R $5FOA^R$ progeny. The numbers in square brackets [ ] are the 95% confidence interval limits. The number in parenthesis ( ) is the fold increase relative to the wild-type dGCR assay. n.d., not determined.
