# Supplementary material for: Cdc73 suppresses genome instability by mediating telomere homeostasis
Source: PLoS Genet. 2018 Jan 10;14(1):e1007170. doi: 10.1371/journal.pgen.1007170 (PMC5779705; doi:10.1371/journal.pgen.1007170)
Supplement: S3 Table — (PDF) [file pgen.1007170.s026.pdf]

Supplemental Table 3. Statistics from Whole Genome Sequencing of sGCR-containing isolates

Sample	Relevant Gentoype	No. Read Pairs*	% Read 1 Mapped	% Read 2 Mapped	No. Uniquely Mapping Read Pairs	Median Intra-Read Pair Distance (bp)	Median Read Depth**
RDKY7964	wild-type	8,326,011 (8,659,824)	85.83%	81.63%	5,830,383	443	53
isolate 541	wild-type	9,418,388 (9,538,156)	84.04%	79.85%	6,554,625	439	59
isolate 542	wild-type	8,477,823 (8,714,627)	85.63%	81.73%	5,968,080	441	56
isolate 543	wild-type	8,390,080 (8,562,393)	74.60%	70.21%	5,079,575	455	47
isolate 544	wild-type	8,876,402 (9,251,742)	79.11%	74.18%	5,665,344	483	50
isolate 545	wild-type	9,586,438 (9,895,872)	74.86%	69.95%	5,738,797	467	52
isolate 546	wild-type	6,550,894 (8,628,109)	83.46%	79.73%	4,212,227	447	35
isolate 547	wild-type	7,238,965 (7,721,265)	84.57%	79.65%	4,934,564	456	43
isolate 548	wild-type	6,901,745 (7,849,953)	81.67%	76.79%	4, 426,736	462	38
isolate 549	wild-type	8,929,193 (9,125,134)	79.55%	74.37%	5,773,823	461	52
isolate 550	wild-type	10,560,888 (10,931,572)	85.08%	80.98%	7,402,387	437	66
isolate 551	wild-type	8,451,108 (8,618,433)	80.23%	75.61%	5,515,188	460	49
RDKY8407	*cdc73Δ*	8,914,110 (9,147,922)	95.85%	92.51%	7,077,127	396	63
isolate 301	*cdc73Δ*	25,542,591 (26,543,805)	97.54%	95.56%	21,673,833	331	188
isolate 302	*cdc73Δ*	36,011,925 (37,572,552)	97.38%	95.29%	30,328,231	337	270
isolate 303	*cdc73Δ*	15,186,423 (15,509,286)	97.54%	95.12%	12,727,638	359	112
isolate 304	*cdc73Δ*	15,661,971 (16,043,006)	97.27%	95.08%	13,128,281	352	115
isolate 305	*cdc73Δ*	12,061,802 (12,335,814)	97.50%	95.48%	10,100,220	338	84
isolate 306	*cdc73Δ*	6,044,130 (12,335,814)	97.23%	95.12%	4,996,191	340	40
isolate 307	*cdc73Δ*	8,641,341 (9,687,340)	89.69%	86.13%	6,248.712	446	56
isolate 308	*cdc73Δ*	7,241,223 (7,388,047)	96.10%	92.24%	5,596,241	405	48
isolate 309	*cdc73Δ*	9,570,960 (9,774,999)	96.36%	93.12%	7,597,366	372	68
isolate 310	*cdc73Δ*	6,466,734 (6,618,862)	90.88%	87.19%	4,727,141	413	42
isolate 311	*cdc73Δ*	7,702,605 (7,889,157)	96.55%	93.26%	6,081,972	377	52
RDKY8409	*cdc73Δ tel1Δ*	16,250,835 (16,859,605)	94.05%	90.68%	12,688,059	362	101
isolate 321	*cdc73Δ tel1Δ*	17,704,747 (18,863,922)	94.50%	90.66%	13,497,430	372	123
isolate 322	*cdc73Δ tel1Δ*	10,765,261 (11,742,490)	90.69%	86.50%	7,534,174	374	63
isolate 323	*cdc73Δ tel1Δ*	14,901,017 (15,476,541)	94.07%	89.82%	11,500,029	387	103
isolate 324	*cdc73Δ tel1Δ*	21,316,018 (22,322,374)	94.56%	91.69%	17,087,740	347	98
isolate 325	*cdc73Δ tel1Δ*	19,212,638 (20,360,546)	94.60%	91.80%	15,084,139	345	133
isolate 326	*cdc73Δ tel1Δ*	38,200,543 (42,138,033)	79.45%	77.48%	24,598,652	346	225
isolate 327	*cdc73Δ tel1Δ*	14,883,739 (16,706,910)	88.46%	85.58%	10,059,944	350	86
isolate 328	*cdc73Δ tel1Δ*	21,095,171 (22,303,025)	91.99%	88.12%	15,626,798	358	140

isolate 329	*cdc73Δ tel1Δ*	13,188,101 (13,842,756)	83.13%	79.50%	8,651,844	393	78
isolate 330	*cdc73Δ tel1Δ*	17,107,334 (18,111,500)	92.46%	89.37%	12,856,311	356	113
isolate 331	*cdc73Δ tel1Δ*	10,967,779 (11,504,956)	80.47%	77.42%	7,016,921	383	61
RDKY8411	*cdc73Δ yku80Δ*	13,275,788 (13,741,766)	88.58%	78.40%	8,777,788	361	80
isolate 345	*cdc73Δ yku80Δ*	7,890,950 (8,215,873)	91.10%	81.54%	5,349,403	343	43
isolate 346	*cdc73Δ yku80Δ*	10,255,637 (10,820,815)	89.58%	79.34%	6,636,032	350	58
isolate 347	*cdc73Δ yku80Δ*	8,669,455 (8,954,632)	91.37%	79.68%	5,825,396	379	53
isolate 348	*cdc73Δ yku80Δ*	33,001,799 (36,195,107)	88.50%	78.76%	22,253,876	348	208
isolate 349	*cdc73Δ yku80Δ*	5,821,524 (6,004,034)	88.10%	76.72%	3,654,470	385	33
isolate 350	*cdc73Δ yku80Δ*	30,483,702 (33,567,142)	89.00%	79.91%	21,059,105	338	186
isolate 351	*cdc73Δ yku80Δ*	22,349,170 (23,440,514)	88.63%	79.62%	15,204,677	344	145
isolate 352	*cdc73Δ yku80Δ*	24,388,237 (26,645,161)	86.48%	74.25%	15,370,940	360	139
isolate 353	*cdc73Δ yku80Δ*	26,410,944 (28,752,111)	86.76%	76.82%	17,343,967	354	151
isolate 354	*cdc73Δ yku80Δ*	27,886,312 (30,500,554)	89.19%	79.30%	18,761,843	358	168
isolate 355	*cdc73Δ yku80Δ*	25,630,030 (26,922,489)	89.57%	79.48%	17,807,852	356	160

*Numbers in parentheses indicate the number of reads prior to culling PCR duplicates. All reads were 50 base pairs long.
**Median read depth is the median number of times that each base in uniquely mapping regions of the nuclear genome was present within a read.
