# Supplementary material for: Cdc73 suppresses genome instability by mediating telomere homeostasis
Source: PLoS Genet. 2018 Jan 10;14(1):e1007170. doi: 10.1371/journal.pgen.1007170 (PMC5779705; doi:10.1371/journal.pgen.1007170)
Supplement: S4 Table — (PDF) [file pgen.1007170.s027.pdf]

Supplemental Table 4. Summary of the evidence for the GCR-associated rearrangements in sGCR-containing isolates

Sample (Relevant Genotype)	GCR Junction Description	GCR Junction Evidence[1]
RDKY7964 (wild-type)	No GCR (parental strain)	n.a.
isolate 541 (wild-type)	*de novo* telomere addition	Copy number (Fig. S5)<br>Junction sequence (49 reads; Fig. S5)
isolate 542 (wild-type)	Homology-mediated translocation between chrV L *can1::P_{LEU2}-NAT SUP53* and chrI R *SUP56*	Copy number (Fig. S5)<br>Discordant read pairs (68 and 74 read pairs[2])<br>Junction sequence (Fig. S10a, b)
isolate 543 (wild-type)	*de novo* telomere addition	Copy number (Fig. S5)<br>Junction sequence (67 reads; Fig. S5)
isolate 544 (wild-type)	Homology-mediated translocation between chrV L *can1::P_{LEU2}-NAT YCLWdelta5* fragment and chrX R *YJRWdelta17*	Copy number (Fig. S5)<br>Discordant read pairs (150 read pairs)<br>Junction sequence (Fig. S9c, d)
isolate 545 (wild-type)	*de novo* telomere addition	Copy number (Fig. S5)<br>Junction sequence (112 reads; Fig. S5)
isolate 546 (wild-type)	Homology-mediated translocation between chrV L *can1::P_{LEU2}-NAT YCLWdelta5* fragment and a full-length Ty element somewhere in the genome based on junction-adjacent sequences; this rearrangement does not give rise to copy number changes in uniquely mapping sequences	Copy number (Fig. S5)<br>Junction sequence
	Homology-mediated translocation between the unidentified full-length Ty element and chrVII R *YGRWdelta21*	Copy number (Fig. S5)<br>Junction sequence
isolate 547 (wild-type)	Homology-mediated translocation between chrV L *can1::P_{LEU2}-NAT SUP53* and chrXIV L *tL(CAA)N*	Copy number (Fig. S5)<br>Discordant read pairs (51 and 42 read pairs[2])<br>Junction sequence (Fig. S10c, d)
	Homology-mediated translocation between chrXIV L *YNLCTy2-1* and chrII L *YBLWTy2-1*	Copy number (Fig. S5)
isolate 548 (wild-type)	Homology-mediated inverted duplication between chrV L *can1::P_{LEU2}-NAT YCLWdelta5* fragment and chrV L *YELWdelta1*	Copy number (Fig. S5)<br>Discordant read pairs (94 read pairs)<br>Junction sequence (Fig. S11)
	Homology-mediated translocation between chrV L *YELWdelta1* and a full-length Ty element somewhere in the genome based on junction-adjacent sequences; this rearrangement does not give rise to copy number change in uniquely mapping sequences	Copy number (Fig. S5)<br>Junction sequence
	Homology-mediated translocation between the unidentified full-length Ty element and chrIII R *YCRWdelta11*	Copy number (Fig. S5)
isolate 549 (wild-type)	*de novo* telomere addition	Copy number (Fig. S5)<br>Junction sequence (113 reads; Fig. S5)
isolate 550 (wild-type)	Homology-mediated translocation between chrV L *can1::P_{LEU2}-NAT YCLWdelta5* fragment and chrIII R *YCRWdelta13*	Copy number (Fig. S5)<br>Discordant read pairs (171 read pairs)<br>Junction sequence (Fig. S9e, f)
isolate 551 (wild-type)	*de novo* telomere addition	Copy number (Fig. S5)<br>Junction sequence (93 reads; Fig. S5)
RDKY8407	No GCR (parental strain)	n.a.

Sample (Relevant Genotype)	GCR Junction Description	GCR Junction Evidence[1]
(*cdc73Δ*)		
isolate 301 (*cdc73Δ*)	Microhomology-mediated translocation to subtelomeric sequences of *TEL15R*	Copy number (Fig. S6)<br>Discordant read pairs (6 read pairs)<br>Junction sequence (14 reads; Fig. S6)
isolate 302 (*cdc73Δ*)	Homology-mediated translocation between chrV L *can1::P_{LEU2}-NAT YCLWdelta5* fragment and chrXIII R *YMRWdelta19*	Copy number (Fig. S6)<br>Discordant read pairs (500 read pairs)<br>Junction sequence (Fig. S9a, b)
	The lack of copy number changes on chrXIII and the inability to identify read pairs spanning *YMRWdelta19* on chrXIII suggest that chrXIII also underwent a rearrangement at *YMRWdelta19*; junction adjacent sequences are consistent with HR involving Ty-related elements. No other copy number changes or discordant read pairs were seen.	-
isolate 303 (*cdc73Δ*)	Hairpin-mediated inverted duplication at chrV L *can1::P_{LEU2}-NAT*	Copy number (Fig. S6)<br>Discordant read pairs (1 read pair)<br>Junction sequence (3 reads; Fig S14a, b)
	Homology-mediated translocation between chrV L *YELWdelta1* and chrV R "*YERWdelta27"* an unannotated delta at chrV:449,322-449,631	Copy number (Fig. S6)<br>Discordant read pairs (93 read pairs)<br>Junction sequence (Fig. S13c)
isolate 304 (*cdc73Δ*)	Homology-mediated inverted duplication between chrV L *can1::P_{LEU2}-NAT YCLWdelta5* fragment and chrV L *YELWdelta1*	Copy number (Fig. S6)<br>Discordant read pairs (271 read pairs)<br>Junction sequence (Fig. S11)
	Homology-mediated translocation between chrV L *YELWdelta6* and chrXVI R *YPRWTy1-3*	Copy number (Fig. S6)<br>Junction sequence (Fig. S12m, n)
isolate 305 (*cdc73Δ*)	Homology-mediated inverted duplication between chrV L *can1::P_{LEU2}-NAT YCLWdelta5* fragment and chrV L *YELWdelta1*	Copy number (Fig. S6)<br>Discordant read pairs (406 read pairs)<br>Junction sequence (Fig. S11)
	Homology-mediated translocation between chrV L *YELWdelta1* and chrV L *YELCdelta4* (inversion)	Copy number (Fig. S6)<br>Discordant read pairs (40 read pairs)
	Homology-mediated inverted duplication between chrV L *can1::P_{LEU2}-NAT YCLWdelta5* fragment and chrV L *YELWdelta1*	Copy number (Fig. S6)<br>Discordant read pairs (406 read pairs)<br>Junction sequence (Fig. S11)
	No further copy number changes or discordant read pairs were seen.	-
isolate 306 (*cdc73Δ*)	Homology-mediated inverted duplication between chrV L *can1::P_{LEU2}-NAT YCLWdelta5* fragment and chrV L *YELWdelta1*	Copy number (Fig. S6)<br>Discordant read pairs (110 read pairs)<br>Junction sequence (Fig. S11)
	Homology-mediated translocation between chrV L *YELCdelta4* and chrV R *YERCdelta20*	Copy number (Fig. S6)<br>Discordant read pairs (16 read pairs)<br>Junction sequence (Fig. S12i, j)
isolate 307 (*cdc73Δ*)	Homology-mediated inverted duplication between chrV L *can1::P_{LEU2}-NAT YCLWdelta5* fragment and chrV L *YELWdelta1*	Copy number (Fig. S6)<br>Discordant read pairs (177 read pairs)<br>Junction sequence (Fig. S11)
	Homology-mediated translocation between chrV L *YELWdelta1* and chrV R "*YERWdelta27"* an unannotated delta at chrV:449,322-449,631	Copy number (Fig. S6)<br>Discordant read pairs (30 read pairs)<br>Junction sequence (Fig. S13c)
isolate 308 (*cdc73Δ*)	Homology-mediated inverted duplication between chrV L *can1::P_{LEU2}-NAT YCLWdelta5* fragment and chrV L *YELWdelta1*	Copy number (Fig. S6)<br>Discordant read pairs (103 read pairs)<br>Junction sequence (Fig. S11)

Sample (Relevant Genotype)	GCR Junction Description	GCR Junction Evidence[1]
	Homology-mediated translocation between chrV L *YELCdelta4* and chrV R *YERCdelta20*, mediated by delta/delta recombination	Copy number (Fig. S6)<br>Discordant read pairs (12 read pairs)<br>Junction sequence (Fig. S12i, j)
isolate 309 (*cdc73Δ*)	Homology-mediated translocation between chrV L *can1::*P_{LEU2}*-NAT SUP53* and chrI R *SUP56*	Copy number (Fig. S6)<br>Discordant read pairs (13 and 110 read pairs[2])<br>Junction sequence (Fig. S10a, b)
isolate 310 (*cdc73Δ*)	Homology-mediated inverted duplication between chrV L *can1::*P_{LEU2}*-NAT YCLWdelta5* fragment and chrV L *YELWdelta1*	Copy number (Fig. S6)<br>Discordant read pairs (105 read pairs)<br>Junction sequence (Fig. S11)
	Homology-mediated translocation between chrV L *YELCdelta4* and chrII L *YBLWTy2-1* (*YBLWdelta5*) or *YBLWdelta6*	Copy number (Fig. S6)
isolate 311 (*cdc73Δ*)	Homology-mediated inverted duplication between chrV L *can1::*P_{LEU2}*-NAT YCLWdelta5* fragment and chrV L *YELWdelta1*	Copy number (Fig. S6)<br>Discordant read pairs (118 read pairs)<br>Junction sequence (Fig. S11)
	Homology-mediated translocation between chrV L *YELCdelta4* and an unidentified Ty element. No further copy number changes or discordant read pairs were seen	Copy number (Fig. S6)
RDKY8409 (*cdc73Δ tel1Δ*)	No GCR (parental strain)	n.a.
isolate 321 (*cdc73Δ tel1Δ*)	Hairpin-mediated inverted duplication at chrV L *can1::*P_{LEU2}*-NAT*	Copy number (Fig. S7)<br>Discordant read pairs (36 read pairs)<br>Junction sequence (45 reads; Fig. S14c, d)
	Homology-mediated translocation between chrV L *YELWdelta6* and chrIV R *YDRCTy1-2*	Discordant read pairs (43 read pairs)
	Homology-mediated translocation between chrIV R *YDRWTy2-2* and chrXII R *YLRCTy2-2*	Copy number (Fig. S7)
isolate 322 (*cdc73Δ tel1Δ*)	Hairpin-mediated inverted duplication at chrV L	Copy number (Fig. S7)<br>Discordant read pairs (298 read pairs)<br>Junction sequence (50 reads; Fig. S14e, f)
	Homology-mediated translocation between chrV L *YELWdelta6* and chrXV L *YOLCdelta1*	Copy number (Fig. S7)<br>Discordant read pairs (87 read pairs)<br>Junction sequence (Fig. S12k, l)
isolate 323 (*cdc73Δ tel1Δ*)	Homology-mediated inverted duplication between chrV L *can1::*P_{LEU2}*-NAT YCLWdelta5* fragment and chrV L *YELWdelta1*	Copy number (Fig. S7)<br>Discordant read pairs (242 read pairs)<br>Junction sequence (Fig. S11)
	Homology-mediated translocation between chrV L *YELCdelta4* and chrII L *YBLWdelta2*	Copy number (Fig. S7)<br>Discordant read pairs (51 read pairs)
isolate 324 (*cdc73Δ tel1Δ*)	Homology-mediated inverted duplication between chrV L *can1::*P_{LEU2}*-NAT YCLWdelta5* fragment and chrV L *YELWdelta1*	Copy number (Fig. S7)<br>Discordant read pairs (374 read pairs)<br>Junction sequence (Fig. S11)
	Homology-mediated translocation between chrV L *YELWdelta1* and chrV R "*YERWdelta27"* an unannotated delta at chrV:449,322-449,631	Copy number (Fig. S7)<br>Discordant read pairs (117 read pairs)<br>Junction sequence (Fig. S13c)
isolate 325 (*cdc73Δ tel1Δ*)	Homology-mediated inverted duplication between chrV L *can1::*P_{LEU2}*-NAT YCLWdelta5* fragment and chrV L *YELWdelta1*	Copy number (Fig. S7)<br>Discordant read pairs (265 read pairs)<br>Junction sequence (Fig. S11)
	Homology-mediated translocation between chrV L *tK(CUU)E1* and chrV R *tK(CUU)E2*	Copy number (Fig. S7)<br>Discordant read pairs (157 read pairs)

Sample (Relevant Genotype)	GCR Junction Description	GCR Junction Evidence[1]
isolate 326 (*cdc73Δ tel1Δ*)	Homology-mediated inverted duplication between chrV L *can1::*P_{LEU2}*-NAT YCLWdelta5* fragment and chrV L *YELWdelta1*	Copy number (Fig. S7)<br>Discordant read pairs (422 read pairs)<br>Junction sequence (Fig. S11)
	Homology-mediated translocation between chrV L *YELCdelta4* and chrV R *YERCdelta16*	Copy number (Fig. S7)<br>Discordant read pairs (92 read pairs)<br>Junction sequence (Fig. 12g, h)
isolate 327 (*cdc73Δ tel1Δ*)	Hairpin-mediated inverted duplication at chrV L *can1::*P_{LEU2}*-NAT*	Copy number (Fig. S7)<br>Discordant read pairs (52 read pairs)<br>Junction sequence (64 reads; Fig. 14g, h)
	Homology-mediated translocation between chrV L *YELCdelta4* to chrI R *YARCTy1-1*	Copy number (Fig. S7)
isolate 328 (*cdc73Δ tel1Δ*)	Homology-mediated inverted duplication between chrV L *can1::*P_{LEU2}*-NAT YCLWdelta5* fragment and chrV L *YELWdelta1*	Copy number (Fig. S7)<br>Discordant read pairs (232 read pairs)<br>Junction sequence (Fig. S11)
	Microhomology-mediated translocation to repetitive subtelomeric sequence	Junction sequence (Fig. S7)
isolate 329 (*cdc73Δ tel1Δ*)	Homology-mediated translocation between chrV L *can1::*P_{LEU2}*-NAT YCLWdelta5* fragment and chrXV L *YOLWTy1-1*	Copy number (Fig. S7)<br>Discordant read pairs (32 read pairs)
	Homology-mediated translocation between chrXV L *YOLWTy1* and chrIII L *YCLCdelta1*	Copy number (Fig. S7)<br>Discordant read pairs (11 read pairs)
isolate 330 (*cdc73Δ tel1Δ*)	Homology-mediated inverted duplication between chrV L *can1::*P_{LEU2}*-NAT YCLWdelta5* fragment and chrV L *YELWdelta1*	Copy number (Fig. S7)<br>Discordant read pairs (209 read pairs)<br>Junction sequence (Fig. S11)
	Homology-mediated translocation between chrV L *YELCdelta4* and chrII L *YBLWdelta2*	Copy number (Fig. S7)<br>Discordant read pairs (41 read pairs)<br>Junction sequence (Fig. S12e, f)
isolate 331 (*cdc73Δ tel1Δ*)	Homology-mediated inverted duplication between chrV L *can1::*P_{LEU2}*-NAT YCLWdelta5* fragment and chrV L *YELWdelta1*	Copy number (Fig. S7)<br>Discordant read pairs (130 read pairs)<br>Junction sequence (Fig. S11)
	Homology-mediated translocation between chrV L *YELWdelta1* and chrV R "*YERWdelta27"* an unannotated delta at chrV:449,322-449,631	Copy number (Fig. S7)<br>Discordant read pairs (12 read pairs)<br>Junction sequence (Fig. S13c)
RDKY8411 (*cdc73Δ yku80Δ*)	No GCR (parental strain)	n.a.
isolate 345 (*cdc73Δ yku80Δ*)	Homology-mediated inverted duplication between chrV L *can1::*P_{LEU2}*-NAT YCLWdelta5* fragment and chrV L *YELWdelta1*	Copy number (Fig. S8)<br>Discordant read pairs (63 read pairs)<br>Junction sequence (Fig. S11)
	Homology-mediated translocation between chrV L *YELCdelta4* and chrVIII R *YHRCdelta15*	Discordant read pairs (6 read pairs)
	Disomy for chrXVI (not necessarily GCR-related)	Read depth histograms (Fig. S15)
isolate 346 (*cdc73Δ yku80Δ*)	Homology-mediated inverted duplication between chrV L *can1::*P_{LEU2}*-NAT YCLWdelta5* fragment and chrV L *YELWdelta1*	Copy number (Fig. S8)<br>Discordant read pairs (119 read pairs)<br>Junction sequence (Fig. S11)
	De novo telomere or translocation to telomeric DNA	Junction sequence (Fig. S8)
isolate 347 (*cdc73Δ yku80Δ*)	Homology-mediated inverted duplication between chrV L *can1::*P_{LEU2}*-NAT YCLWdelta5* fragment and chrV L *YELWdelta1*	Copy number (Fig. S8)<br>Discordant read pairs (94 read pairs)<br>Junction sequence (Fig. S11)
	Homology-mediated translocation between chrV L *PAU2* and chrII R *PAU24*	Copy number (Fig. S8)<br>Discordant read pairs (62 read pairs)

Sample (Relevant Genotype)	GCR Junction Description	GCR Junction Evidence[1]
isolate 348 (*cdc73Δ yku80Δ*)	Microhomology-mediated translocation to subtelomeric sequences of *TEL15R*	Copy number (Fig. S8)<br>Junction sequence (71 reads; Fig. S8)
isolate 349 (*cdc73Δ yku80Δ*)	Hairpin-mediated inverted duplication at chrV L *can1::*P_{LEU2}*-NAT*	Copy number (Fig. S8)<br>Discordant read pairs (45 read pairs)<br>Junction sequence (12 reads; Fig. S14i, j)
	Homology-mediated inverted duplication between chrV L *YELCdelta4* and chrII L *YBLWdelta4*	Copy number (Fig. S8)<br>Discordant read pairs (23 read pairs)<br>Junction sequence (Fig. S12a, b)
isolate 350 (*cdc73Δ yku80Δ*)	Homology-mediated inverted duplication between chrV L *can1::*P_{LEU2}*-NAT YCLWdelta5* fragment and chrV L *YELWdelta1*	Copy number (Fig. S8)<br>Discordant read pairs (375 read pairs)<br>Junction sequence (Fig. S11)
	Homology-mediated translocation between chrV L *YELCdelta4* and an unidentified Ty element. No additional copy number changes or discordant read pairs were seen.	Copy number (Fig. S8)
isolate 351 (*cdc73Δ yku80Δ*)	Homology-mediated inverted duplication between chrV L *can1::*P_{LEU2}*-NAT YCLWdelta5* fragment and chrV L *YELWdelta1*	Copy number (Fig. S8)<br>Discordant read pairs (302 read pairs)<br>Junction sequence (Fig. S11)
	Homology-mediated translocation between chrV L *YELCdelta4* and chrII L *YBLWTy2-1*	Copy number (Fig. S8)
isolate 352 (*cdc73Δ yku80Δ*)	Homology-mediated inverted duplication between chrV L *can1::*P_{LEU2}*-NAT YCLWdelta5* fragment and chrV L *YELWdelta1*	Copy number (Fig. S8)<br>Discordant read pairs (293 read pairs)<br>Junction sequence (Fig. S11)
	Homology-mediated inverted duplication between chrV L *YELCdelta4* and chrXII R *YLRCdelta26*	Copy number (Fig. S8)<br>Discordant read pairs (41 read pairs)<br>Junction sequence (Fig. S12c, d)
isolate 353 (*cdc73Δ yku80Δ*)	Homology-mediated inverted duplication between chrV L *can1::*P_{LEU2}*-NAT YCLWdelta5* fragment and chrV L *YELWdelta1*	Copy number (Fig. S8)<br>Discordant read pairs (319 read pairs)<br>Junction sequence (Fig. S11)
	Homology-mediated translocation between chrV L *YELWdelta6* and chrIII R *YCRWdelta13*	Copy number (Fig. S8)<br>Discordant readpairs (546 read pairs)<br>Junction sequence (Fig. S12o, p)
isolate 354 (*cdc73Δ yku80Δ*)	Homology-mediated translocation between chrV L *can1::*P_{LEU2}*-NAT YCLWdelta5* fragment and chrII L *YBLWdelta2*	Copy number (Fig. S8)<br>Discordant read pairs (308 read pairs)<br>Junction sequence (Fig. S9g, h)
	No further copy number changes or discordant read pairs were seen.	-
isolate 355 (*cdc73Δ yku80Δ*)	Homology-mediated inverted duplication between chrV L *can1::*P_{LEU2}*-NAT YCLWdelta5* fragment and chrV L *YELWdelta1*	Copy number (Fig. S8)<br>Discordant read pairs (300 read pairs)<br>Junction sequence (Fig. S11)
	Homology-mediated translocation between chrV L *YELCdelta4* and chrII L *YBLWTy2-1*	Copy number (Fig. S8)

[1]Evidence supporting GCR-related junctions can be derived from whole genome sequencing data in several ways. Frequently, multiple lines of evidence support the identity of individual junctions. Copy number changes, from normalized read depth plots of uniquely mapping regions of the genome, can suggest possible rearrangements, particularly those involving HR between repetitive elements, because GCRs typically duplicate the region of the target chromosome that is joined to GCR chromosome. Discordant read pairs are read pairs where two uniquely mapping reads map to regions consistent with the GCR, but not with reference

chromosome; note that the number of discordant read pairs is influenced by whether the rearrangement is within or adjacent to non-uniquely mapping regions of the genome. Junction sequences can be determined in two ways, depending on the junction. For junctions that generate a novel sequence, such as a deletion or hairpin-mediated inversion, the sequence can be derived from read pairs adjacent to junction-defining discordant read pairs in which one read does not map to the reference and potentially sequences the novel junction. In these cases, the numbers of reads that align to define the novel junction sequence are reported. For junctions mediated by homologies, such as tRNA genes or Ty-related sequences, sequences can be determined by aligning reads that are linked to uniquely mapping regions adjacent to the homologies involved in the junction.

[2]Translocation observed by junction-defining read pairs for the translocation target with both ChrV L (adjacent to P_{LEU2}-*YCLWdelta5* fragment, listed first) and ChrIII L (within the P_{LEU2}-*YCLWdelta5* fragment, listed second).
