# Supplementary material for: Cdc73 suppresses genome instability by mediating telomere homeostasis
Source: PLoS Genet. 2018 Jan 10;14(1):e1007170. doi: 10.1371/journal.pgen.1007170 (PMC5779705; doi:10.1371/journal.pgen.1007170)
Supplement: S5 Table — (PDF) [file pgen.1007170.s028.pdf]

Supplemental Table 5. Effects of overexpressing *RNH1* and *TLC1* on genome instability in the uGCR assay.

	RDKY	***empty vector (pRS314) Can^R $5FOA^R$ Rate*†**	***RNH1 overexpression (pCM184) Can^R $5FOA^R$ Rate*†**	***TLC1 overexpression (pVL2679) Can^R $5FOA^R$ Rate*†**
cdc73Δ	8480	<5.41 [4.25-6.38] x 10^{-9}	2.76 [1.24-5.91] x 10^{-9}	4.27 [3.28-6.57] x 10^{-9}
cdc73Δ tel1Δ	8481	2.06 [1.06-4.77] x 10^{-8}	1.05 [0.80-2.09] x 10^{-8} (0.06)	5.86 [3.42-10.4] x 10^{-9} (0.0007)
cdc73Δ yku80Δ	8482	8.12 [4.96-19.8] x 10^{-8}	6.63 [2.82-10.3] x 10^{-8} (0.13)	5.59 [2.39-7.96] x 10^{-8} (0.47)

†Rate of accumulating Can^R $5FOA^R$ progeny in CSM-Trp media. GCR rates are known to be decreased in CSM media relative to rich media. The numbers in square brackets [ ] are the 95% confidence interval limits. The number in parenthesis is the p-value of the difference in rates of the pCM184 or pVL2679-containing sample relative to the sample containing the empty vector determined using a two-tailed Mann-Whitney U-test.
