# Supplementary material for: Cdc73 suppresses genome instability by mediating telomere homeostasis
Source: PLoS Genet. 2018 Jan 10;14(1):e1007170. doi: 10.1371/journal.pgen.1007170 (PMC5779705; doi:10.1371/journal.pgen.1007170)
Supplement: S6 Table — (PDF) [file pgen.1007170.s029.pdf]

Supplemental Table 6. Effect of *CDC73* truncations on dGCR rates.

	Wild-type *YKU80*		*yku80*Δ Mutation	
Genotype	***RDKY***	***Can^R 5FOA^R Rate*†**	***RDKY***	***Can^R 5FOA^R Rate*†**
Wild-type	7635	8.1 [6.4-15] x 10^{-8} (1)	8339	3.29 [1.5-10] x 10^{-7} (4.1)
cdc73Δ2-91	8358	1.37 [0.8-3.8] x 10^{-7} (1.7)	8369	1.51 [1.0-3.8] x 10^{-7} (1.9)
cdc73Δ2-124	8362	1.42 [1.1-2.7] x10^{-7} (1.7)	8373	1.32 [0.7-4.3] x 10^{-7} (1.6)
cdc73Δ2-229	8357	6.73 [4.0-23] x 10^{-7} (8.3)	8368	1.32 [0.2-3.8] x 10^{-5} (163)
cdc73Δ92-147	8360	4.51 [1.4-9.9] x 10^{-7} (5.6)	8371	6.98 [3.2-16] x 10^{-7} (8.6)
cdc73Δ92-229	8359	1.07 [0.4-11] x 10^{-6} (13.2)	8370	1.09 [0.2-4.5] x10^{-5} (135)
cdc73Δ125-229	8363	1.40 [0.8-3.3] x 10^{-6} (17.3)	8374	7.95 [0.3-14] x 10^{-6} (98.1)
cdc73Δ148-229	8361	6.08 [4.3-16] x 10^{-7} (7.5)	8372	1.21 [0.6-2.6] x 10^{-6} (14.9)
cdc73Δ230-393	8356	1.31 [0.9-2.4] x 10^{-7} (1.6)	8367	2.01 [1.2-6.8] x 10^{-7} (2.5)
cdc73:125-229	8364	2.09 [1.0-3.2] x 10^{-7} (2.6)	8375	2.26 [1.9-4.9] x 10^{-7} (2.8)

†Rate of accumulating Can^R 5FOA^R progeny. The numbers in square brackets [ ] are the 95% confidence interval limits. The number in parenthesis is the fold increase relative to the wild-type dGCR assay.
n.d., not determined.
