# Supplementary material for: Cdc73 suppresses genome instability by mediating telomere homeostasis
Source: PLoS Genet. 2018 Jan 10;14(1):e1007170. doi: 10.1371/journal.pgen.1007170 (PMC5779705; doi:10.1371/journal.pgen.1007170)
Supplement: S7 Table — (PDF) [file pgen.1007170.s030.pdf]

Supplemental Table 7. *S. cerevisiae* strains used in this study.

Name	Relevant Genotype	Reference
RDKY7635	*MATalpha hom3-10 ura3Δ0 leu2Δ0 trp1Δ63 his3Δ200 lyp1::TRP1 cyh2-Q38K iYFR016::P_{MFA1}-LEU2 can1::P_{LEU2}-NAT yel072w::CAN1-URA3*	(Putnam et al. 2016)
RDKY7986	RDKY7635 *cdc73::HIS3*	This study
RDKY8421	RDKY7635 *chl1::G418*	This study
RDKY8063	RDKY7635 *ctr9::HIS3*	This study
RDKY8426	RDKY7635 *dnl4::hph*	This study
RDKY8419	RDKY7635 *exo1::G418*	This study
RDKY8061	RDKY7635 *leo1::HIS3*	This study
RDKY8423	RDKY7635 *mid1::G418*	This study
RDKY7990	RDKY7635 *paf1::HIS3*	This study
RDKY8425	RDKY7635 *rad52::hph*	This study
RDKY7988	RDKY7635 *rtf1::HIS3*	This study
RDKY8172	RDKY7635 *sir2::G418*	This study
RDKY8173	RDKY7635 *sir3::G418*	This study
RDKY8424	RDKY7635 *sir4::G418*	This study
RDKY8340	RDKY7635 *tel1::G418*	This study
RDKY8422	RDKY7635 *thp1::G418*	This study
RDKY8420	RDKY7635 *yku70::G418*	This study
RDKY8339	RDKY7635 *yku80::G418*	This study
RDKY8429	RDKY7635 *cdc73::HIS3 chl1::G418*	This study
RDKY8436	RDKY7635 *cdc73::HIS3 dnl4::G418*	This study
RDKY8428	RDKY7635 *cdc73::HIS3 exo1::G418*	This study
RDKY8431	RDKY7635 *cdc73::HIS3 mid1::G418*	This study
RDKY8435	RDKY7635 *cdc73::HIS3 rad52::G418*	This study
RDKY8432	RDKY7635 *cdc73::HIS3 sir2::G418*	This study
RDKY8433	RDKY7635 *cdc73::HIS3 sir3::G418*	This study
RDKY8434	RDKY7635 *cdc73::HIS3 sir4::G418*	This study
RDKY8324	RDKY7635 *cdc73::HIS3 tel1::G418*	This study
RDKY8440	RDKY7635 *cdc73::HIS3 tel1::G418 dnl4::hph*	This study
RDKY8466	RDKY7635 *cdc73::hph exo1::HIS3 tel1::G418*	This study
RDKY8439	RDKY7635 *cdc73::HIS3 tel1::G418 rad52::hph*	This study
RDKY8468	RDKY7635 *cdc73::hph tel1::G418 yku80::HIS3*	This study
RDKY8430	RDKY7635 *cdc73::HIS3 thp1::G418*	This study
RDKY8427	RDKY7635 *cdc73::HIS3 yku70::G418*	This study
RDKY8323	RDKY7635 *cdc73::HIS3 yku80::G418*	This study
RDKY8438	RDKY7635 *cdc73::HIS3 yku80::G418 dnl4::hph*	This study
RDKY8465	RDKY7635 *cdc73::hph yku80::G418 exo1::HIS3*	This study
RDKY8437	RDKY7635 *cdc73::HIS3 yku80::G418 rad52::hph*	This study
RDKY8327	RDKY7635 *ctr9::HIS3 yku80::G418*	This study
RDKY8328	RDKY7635 *ctr9::HIS3 tel1::G418*	This study
RDKY8463	RDKY7635 *exo1::HIS3 yku80::G418*	This study
RDKY8464	RDKY7635 *exo1::HIS3 tel1::G418*	This study
RDKY8331	RDKY7635 *leo1::HIS3 yku80::G418*	This study
RDKY8332	RDKY7635 *leo1::HIS3 tel1::G418*	This study
RDKY8325	RDKY7635 *paf1::HIS3 yku80::G418*	This study
RDKY8326	RDKY7635 *paf1::HIS3 tel1::G418*	This study
RDKY8329	RDKY7635 *rtf1::HIS3 yku80::G418*	This study
RDKY8330	RDKY7635 *rtf1::HIS3 tel1::G418*	This study

Name	Relevant Genotype	Reference
RDKY8467	RDKY7635 *yku80::HIS3 tel1::G418*	This study
RDKY8355	RDKY7635 CDC73.*hph*	This study
RDKY8356	RDKY7635 *cdc73*Δ230-393.*hph*	This study
RDKY8357	RDKY7635 *cdc73*Δ2-229.*hph*	This study
RDKY8358	RDKY7635 *cdc73*Δ2-91.*hph*	This study
RDKY8359	RDKY7635 *cdc73*Δ92-229.*hph*	This study
RDKY8360	RDKY7635 *cdc73*Δ92-147.*hph*	This study
RDKY8361	RDKY7635 *cdc73*Δ148-229.*hph*	This study
RDKY8362	RDKY7635 *cdc73*Δ2-124.*hph*	This study
RDKY8363	RDKY7635 *cdc73*Δ125-229.*hph*	This study
RDKY8364	RDKY7635 *cdc73*:125-229.*hph*	This study
RDKY8365	RDKY7635 *yku80::HIS3*	This study
RDKY8366	RDKY7635 *yku80::HIS3* CDC73.*hph*	This study
RDKY8367	RDKY7635 *yku80::HIS3 cdc73*Δ230-393.*hph*	This study
RDKY8368	RDKY7635 *yku80::HIS3 cdc73*Δ2-229.*hph*	This study
RDKY8369	RDKY7635 *yku80::HIS3 cdc73*Δ2-91.*hph*	This study
RDKY8370	RDKY7635 *yku80::HIS3 cdc73*Δ92-229.*hph*	This study
RDKY8371	RDKY7635 *yku80::HIS3 cdc73*Δ92-147.*hph*	This study
RDKY8372	RDKY7635 *yku80::HIS3 cdc73*Δ148-229.*hph*	This study
RDKY8373	RDKY7635 *yku80::HIS3 cdc73*Δ2-124.*hph*	This study
RDKY8374	RDKY7635 *yku80::HIS3 cdc73*Δ125-229.*hph*	This study
RDKY8375	RDKY7635 *yku80::HIS3 cdc73*:125-229.*hph*	This study
RDKY8386	RDKY7635 *CDC73-Venus.G418* Nic96-mCherry.*G418*	This study
RDKY8387	RDKY7635 *cdc73*Δ230-393-*Venus.G418* Nic96-mCherry.*G418*	This study
RDKY8388	RDKY7635 *cdc73*Δ2-124-*Venus.G418* Nic96-mCherry.*G418*	This study
RDKY8389	RDKY7635 *cdc73*Δ125-229-*Venus.G418* Nic96-mCherry.*G418*	This study
RDKY8390	RDKY7635 *cdc73*:125-229-*Venus.G418* Nic96-mCherry.*G418*	This study
RDKY8399	RDKY7635 *cdc73::Venus.G418* Nic96-mCherry.*G418*	This study
RDKY8391	RDKY7635 *CDC73-Venus.G418 paf1::HIS3*	This study
RDKY8392	RDKY7635 *CDC73-Venus.G418 rtf1::HIS3*	This study
RDKY8393	RDKY7635 *CDC73-Venus.G418 ctr9::HIS3*	This study
RDKY8394	RDKY7635 *CDC73-Venus.G418 leo1::HIS3*	This study
RDKY8447	RDKY7635 *CDC73-Venus.G418 PAF1-9myc.HIS3*	This study
RDKY8448	RDKY7635 *CDC73-Venus.G418 RTF1-9myc.HIS3*	This study
RDKY8449	RDKY7635 *CDC73-Venus.G418 CTR9-9myc.HIS3*	This study
RDKY8450	RDKY7635 *CDC73-Venus.G418 LEO1-9myc.HIS3*	This study
RDKY8451	RDKY7635 *cdc73*Δ230-393-*Venus.G418 PAF1-9myc.HIS3*	This study
RDKY8452	RDKY7635 *cdc73*Δ230-393-*Venus.G418 RTF1-9myc.HIS3*	This study
RDKY8453	RDKY7635 *cdc73*Δ230-393-*Venus.G418 CTR9-9myc.HIS3*	This study
RDKY8454	RDKY7635 *cdc73*Δ230-393-*Venus.G418 LEO1-9myc.HIS3*	This study
RDKY8455	RDKY7635 *cdc73*Δ2-124-*Venus.G418 PAF1-9myc.HIS3*	This study
RDKY8456	RDKY7635 *cdc73*Δ2-124-*Venus.G418 RTF1-9myc.HIS3*	This study
RDKY8457	RDKY7635 *cdc73*Δ2-124-*Venus.G418 CTR9-9myc.HIS3*	This study
RDKY8458	RDKY7635 *cdc73*Δ2-124-*Venus.G418 LEO1-9myc.HIS3*	This study
RDKY8484	RDKY7635 *cdc73*Δ125-229-*Venus.G418 PAF1-9myc.HIS3*	This study
RDKY8485	RDKY7635 *cdc73*Δ125-229-*Venus.G418 RTF1-9myc.HIS3*	This study
RDKY8486	RDKY7635 *cdc73*Δ125-229-*Venus.G418 CTR9-9myc.HIS3*	This study
RDKY8487	RDKY7635 *cdc73*Δ125-229-*Venus.G418 LEO1-9myc.HIS3*	This study
RDKY8459	RDKY7635 *cdc73*:125-229-*Venus.G418 PAF1-9myc.HIS3*	This study
RDKY8460	RDKY7635 *cdc73*:125-229-*Venus.G418 RTF1-9myc.HIS3*	This study
RDKY8461	RDKY7635 *cdc73*:125-229-*Venus.G418 CTR9-9myc.HIS3*	This study
RDKY8462	RDKY7635 *cdc73*:125-229-*Venus.G418 LEO1-9myc.HIS3*	This study
RDKY7964	*MATalpha hom3-10 ura3Δ0 leu2Δ0 trp1Δ63 his3Δ200 lyp1::TRP1 cyh2-Q38K iYFR016::*P_{MFA1}*-LEU2 can1::*P_{LEU2}*-NAT yel068c::CAN1-URA3*	(Putnam et al. 2016)

Name	Relevant Genotype	Reference
RDKY8407	RDKY7964 *cdc73::HIS3*	This study
RDKY8405	RDKY7964 *tel1::G418*	This study
RDKY8406	RDKY7964 *yku80::HIS3*	This study
RDKY8470	RDKY7964 *cdc73::HIS3 exo1::G418*	This study
RDKY8409	RDKY7964 *cdc73::HIS3 tel1::G418*	This study
RDKY8475	RDKY7964 *cdc73::hph tel1::G418 exo1::HIS3*	This study
RDKY8410	RDKY7964 *cdc73::HIS3 tel1::G418 rad52::hph*	This study
RDKY8411	RDKY7964 *cdc73::HIS3 yku80::G418*	This study
RDKY8474	RDKY7964 *cdc73::hph yku80::G418 exo1::HIS3*	This study
RDKY8412	RDKY7964 *cdc73::HIS3 yku80::G418 rad52::hph*	This study
RDKY8413	RDKY7964 *cdc73::hph yku80::HIS3 tel1::G418*	This study
RDKY8414	RDKY7964 *cdc73::hph yku80::HIS3 tel1::G418*	This study
RDKY8472	RDKY7964 *exo1::HIS3 yku80::G418*	This study
RDKY8473	RDKY7964 *exo1::HIS3 tel1::G418*	This study
RDKY8408	RDKY7964 *yku80::HIS3 tel1::G418*	This study
RDKY6677	*MATa ura3-52 leu2Δ1 trp1Δ63 his3Δ200 lys2ΔBgl hom3-10 ade2Δ1 ade8 can1::hisG yel068c::CAN1/URA3 iYEL072W::hph*	(Putnam et al. 2009)
RDKY6761	RDKY6677 *tel1::HIS3*	(Putnam et al. 2009)
RDKY8006	RDKY6677 *yku80::HIS3*	(Putnam et al. 2014)
RDKY8480	RDKY6677 *cdc73::G418*	This study
RDKY8481	RDKY6677 *cdc73::G418 tel1::HIS3*	This study
RDKY8482	RDKY6677 *cdc73::G418 yku80::HIS3*	This study
BY4741	*MATa leu2Δ0 his3Δ1 ura3Δ0 met15Δ0*	(Brachmann et al. 1998)
RDKY8346	BY4741 *PAF1-Venus.G418*	This study
RDKY8347	BY4741 *RTF1-Venus.G418*	This study
RDKY8348	BY4741 *CTR9-Venus.G418*	This study
RDKY8349	BY4741 *LEO1-Venus.G418*	This study
RDKY8395	BY4741 *PAF1-Venus.G418 cdc73::HIS3*	This study
RDKY8396	BY4741 *RTF1-Venus.G418 cdc73::HIS3*	This study
RDKY8397	BY4741 *CTR9-Venus.G418 cdc73::HIS3*	This study
RDKY8398	BY4741 *LEO1-Venus.G418 cdc73::HIS3*	This study
RDKY8443	BY4741 *PAF1-9myc.HIS3*	This study
RDKY8444	BY4741 *RTF1-9myc.HIS3*	This study
RDKY8445	BY4741 *CTR9-9myc.HIS3*	This study
RDKY8446	BY4741 *LEO1-9myc.HIS3*	This study
RDKY9233	BY4741 *CDC73-Venus.G418 PAF1-9myc.HIS3*	This study
RDKY9234	BY4741 *CDC73-Venus.G418 PAF1-9myc.HIS3 leo1::hph*	This study
RDKY9235	BY4741 *CDC73-Venus.G418 PAF1-9myc.HIS3 rtf1::hph*	This study
RDKY9236	BY4741 *CDC73-Venus.G418 PAF1-9myc.HIS3 ctr9::URA3*	This study
BY4742	*MATalpha leu2Δ0 his3Δ1 ura3Δ0 lys2Δ0*	(Brachmann et al. 1998)
RDKY8230	BY4742 *TELVII-L::URA3*	This study
RDKY8401	BY4742 *TELVII-L::URA3 yku80::G418*	This study
RDKY8402	BY4742 *TELVII-L::URA3 tel1::G418*	This study
RDKY8333	BY4742 *TELVII-L::URA3 cdc73::HIS3*	This study
RDKY8403	BY4742 *TELVII-L::URA3 cdc73::HIS3 yku80::G418*	This study
RDKY8404	BY4742 *TELVII-L::URA3 cdc73::HIS3 tel1::G418*	This study
RDKY8334	BY4742 *TELVII-L::URA3 paf1::HIS3*	This study
RDKY8335	BY4742 *TELVII-L::URA3 ctr9::HIS3*	This study
RDKY8336	BY4742 *TELVII-L::URA3 rtf1::HIS3*	This study
RDKY8337	BY4742 *TELVII-L::URA3 leo1::HIS3*	This study
RDKY8376	BY4742 *TELVII-L::URA3* CDC73.*hph*	This study
RDKY8377	BY4742 *TELVII-L::URA3 cdc73*Δ230-393.*hph*	This study

**Name**	**Relevant Genotype**	**Reference**
RDKY8378	BY4742 *TELVII-L::URA3 cdc73*Δ2-229.*hph*	This study
RDKY8379	BY4742 *TELVII-L::URA3 cdc73*Δ2-91.*hph*	This study
RDKY8380	BY4742 *TELVII-L::URA3 cdc73*Δ92-229.*hph*	This study
RDKY8381	BY4742 *TELVII-L::URA3 cdc73*Δ92-147 *hph*	This study
RDKY8382	BY4742 *TELVII-L::URA3 cdc73*Δ148-229.*hph*	This study
RDKY8383	BY4742 *TELVII-L::URA3 cdc73*Δ2-124.*hph*	This study
RDKY8384	BY4742 *TELVII-L::URA3 cdc73*Δ125-229.*hph*	This study
RDKY8385	BY4742 *TELVII-L::URA3 cdc73*:125-229.*hph*	This study
